# Exploration of Ziziphi Spinosae Semen in Treating Insomnia Based on Network Pharmacology Strategy

**DOI:** 10.1155/2021/9888607

**Published:** 2021-10-28

**Authors:** Gong Feipeng, Xie Luxin, Chen Beili, Yang Songhong, Wu Wenting, Li Junmao, Gong Qianfeng, Zhong Lingyun, Wu Jianxiong

**Affiliations:** ^1^Jiangxi University of Chinese Medicine, Nanchang, China; ^2^Jiangxi Provincial People's Hospital Affiliated to Nanchang University, Nanchang, China; ^3^Tiantai County Food and Drug Testing Center, Taizhou, China

## Abstract

Ziziphi Spinosae Semen (ZSS) is a common natural medicine used to treat insomnia, and to show clearly its method of action, we managed and did an in-depth discussion. Network pharmacology research is very suitable for the analysis of multiple components, multiple targets, and multiple pathways of Traditional Chinese Medicine (TCM). According to the relevant theory, we first carefully collected and screened the active ingredients in ZSS and received 11 active ingredients that may work. The targets going along with these active components were also strongly related to insomnia targets, 108 common genes were identified, and drug-compound-gene symbol-disease visualization network and protein-protein interaction network were constructed. Forty-eight core genes were identified by PPI analysis and subjected to GO functional analysis with KEGG pathway analysis. The results of GO analysis pointed that there were 998 gene ontology items for the treatment of insomnia, including terms of 892 biological processes, 47 cellular components, and 59 molecular functions. It mainly shows the coupling effect and transport mode of some proteins in the biological pathways of ZSS in the treatment of insomnia and explains the mechanism of action through the connection between the target and the cell biomembrane. KEGG enrichment analyzed 19 signaling pathways, which were collectively classified into seven categories. We have identified the potential pathways of ZSS against insomnia and obtained the regulatory relationship between core genes and pathways and know that the same target can be regulated by multiple components at the same time. The results of molecular docking also prove this conclusion. We sought to provide a new analytical approach to explore TCM treatments for diseases using network pharmacology analysis tools.

## 1. Introduction

Insomnia refers to the symptoms of unable to get commonly and regular sleep or difficulty falling asleep, insufficient sleep time, lack of deep sleep, or even sleepless nights. Sleep deprivation is a potential factor for a variety of risk sicknesses. General medicine-based treatments such as cognitive behavioral therapy for insomnia have limitations, and better alternatives have also been searched for [1]. There are many links between insomnia and mental health, of which anxiety and depression may have a bidirectional effect with insomnia, and the problem can no longer be ignored by people [2]. Such people are prone to dizziness and brain swelling, generalized weakness, explosive temper, and irritability in the morning, which have been very harmful to the body for a long time in the past. With the fierce development of social competition, people's life rhythm has become faster. The prevalence and recurrence rates of insomnia remain high, and it has become one of the hot issues in the field of public health.

The effective drugs commonly used clinically are benzodiazepines, but they also have obvious side effects, even increasing the death rate of the elderly [3, 4]. As an alternative to treating insomnia, Chinese medicine has achieved good therapeutic effects and has gradually attracted the attention of clinicians [5]. Ziziphi Spinosae Semen (ZSS) is the dried mature seed of Rhamnaceae plant *Ziziphus jujuba* Mill.var *spinosa* (Bunge) Hu ex H. F. Chou; according to TCM theory, ZSS is a gentle temperament drug that can nourish the heart and benefit the liver, tranquilize the mind, converge sweat, and generate fluid [6]. ZSS is a relatively easy-to-obtain natural medicine. It is found in many Chinese medicine prescriptions for calming and tranquilizing the nerves, and it is most commonly used to treat insomnia [7].

**Figure 1:**
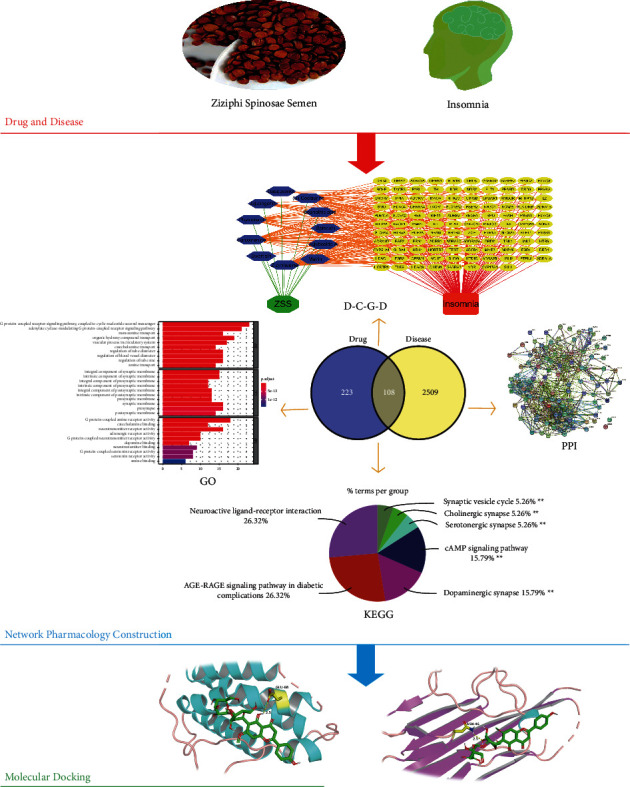
A comprehensive strategy diagram for the study of the mechanism of ZSS acting on insomnia.

Chinese herbal medicine has been used to treat diseases in China for thousands of years. Drugs regulate the immune system or nervous system by eliminating pathogenic factors and strengthening vital energy, so that various organs of the human body can operate normally and maintain the balance between the body and nature. Traditional Chinese herbs, on the other hand, are often multicomponent, multitarget, and multicenter of action. This paper also explains the treatment of insomnia by ZSS with the help of tools and analysis methods (Figure 1).

## 2. Materials and Methods

### 2.1. Screening of Active Compounds

To get the compounds in ZSS directly and effectively, we used the most commonly used specialized Traditional Chinese Medicine Systems Pharmacology Database and Analysis Platform (http://lsp.nwu.edu.cn/tcmsp.php); this platform relatively comprehensively covers the vast majority of TCM compounds' described information, while including the links between related targets and diseases [8, 9]. All ingredients about ZSS were first collected as candidates with the Chinese name “Suanzaoren” as the search term. Afterwards, we introduced these limiting parameters of oral bioavailability (OB) and drug-likeness (DL) according to the classical active ingredient screening rules [10], in which OB refers to the percentage of unmodified drugs that enter the circulatory system after oral administration and can effectively represent the availability of drugs, and OB ≥ 30% of compounds is considered as one of the screening rules for active ingredients [11]. While DL refers to the similarity of compounds to clinically used drugs, the greater DL value represents the possibility of compounds becoming drugs, and molecules with DL ≥ 0.18 are carefully thought about to have better pharmacological effects and are also one of the most commonly used active ingredient screening rules [12], so we started obeying this rule. In addition, as a drug often used by Chinese clinicians, the active substances of traditional Chinese medicine must meet the relevant provisions of the Chinese Pharmacopoeia when prescribing [13]. We also added the active ingredients specified in the Chinese Pharmacopoeia as active compounds.

### 2.2. Identification of Targets and Gene Symbols Associated with ZSS Compounds

The SMILES structural formulas of the compounds were obtained with the ZINC 15 database [14] and TCMSP Platform according to the CAS number, and the SMILES formulas were input into the SwissTargetPrediction (STP; http://www.swisstargetprediction.ch) [15]. We use the information given to extract the protein targets of each ZSS-active compounds and simplify the obtained data to get gene symbols.

### 2.3. Acquisition of Insomnia Gene Targets

In order to ensure that the genes we obtain that are associated with the disease are more comprehensive, we used “Insomnia” as a keyword to search on these two reliable database platforms, respectively, from the GeneCards database (https://www.genecards.org/) and the Online Mendelian Inheritance in Man (OMIM) database (http://www.omim.org/). GeneCards database automatically integrates data from about 125 network-derived genes including genome, transcriptomics, and proteomics, with very powerful functions [16, 17]. The OMIM database can question almost any data on genetic diseases and provide linkage relationships known about causative genes [18, 19]. After summarizing the obtained disease information, it is then screened in detail. Eventually, we obtained genes associated with insomnia.

### 2.4. Drug-Compound-Gene-Disease (D-C-G-D) Network Construction

We first obtained overlapping targets by crossing ZSS-related targets with insomnia-related targets by Venn diagram and then built a visual comprehensive network (D-C-G-D) based on the interaction between drugs (ZSS), compounds, gene symbols, and diseases (insomnia) by Cytoscape software (version 3.8.0) and made the corresponding schematic.

### 2.5. Protein-Protein Interaction (PPI) Network Construction

Discovering and annotating the interaction relationships of all functional targets in proteins enable system-level learning and understanding of the functions of proteins, and protein-protein interaction data were obtained from the STRING database (https://string-db.org/). STRING is commonly used to filter and evaluate relevant data for functional genomics, setting the organism species to *Homo sapiens* (human) before retrieval [20] and then organizing the received data.

### 2.6. Gene Ontology (GO) and Kyoto Encyclopedia of Genes and Genomes (KEGG) Pathway Enrichment

Gene Ontology (GO) enrichment analyses was performed with R software (version 4.0.2), and the entries enriched were determined by the Bioconductor database (http://bioconductor.org/). Kyoto Encyclopedia of Genes and Genomes (KEGG) enrichment analyses were performed using ClueGO plug-in in Cytoscape [21].

### 2.7. Computational Validation of Compound-Target Interactions

As a feasible and modern verification method, molecular docking is a method for drug design through the characteristics of receptors and the interaction mode between receptors and drug molecules and is widely used in the field of drug binding to protein receptors [22]. We selected three active ingredients for docking with their corresponding two targets, for a total of four component-target interactions to validate the prediction results. The protein crystal forms matching up to all targets were obtained from the RCSB Protein Data Bank (PDB) (https://www.rcsb.org). We chose the appropriate protein crystal overall principle to be structurally intact and stable and obtained X-ray crystal structures of TNF (TNF-alpha) and IL-2 (interleukin-2), which have a PDBID of 5 UUI and 1 M4B, respectively. In addition, to further confirm the reliability of the docking results, we also docked these two proteins with their positive inhibitors curcumin and upadacitinib. After processing targets and compounds using PyMOL software (version 1.3), docking work was performed using AutoDock Vina software (version 1.1.2), and the presentation files were processed by AutoDockTools.

## 3. Results

### 3.1. Screening of Active Compounds

We collated the collected data and compared the obtained component names, CAS numbers, and structures one by one to remove some inaccurate information. Nine active ingredients that met the above conditions were obtained by screening according to the previous parameters of OB ≥ 30% and DL ≥ 0.18. In addition, two components, jujuboside A and spinosin, were included in the provisions of Chinese Pharmacopoeia 2020. Studies have shown that saponins and flavonoids are the two most important active components in ZSS [23, 24], the most important of which are jujuboside A and spinosin. Jujuboside A can inhibit cancer cell growth through various mechanisms such as cell cycle arrest, proliferation inhibition, stem cell inhibition, and promotion of aging and can contaminate antitoxins [25, 26], and our analysis results show that there are many intersections of targets associated with it and insomnia targets. Spinosin exerts neuroprotective effects by inhibiting oxidative damage and has a significant antidepressant effect, and there is a great association between spinosin and insomnia [27, 28]. Although the kinetic values of these two components are relatively low, they have very excellent biological activity and are closely related to our study. Finally, all eligible compounds were combined and we obtained 11 fractions as final active compounds (Table 1).

### 3.2. Identification of Targets and Gene Symbols Associated with ZSS Compounds

After collecting related proteins from the STP database and simplifying (probability>0), we obtained 11 components in the ZSS and 331 known target symbols associated with them (Supplementary Table S1).

### 3.3. Acquisition of Known Therapeutic Gene Targets for Insomnia

We merged all target data obtained from the GeneCards and OMIM databases for insomnia-related targets and removed duplicated genes from them, collecting 2617 known insomnia therapeutic targets (Supplementary Table S2).

### 3.4. D-C-G-D Network Construction

**Figure 2:**
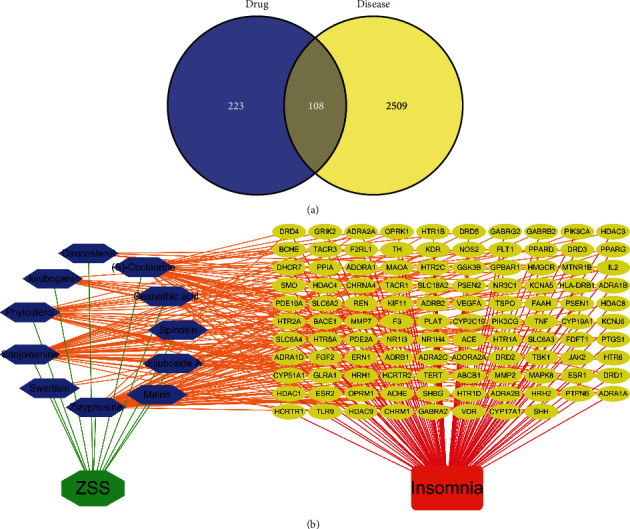
(a) Venn diagram of related targets of ZSS and insomnia. (b) D-C-G-D network. Green and red nodes indicate ZSS and insomnia, respectively. 11 blue nodes represent active ingredients in ZSS, 108 yellow nodes represent overlapping gene symbols between disease and drug, with edges indicating that nodes can interact, red edges indicate the action of insomnia with genes, green edges indicate the interaction of ZSS with active ingredients, and orange edges indicate the interaction of active ingredients with genes.

According to Venn diagram (Figure 2(a)), we can visually see the cross genes common to drugs and diseases, of which 331 drug gene symbols and 2617 disease gene symbols have 108 overlaps. The D-C-G-D network (Figure 2(b)) also clearly shows how ZSS treats insomnia by acting between components and targets, and the details of the D-C-G-D network are shown in Supplementary Table S3.

### 3.5. PPI Network Construction

**Figure 3:**
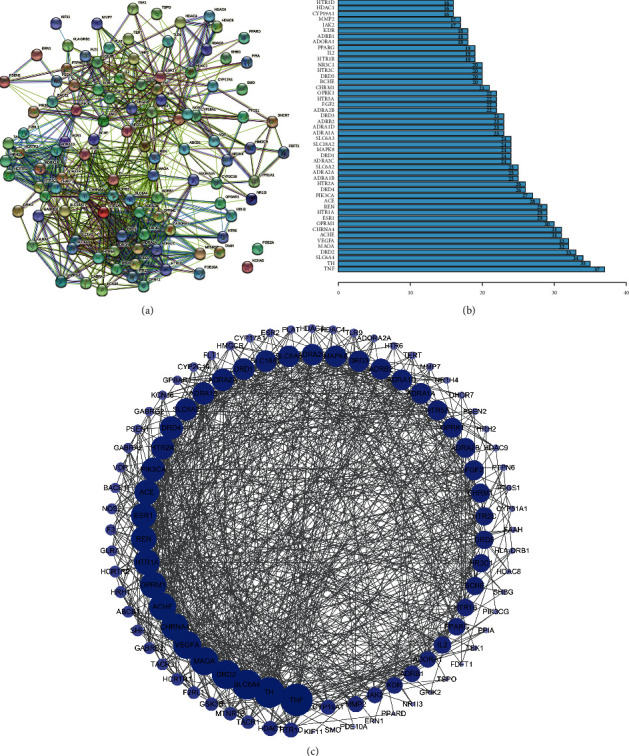
(a) The PPI network obtained from the STRING database platform. (b) The core gene degree barplot diagram. (c) The PPI network arranged according to degree value.

According to the results received by STRING platform, we got the action relationship map among 108 overlapping genes (Figure 3(a)), including two unconnected free genes, which may be important targets of ZSS to treat insomnia. After removing the free genes, we calculated the topological index node degree of 106 genes, which largely points to the degree of association of this gene with other genes. The higher node degree indicates that this gene may be more important, and the details of the specific gene name and its corresponding node degree are given in Supplementary Table S4. In addition, we calculated the mean value of 106 overlapping gene degrees to be 15.05, selected all genes with value greater than the mean value [34], and generated 48 core targets of relatively more important networks with other gene interactions (Figure 3(b)). Moreover, to better highlight the importance of key targets, after processing these related targets using Cytoscape software, it displayed 48 genes with higher values in the inner loop of Figure 3(c). The deeper the blue color and the larger the nodes, the higher the node degree; the line between them represented the interaction. These core targets may be the key genes of ZSS in the treatment of insomnia.

### 3.6. GO and KEGG Pathway Enrichment

**Figure 4:**
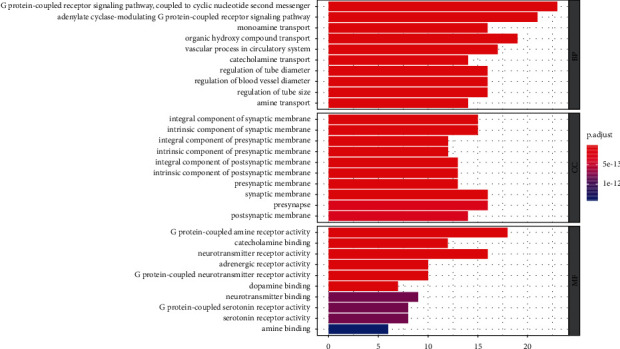
Bar graph of GO function enrichment of overlapping targets. The *Y*-axis represents GO terms. The *X*-axis indicates the number of genes enriched in this pathway. The redder the color, the smaller the p.adjust value; it also indicates the reliability and importance. The bluer the color, the greater the p.adjust value.

GO enrichment analysis (*p* < 0.01) of 48 core target genes was classified and enriched according to three modules: biological process (BP), cellular component (CC), and molecular function (MF), with 998 GO terms enriched, of which the BP term accounted for a relatively high proportion, with 892 GO terms, mainly showing the coupling role and transport mode of some proteins in biological pathways, such as G protein-coupled receptor signaling pathway, coupled to cyclic nucleotide second messenger (GO:0007187), adenylate cyclase-modulating G protein-coupled receptor signaling pathway (GO:0007188), monoamine transport (GO:0015844), and organic hydroxy compound transport (GO:0015850). CC is rich in 47 GO terms and analyzes that these core genes are closely related to those cell biofilms, mainly involving a variety of synaptic membranes, such as integral component of synaptic membrane (GO:0099699), intrinsic component of synaptic membrane (GO:0099240), and integral component of presynaptic membrane (GO:0099056). Similarly, we enriched 59 GO terms for MF, and in MF analysis, we could understand which receptor activities the core genes would affect and the forms of partial protein and target binding, mainly G protein-coupled amine receptor activity (GO:0008227), neurotransmitter receptor activity (GO:0030594), and catecholamine binding (GO:1901338). To present the GO enrichment results more directly, we based on *p*. Adjust intercepts for the top 10 terms from small to large for an abbreviated presentation (Figure 4), respectively, and the detailed results of the specific GO item analysis are given in Supplementary Table S5.

**Figure 5:**
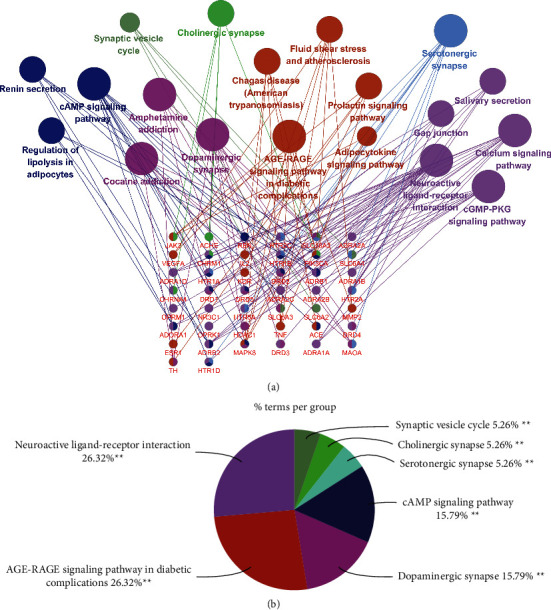
(a) Correlation diagram between core targets and KEGG pathway. The smaller the term p.adjust, the larger the node. Nodes with different colors represent different pathway classifications, and the line between pathway and gene represents the interaction between them. (b) Representative classification of pathways and percentage.

KEGG pathway enrichment analysis can help us further figure out the potential pathways of ZSS against insomnia; after all core genes are entered and after applying *p* value significance selection criteria, 44 genes from all clusters are systematically selected and 19 representative pathways are selected (*p* < 0.01*p* < 0.01), as shown in Figure 5(a), and these pathways are divided into 7 categories by signaling pathway functional clustering analysis, of which neuroactive ligand-receptor interaction (KEGG:04080), AGE-RAGE signaling pathway in diabetic complications (KEGG:04933), dopaminergic synapse (KEGG:04728), cAMP signaling pathway (KEGG:04024), synaptic vesicle cycle (KEGG:04721), serotonergic synapse (KEGG:04726), and cholinergic synapse (KEGG: 04725) each represent a class of biological pathways (Figure 5(b)). Some of these pathways have been shown to be highly connected with insomnia, such as key protein pathways that upregulate neuroactive ligand-receptor interaction signaling that can significantly improve insomnia [35], and some pharmacological treatments for psychiatric disorders also use selective blockade of presynaptic dopamine receptors in the frontal cortex, enhancing dopaminergic transmission, which is consistent with the results of our analysis of modulation of the dopaminergic synapse signaling pathway [36]. All details of the KEGG pathway enrichment analysis are described in Supplementary Table S6.

### 3.7. Computational Validation of Ingredient-Target Interactions

**Figure 6:**
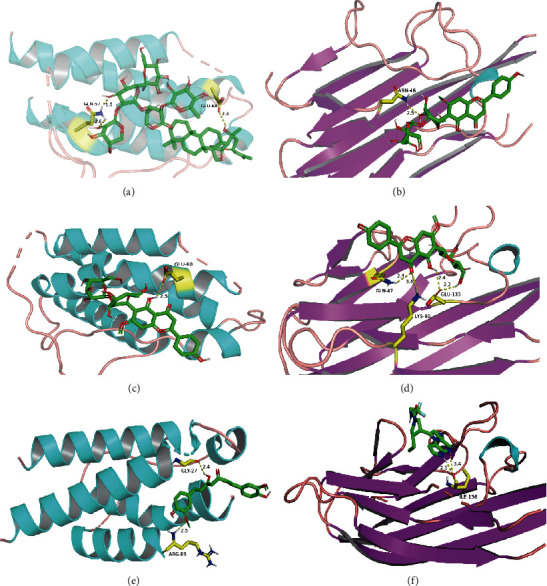
Selected compounds' interactions with the targets. (a) Jujuboside A with IL-2, (b) swertisin with TNF, (c) spinosin with IL-2, (d) spinosin with TNF, (e) curcumin with IL-2, and (f) upadacitinib with TNF. The molecule is represented in a ball-stick model with atoms C, O, and N in green, red, and blue, respectively. Hydrogen bonds are indicated by dashed lines, and the numbers above represent distances in angstroms (Å).

Based on the above prediction and analysis, we selected three classical components, swertisin, jujuboside A, and spinosin, in ZSS for molecular docking with two important proteins, TNF and IL-2, to validate our analysis results. According to the docking results, we know that the binding energy of swertisin to TNF is −6.4 kcal/mol, the binding energy of jujuboside A to IL-2 is −7.8 kcal/mol, and the binding energy of spinosin to TNF and IL-2 is −6.9 and −5.9 kcal/mol, respectively. The lower binding energy between molecules represents that the stronger force binding energy between them (<0 kcal/mol) is carefully thought about being conducive to the binding reaction, which shows that our analysis is accurate and reliable, and the compounds can better act on the related proteins. The binding energy of IL-2 and its inhibitor curcumin is −6.5 kcal/mol, and the binding energy of TNF and the inhibitor upadacitinib is −6.4 kcal/mol. The compound and the inhibitor are docked into the same pocket of the protein, which means our results are more reliable.  Figure 6 shows the specific situation of their combination, and the molecule is represented in a ball-stick model of atoms C, O, and N in green, red, and blue, respectively. Hydrogen bonds are indicated by dashed lines, and the numbers above represent distances in angstroms (Å). Among them, two amino acid residues in IL-2, GLN-57 and GLU-68, form multiple binding locations with jujuboside A (Figure 6(a)), while GLU-68 also binds to spinosin through hydrogen bonding (Figure 6(c)). Three amino acid residues in TNF, GLN-47, LYS-90, and GLU-135-48, form multiple hydrogen bonds with swertisin (Figure 6(b)), and another amino acid residue, ASN-46, forms a hydrogen bond with spinosin (Figure 6(d)). It linked the positive inhibitor curcumin to the two amino acids, GLY-27 and ARG-83, on IL-2 (Figure 6(e)), and upadacitinib forms two hydrogen bonds with the amino acid ILE-136 on TNF (Figure 6(f)). Our analysis shows that ZSS can effectively act on multiple targets such as TNF and IL-2 simultaneously through various components such as swertisin, jujuboside A, and spinosin, which makes the drug molecules affect the protein, and this achieves the effect of treating insomnia.

## 4. Discussion

With the acceleration of people's modern life rhythm, the number of insomnia population showed an increasing trend. Despite developed medical conditions and increasingly diverse means of treating psychiatric disorders, the problem of insomnia still cannot be effectively treated [37, 38]. Monomeric drugs have opened a channel of remission for some patients with neurological diseases, but it is undeniable that the effects of drug resistance and other side effects are not negligible [39, 40]. Most herbs originate from nature with relatively few side effects and adverse effects. ZSS is a pure natural traditional Chinese medicine that has a significant therapeutic effect on insomnia [41, 42], but its application and development have some limitations because of the complex mechanism of action of multiple components and multiple targets of traditional Chinese medicine. In this study, we used network pharmacology theory and related tools to select the active components and related targets of ZSS and linked them with insomnia-related targets to construct a D-C-G-D visual network, which more intuitively showed the specific relationship between drug components and diseases through 108 consensus genes. The results of PPI analysis showed complex interactions between overlapping genes, and 48 core targets were further screened according to the average node degree between them, such as TNF with a high node degree, meaning that one key to ZSS treatment of insomnia may be the inhibition of inflammation *in vivo* [43], which also made our subsequent analysis more prominent.

GO functional analysis makes a simple annotation of gene products, BP enrichment results in the coupling effect and transport mode of proteins in biological pathways, for example, G protein-coupled receptors can mediate the normal function of the brain, of which H3 and H4 receptors are high-affinity receptors in the brain and immune system, respectively, and H3 receptors can regulate histamine and neurotransmitters, which control sleep quality [44]. The results of CC analysis show that intersection proteins or their products take part in the cellular environment, and active biofilm, such as a variety of protrusion membranes, can be utilized as tools to transmit synaptic currents in hypothalamic neurons, triggering arousal and regulating energy metabolism [45]. By MF analysis, we can determine that some protein receptor activities are regulated by drugs, such as neurotransmitter receptor, which is closely related to sleep, of which *γ*-amino butyric acid receptor plays an important role in controlling different vigilance states in the human body [46]. KEGG pathway analysis effectively helps us to associate genomes with cellular, species, and ecosystem functions. According to the results of our analysis, neuroactive ligand-receptor interaction, cAMP signaling pathway, and one of the key pathways to treat insomnia in ZSS, in the pathway, multiple targets such as ADRA1A, ADRA1B, and ADRA1D can be simultaneously regulated by components such as (S)-cointeraction, sanjoinenine, and zizyphusine, and upregulation of neuroactive ligand-receptor interaction has also been an active process for the treatment of insomnia [35]. Besides identifying the specific pathways involved, cluster analysis of these pathways is also unique to our study, such as the cAMP signaling pathway, renin secretion, and dopaminergic synapse; these three pathways function similarly or are more closely linked during ZSS treatment of insomnia. Some protein kinases in the cAMP signaling pathway phosphorylate some specific proteins associated with insomnia, controlling the molecular and intracellular signaling of sleep and wake states [47]. Renin secretion can affect vital active states, and increased activity of the renin-angiotensin system in the brain elevates fluid intake, blood pressure, and resting metabolic rate renin secretion [48]. In addition, antipsychotics may improve psychomotor and routine by releasing dopamine in the prefrontal cortex, which may also involve the dopaminergic synapse signaling pathway [36]. Classification analysis of signaling pathways reveals population differences between different signaling pathways when specific drugs are used to treat diseases. Based on the prediction results, we finally performed in silico molecular docking validation. The tight binding of a variety of small molecule compounds and amino acids on proteins preliminarily showed that our analysis was reliable, and it was also to affirm the feasibility of traditional Chinese medicine, an active compound in ZSS, in the treatment of insomnia.

Complex diseases often have many symptoms, and sometimes, the use of monomeric components is less effective to treat complex diseases and has significant side effects. TCM has been used to treat diseases for thousands of years, with significant results. Meanwhile, the special philosophical ideas of Chinese medicine and the very complex composition of TCM limit its further development, and the rise of network pharmacology brings another idea for the study of TCM. By constructing a drug-target-disease network, higher levels of information can be obtained by calculating the mechanism of simultaneous action of multiple components, multiple targets, and multiple pathways, and then using the construction of visual network charts can enable researchers to quickly and clearly know the mechanism of complex components in the treatment of diseases, which is a very practical new research tool. All of our work is dedicated to establishing a new analytical method with the help of network pharmacology, and besides exploring ZSS for the treatment of insomnia, we also hope to apply it in more traditional Chinese medicine research.

## Figures and Tables

**Table 1:** Final active compounds selected as the details of ZSS in this study.

No.	Molecule name	MF	MW	OB (%)	DL	CAS number	References
1	Ceanothic acid	C30H46O5	486.76	33.41	0.77	21302-79-4	[29]
2	(S)-coclaurine	C17H19NO3	285.37	42.35	0.24	486-39-5	[30]
3	Daucosterol	C35H60O6	576.80	36.91	0.75	474-58-8	[31]
4	Jujubogenin	C30H48O4	472.78	34.96	0.62	54815-36-0	[32]
5	Phytosterol	C29H50O	414.79	36.91	0.75	949109-75-5	[31]
6	Sanjoinenine	C29H35N3O4	489.67	67.27	0.79	107446-80-0	[31]
7	Swertisin	C22H22O10	446.44	31.83	0.75	6991-10-2	[33]
8	Zizyphusine	C20H24NO4	342.45	41.52	0.55	107446-79-7	[29]
9	Mairin	C30H48O3	456.78	55.37	0.78	472-15-1	[31]
10	Jujuboside A	C58H94O26	1207.52	8.03	0.02	55466-04-1	[30]
11	Spinosin	C28H32O15	608.60	6.31	0.72	72063-39-9	[33]

## Data Availability

In this study, the sources are indicated in the text when the third-party data that may be involved are cited, and all datasets presented in this study are included within the article/supplementary materials and can be open to the public.

## Additional Points

*Contribution to the Field.* Ziziphi Spinosae Semen (ZSS) has a unique effect in the field of insomnia and comforting the nerves, and the curative effect is remarkable. ZSS appears in many Chinese doctors' prescriptions for treating insomnia. It is known as the sleeping fruit of the East. However, at present, there are very few studies on how it works in the human body. With the help of network pharmacology, we conducted an association analysis on the important proteins of jujube seed in the treatment of insomnia for the first time and carefully studied the important biological pathways and cell functions that may be involved. Our work effectively makes the theoretical research of traditional Chinese medicine more closely integrated with the actual clinical application.

## Supplementary Materials

Supplementary Materials Table S1: detailed information of related targets and gene symbols of compounds in ZSS. Table S2: the known therapeutic gene targets for insomnia. Table S3: detailed information about the D-C-G-D network. Table S4: overlapping gene symbols and corresponding degree values between disease and drug. Table S5: details of GO enrichment analyses. Table S6: details of KEGG pathway enrichment analyses.
