# Supplementary material for: Exploration of Ziziphi Spinosae Semen in Treating Insomnia Based on Network Pharmacology Strategy
Source: Evid Based Complement Alternat Med. 2021 Oct 28;2021:9888607. doi: 10.1155/2021/9888607 (PMC8568550; doi:10.1155/2021/9888607)
Supplement: Supplementary Materials — Table S1: detailed information of related targets and gene symbols of compounds in ZSS. Table S2: the known therapeutic gene targets for insomnia. Table S3: detailed information about the D-C-G-D network. Table S4: overlapping gene symbols and corresponding degree values between disease and drug. Table S5: details of GO enrichment analyses. Table S6: details of KEGG pathway enrichment analyses. [file 9888607.f1.zip › 9888607.f1/Supplementary Table S1-Detailed information of related targets and gene symbols of compounds in ZSS (1).doc]

| **Detailed information of related targets and gene symbols of compounds in ZSS** | | |
| --- | --- | --- |
| **MolName** | **Target** | **Symbol** |
| Ceanothic acid | DNA polymerase beta (by homology) | POLB |
| Ceanothic acid | Aldo-keto reductase family 1 member B10 | AKR1B10 |
| Ceanothic acid | Prostaglandin E synthase | PTGES |
| Ceanothic acid | SUMO-activating enzyme | SAE1/UBA2 |
| Ceanothic acid | 11-beta-hydroxysteroid dehydrogenase 1 | HSD11B1 |
| Ceanothic acid | Protein-tyrosine phosphatase 1B | PTPN1 |
| Ceanothic acid | Bile acid receptor FXR | NR1H4 |
| Ceanothic acid | DNA topoisomerase II alpha | TOP2A |
| Ceanothic acid | Dual specificity phosphatase Cdc25C | CDC25C |
| Ceanothic acid | Androgen Receptor (by homology) | AR |
| Ceanothic acid | Carboxylesterase 2 | CES2 |
| Ceanothic acid | Prolyl endopeptidase | PREP |
| Ceanothic acid | UDP-glucuronosyltransferase 2B7 | UGT2B7 |
| Ceanothic acid | Dual specificity phosphatase Cdc25A | CDC25A |
| Ceanothic acid | LXR-alpha | NR1H3 |
| Ceanothic acid | Prostanoid EP2 receptor (by homology) | PTGER2 |
| Ceanothic acid | Angiotensin-converting enzyme | ACE |
| Ceanothic acid | CD81 antigen | CD81 |
| Ceanothic acid | Fatty acid-binding protein, liver | FABP1 |
| Ceanothic acid | Delta opioid receptor (by homology) | OPRD1 |
| Ceanothic acid | Hydroxycarboxylic acid receptor 2 | HCAR2 |
| Ceanothic acid | Neprilysin (by homology) | MME |
| Ceanothic acid | Endothelin-converting enzyme 1 | ECE1 |
| Ceanothic acid | Mu opioid receptor (by homology) | OPRM1 |
| Ceanothic acid | Protein kinase C eta | PRKCH |
| Ceanothic acid | Estrogen receptor beta | ESR2 |
| Ceanothic acid | ADAMTS4 | ADAMTS4 |
| Ceanothic acid | Corticosteroid binding globulin | SERPINA6 |
| Ceanothic acid | Testis-specific androgen-binding protein | SHBG |
| Ceanothic acid | Glucose-6-phosphate 1-dehydrogenase | G6PD |
| Ceanothic acid | Cytochrome P450 51 (by homology) | CYP51A1 |
| Ceanothic acid | Cyclooxygenase-2 | PTGS2 |
| Ceanothic acid | G-protein coupled bile acid receptor 1 | GPBAR1 |
| Ceanothic acid | AMP deaminase 2 | AMPD2 |
| Ceanothic acid | Lysosomal protective protein | CTSA |
| Ceanothic acid | Acyl-CoA desaturase | SCD |
| Ceanothic acid | Serine/threonine-protein kinase Chk1 | CHEK1 |
| Ceanothic acid | Serine/threonine-protein kinase WEE1 | WEE1 |
| Ceanothic acid | Prostanoid EP4 receptor | PTGER4 |
| Ceanothic acid | Aldose reductase (by homology) | AKR1B1 |
| Ceanothic acid | Cytosolic phospholipase A2 | PLA2G4A |
| (S)-Coclaurine | Dopamine D2 receptor (by homology) | DRD2 |
| (S)-Coclaurine | Adrenergic receptor beta | ADRB2 |
| (S)-Coclaurine | Beta-1 adrenergic receptor | ADRB1 |
| (S)-Coclaurine | Sigma opioid receptor | SIGMAR1 |
| (S)-Coclaurine | Dopamine D1 receptor | DRD1 |
| (S)-Coclaurine | Thromboxane A2 receptor | TBXA2R |
| (S)-Coclaurine | Beta-3 adrenergic receptor | ADRB3 |
| (S)-Coclaurine | Dopamine D3 receptor | DRD3 |
| (S)-Coclaurine | Dopamine transporter | SLC6A3 |
| (S)-Coclaurine | Alpha-1d adrenergic receptor | ADRA1D |
| (S)-Coclaurine | Alpha-1a adrenergic receptor | ADRA1A |
| (S)-Coclaurine | Dopamine D4 receptor | DRD4 |
| (S)-Coclaurine | Serotonin 1a (5-HT1a) receptor | HTR1A |
| (S)-Coclaurine | Dopamine D5 receptor | DRD5 |
| (S)-Coclaurine | Mu opioid receptor | OPRM1 |
| (S)-Coclaurine | Alpha-1b adrenergic receptor | ADRA1B |
| (S)-Coclaurine | Coagulation factor VII/tissue factor | F3 |
| (S)-Coclaurine | Serotonin 2a (5-HT2a) receptor | HTR2A |
| (S)-Coclaurine | Small conductance calcium-activated potassium channel protein 1 | KCNN1 |
| (S)-Coclaurine | Small conductance calcium-activated potassium channel protein 3 | KCNN3 |
| (S)-Coclaurine | Small conductance calcium-activated potassium channel protein 2 | KCNN2 |
| (S)-Coclaurine | Monoamine oxidase A | MAOA |
| (S)-Coclaurine | Kappa Opioid receptor | OPRK1 |
| (S)-Coclaurine | Serotonin transporter | SLC6A4 |
| (S)-Coclaurine | Putative hydrolase RBBP9 | RBBP9 |
| (S)-Coclaurine | Orexin receptor 2 | HCRTR2 |
| (S)-Coclaurine | Orexin receptor 1 | HCRTR1 |
| (S)-Coclaurine | Acetylcholinesterase | ACHE |
| (S)-Coclaurine | Dipeptidyl peptidase IV | DPP4 |
| (S)-Coclaurine | Serotonin 7 (5-HT7) receptor | HTR7 |
| (S)-Coclaurine | Delta opioid receptor | OPRD1 |
| (S)-Coclaurine | Anti-estrogen binding site (AEBS) | DHCR7 |
| (S)-Coclaurine | Norepinephrine transporter | SLC6A2 |
| (S)-Coclaurine | Multidrug and toxin extrusion protein 1 | SLC47A1 |
| (S)-Coclaurine | Neuronal acetylcholine receptor; alpha4/beta2 | CHRNA4/CHRNB2 |
| (S)-Coclaurine | Serotonin 2c (5-HT2c) receptor | HTR2C |
| (S)-Coclaurine | Neuronal acetylcholine receptor; alpha2/beta4 | CHRNB4/CHRNA2 |
| (S)-Coclaurine | Neuronal acetylcholine receptor; alpha3/beta2 | CHRNA3/CHRNB2 |
| (S)-Coclaurine | Butyrylcholinesterase | BCHE |
| (S)-Coclaurine | Muscarinic acetylcholine receptor M4 | CHRM4 |
| (S)-Coclaurine | HERG | KCNH2 |
| (S)-Coclaurine | Carbonic anhydrase VII | CA7 |
| (S)-Coclaurine | Alpha-2a adrenergic receptor | ADRA2A |
| (S)-Coclaurine | Adrenergic receptor alpha-2 | ADRA2C |
| (S)-Coclaurine | P-glycoprotein 1 | ABCB1 |
| (S)-Coclaurine | Synaptic vesicular amine transporter (by homology) | SLC18A2 |
| (S)-Coclaurine | Tyrosine-protein kinase JAK3 | JAK3 |
| (S)-Coclaurine | Tyrosine-protein kinase JAK1 | JAK1 |
| (S)-Coclaurine | Tyrosine-protein kinase JAK2 | JAK2 |
| (S)-Coclaurine | Tyrosine-protein kinase TYK2 | TYK2 |
| (S)-Coclaurine | Lysine-specific histone demethylase 1 | KDM1A |
| (S)-Coclaurine | Carbonic anhydrase I | CA1 |
| (S)-Coclaurine | Neuronal acetylcholine receptor beta4 | CHRNB4 |
| (S)-Coclaurine | Neuronal acetylcholine receptor alpha3 | CHRNA3 |
| (S)-Coclaurine | Protein tyrosine phosphatase receptor type C-associated protein | PTPRCAP |
| (S)-Coclaurine | Proto-oncogene c-JUN | JUN |
| (S)-Coclaurine | Carbonic anhydrase II | CA2 |
| (S)-Coclaurine | Glutamate receptor ionotropic kainate 2 | GRIK2 |
| (S)-Coclaurine | Carbonic anhydrase IX | CA9 |
| (S)-Coclaurine | Histone deacetylase 1 | HDAC1 |
| (S)-Coclaurine | Rho-associated protein kinase 1 | ROCK1 |
| (S)-Coclaurine | Rho-associated protein kinase 2 | ROCK2 |
| (S)-Coclaurine | Alpha-2b adrenergic receptor | ADRA2B |
| (S)-Coclaurine | Melatonin receptor 1B | MTNR1B |
| Daucosterol | Interleukin-2 | IL2 |
| Daucosterol | Signal transducer and activator of transcription 3 | STAT3 |
| Daucosterol | Gamma-secretase | PSEN2/PSENEN/NCSTN/APH1A/PSEN1/APH1B |
| Daucosterol | Platelet activating factor receptor | PTAFR |
| Daucosterol | Hepatocyte growth factor receptor | MET |
| Daucosterol | Sphingosine 1-phosphate receptor Edg-3 | S1PR3 |
| Daucosterol | Sphingosine 1-phosphate receptor Edg-1 | S1PR1 |
| Daucosterol | Vascular endothelial growth factor receptor 1 | FLT1 |
| Daucosterol | Plasma retinol-binding protein | RBP4 |
| Daucosterol | Protein phosphatase 2C beta | PPM1B |
| Daucosterol | Serine/threonine protein phosphatase PP1-gamma catalytic subunit | PPP1CC |
| Daucosterol | Serine/threonine protein phosphatase 2A, catalytic subunit, alpha isoform | PPP2CA |
| Daucosterol | Serine/threonine protein phosphatase 2A, 56 kDa regulatory subunit, alpha isoform | PPP2R5A |
| Daucosterol | 11-beta-hydroxysteroid dehydrogenase 2 | HSD11B2 |
| Daucosterol | Sphingosine 1-phosphate receptor Edg-8 | S1PR5 |
| Daucosterol | Sphingosine 1-phosphate receptor Edg-6 | S1PR4 |
| Jujubogenin | Phosphodiesterase 10A | PDE10A |
| Jujubogenin | Prostaglandin E synthase | PTGES |
| Jujubogenin | Renin | REN |
| Jujubogenin | Arachidonate 5-lipoxygenase | ALOX5 |
| Jujubogenin | Neurokinin 2 receptor | TACR2 |
| Jujubogenin | Tyrosine-protein kinase BRK | PTK6 |
| Jujubogenin | Bile acid receptor FXR | NR1H4 |
| Jujubogenin | Phospholipase A2 group 1B | PLA2G1B |
| Jujubogenin | EZH2/SUZ12/EED/RBBP7/RBBP4 | EZH2 |
| Jujubogenin | Epoxide hydratase | EPHX2 |
| Jujubogenin | C-C chemokine receptor type 1 | CCR1 |
| Jujubogenin | P2X purinoceptor 3 | P2RX3 |
| Jujubogenin | Pyruvate dehydrogenase kinase isoform 1 | PDK1 |
| Jujubogenin | Phosphodiesterase 5A | PDE5A |
| Jujubogenin | Estrogen-related receptor alpha | ESRRA |
| Jujubogenin | Estrogen-related receptor beta | ESRRB |
| Jujubogenin | Proteinase-activated receptor 1 | F2R |
| Jujubogenin | Matrix metalloproteinase 3 | MMP3 |
| Jujubogenin | Matrix metalloproteinase 1 | MMP1 |
| Jujubogenin | Matrix metalloproteinase 2 | MMP2 |
| Jujubogenin | Matrix metalloproteinase 7 | MMP7 |
| Jujubogenin | Nitric oxide synthase, inducible (by homology) | NOS2 |
| Jujubogenin | Sodium/potassium-transporting ATPase alpha-1 chain | ATP1A1 |
| Jujubogenin | Cyclooxygenase-1 | PTGS1 |
| Jujubogenin | C-X-C chemokine receptor type 3 | CXCR3 |
| Jujubogenin | Carbonic anhydrase II | CA2 |
| Jujubogenin | Anandamide amidohydrolase | FAAH |
| Jujubogenin | Carbonic anhydrase I | CA1 |
| Jujubogenin | c-Jun N-terminal kinase 1 | MAPK8 |
| Phytosterol | Androgen Receptor | AR |
| Phytosterol | HMG-CoA reductase | HMGCR |
| Phytosterol | Cytochrome P450 51 (by homology) | CYP51A1 |
| Phytosterol | Niemann-Pick C1-like protein 1 | NPC1L1 |
| Phytosterol | LXR-alpha | NR1H3 |
| Phytosterol | Cytochrome P450 19A1 | CYP19A1 |
| Phytosterol | Cytochrome P450 17A1 | CYP17A1 |
| Phytosterol | Nuclear receptor ROR-gamma | RORC |
| Phytosterol | Estrogen receptor alpha | ESR1 |
| Phytosterol | Estrogen receptor beta | ESR2 |
| Phytosterol | Sterol regulatory element-binding protein 2 | SREBF2 |
| Phytosterol | Testis-specific androgen-binding protein | SHBG |
| Phytosterol | Norepinephrine transporter | SLC6A2 |
| Phytosterol | Cytochrome P450 2C19 | CYP2C19 |
| Phytosterol | Nuclear receptor ROR-alpha | RORA |
| Phytosterol | Protein-tyrosine phosphatase 1B | PTPN1 |
| Phytosterol | Butyrylcholinesterase | BCHE |
| Phytosterol | Corticosteroid binding globulin | SERPINA6 |
| Phytosterol | Serotonin transporter | SLC6A4 |
| Phytosterol | Muscarinic acetylcholine receptor M2 | CHRM2 |
| Phytosterol | Vitamin D receptor | VDR |
| Phytosterol | Acetylcholinesterase | ACHE |
| Phytosterol | Glucose-6-phosphate 1-dehydrogenase | G6PD |
| Phytosterol | LXR-beta | NR1H2 |
| Phytosterol | Glycine receptor subunit alpha-1 | GLRA1 |
| Phytosterol | Carboxylesterase 2 | CES2 |
| Phytosterol | Prostanoid EP1 receptor (by homology) | PTGER1 |
| Phytosterol | Prostanoid EP2 receptor (by homology) | PTGER2 |
| Phytosterol | 11-beta-hydroxysteroid dehydrogenase 1 | HSD11B1 |
| Phytosterol | Prostaglandin E synthase | PTGES |
| Phytosterol | Dual specificity phosphatase Cdc25A | CDC25A |
| Phytosterol | Peroxisome proliferator-activated receptor alpha | PPARA |
| Phytosterol | Peroxisome proliferator-activated receptor delta | PPARD |
| Phytosterol | Anti-estrogen binding site (AEBS) (by homology) | DHCR7 |
| Phytosterol | Squalene monooxygenase | SQLE |
| Phytosterol | Protein-tyrosine phosphatase 1C | PTPN6 |
| Phytosterol | Nuclear receptor subfamily 1 group I member 3 | NR1I3 |
| Phytosterol | Squalene synthetase (by homology) | FDFT1 |
| Phytosterol | Sigma opioid receptor | SIGMAR1 |
| Phytosterol | Nitric oxide synthase, inducible (by homology) | NOS2 |
| Phytosterol | Glucocorticoid receptor | NR3C1 |
| Phytosterol | Peroxisome proliferator-activated receptor gamma | PPARG |
| Phytosterol | Dual specificity phosphatase Cdc25B | CDC25B |
| Phytosterol | UDP-glucuronosyltransferase 2B7 | UGT2B7 |
| Phytosterol | 11-beta-hydroxysteroid dehydrogenase 2 | HSD11B2 |
| Phytosterol | DNA polymerase beta (by homology) | POLB |
| Sanjoinenine | Galanin receptor 2 (by homology) | GALR2 |
| Sanjoinenine | Matrix metalloproteinase 8 | MMP8 |
| Sanjoinenine | Galanin receptor 1 (by homology) | GALR1 |
| Sanjoinenine | Isocitrate dehydrogenase [NADP] cytoplasmic | IDH1 |
| Sanjoinenine | Sonic hedgehog protein | SHH |
| Sanjoinenine | Serotonin 1a (5-HT1a) receptor | HTR1A |
| Sanjoinenine | Alpha-1d adrenergic receptor | ADRA1D |
| Sanjoinenine | Alpha-1a adrenergic receptor | ADRA1A |
| Sanjoinenine | Alpha-1b adrenergic receptor | ADRA1B |
| Sanjoinenine | Thrombin and coagulation factor X | F10 |
| Sanjoinenine | Mu opioid receptor | OPRM1 |
| Sanjoinenine | Tyrosine-protein kinase receptor FLT3 | FLT3 |
| Sanjoinenine | Cyclin-dependent kinase 2/cyclin A | CDK2/CCNA1/CCNA2 |
| Sanjoinenine | Tyrosine-protein kinase JAK2 | JAK2 |
| Sanjoinenine | Calcitonin gene-related peptide type 1 receptor | CALCRL |
| Sanjoinenine | Carnitine O-palmitoyltransferase 1, liver isoform | CPT1A |
| Sanjoinenine | Carnitine palmitoyltransferase 2 | CPT2 |
| Sanjoinenine | Neurokinin 3 receptor | TACR3 |
| Sanjoinenine | Potassium channel, inwardly rectifying, subfamily J, member 11 | KCNJ11 |
| Sanjoinenine | Oxytocin receptor | OXTR |
| Sanjoinenine | Serotonin transporter | SLC6A4 |
| Sanjoinenine | Acyl coenzyme A:cholesterol acyltransferase 1 | SOAT1 |
| Sanjoinenine | Nitric oxide synthase, inducible | NOS2 |
| Sanjoinenine | Serine/threonine-protein kinase TBK1 | TBK1 |
| Sanjoinenine | c-Jun N-terminal kinase 1 | MAPK8 |
| Sanjoinenine | Neurokinin 1 receptor | TACR1 |
| Sanjoinenine | Calpain 1 | CAPN1/CAPNS2 |
| Sanjoinenine | Neuropeptide Y receptor type 1 | NPY1R |
| Sanjoinenine | Smoothened homolog (by homology) | SMO |
| Sanjoinenine | Bile acid receptor FXR | NR1H4 |
| Sanjoinenine | Neuropeptide Y receptor type 5 | NPY5R |
| Sanjoinenine | Sodium channel protein type IX alpha subunit | SCN9A |
| Sanjoinenine | Orexin receptor 2 | HCRTR2 |
| Sanjoinenine | Orexin receptor 1 (by homology) | HCRTR1 |
| Sanjoinenine | Delta opioid receptor | OPRD1 |
| Sanjoinenine | TGF-beta receptor type I | TGFBR1 |
| Sanjoinenine | Proteasome Macropain subunit MB1 | PSMB5 |
| Sanjoinenine | HLA class II histocompatibility antigen DRB1-1 | HLA-DRB1 |
| Sanjoinenine | Muscarinic acetylcholine receptor M1 | CHRM1 |
| Sanjoinenine | Telomerase reverse transcriptase | TERT |
| Sanjoinenine | Phosphodiesterase 10A | PDE10A |
| Sanjoinenine | Acetyl-CoA carboxylase 2 | ACACB |
| Sanjoinenine | Translocator protein (by homology) | TSPO |
| Sanjoinenine | Legumain | LGMN |
| Sanjoinenine | Bradykinin B1 receptor | BDKRB1 |
| Sanjoinenine | Prostanoid EP1 receptor | PTGER1 |
| Sanjoinenine | Thromboxane A2 receptor | TBXA2R |
| Sanjoinenine | Tyrosine-protein kinase SYK | SYK |
| Sanjoinenine | Alpha-2a adrenergic receptor | ADRA2A |
| Sanjoinenine | Glycine transporter 1 | SLC6A9 |
| Sanjoinenine | C5a anaphylatoxin chemotactic receptor | C5AR1 |
| Sanjoinenine | Bradykinin B2 receptor | BDKRB2 |
| Sanjoinenine | Sphingosine 1-phosphate receptor Edg-3 | S1PR3 |
| Sanjoinenine | Apoptosis regulator Bcl-2 | BCL2 |
| Sanjoinenine | Scavenger receptor class B member 1 (by homology) | SCARB1 |
| Sanjoinenine | Serotonin 7 (5-HT7) receptor | HTR7 |
| Sanjoinenine | Serotonin 6 (5-HT6) receptor | HTR6 |
| Sanjoinenine | Kinesin-like protein 1 | KIF11 |
| Sanjoinenine | Coagulation factor IX | F9 |
| Sanjoinenine | Phosphodiesterase 2A | PDE2A |
| Sanjoinenine | Bromodomain-containing protein 2 | BRD2 |
| Sanjoinenine | Tissue-type plasminogen activator | PLAT |
| Sanjoinenine | Complement factor D | CFD |
| Sanjoinenine | EZH2/SUZ12/EED/RBBP7/RBBP4 | EZH2 |
| Sanjoinenine | Phosphodiesterase 5A | PDE5A |
| Sanjoinenine | Phosphodiesterase 11A | PDE11A |
| Sanjoinenine | Kir3.1/Kir3.4 | KCNJ5/KCNJ3 |
| Sanjoinenine | Kir3.1/Kir3.2 | KCNJ6/KCNJ3 |
| Sanjoinenine | Formyl peptide receptor 1 | FPR1 |
| Sanjoinenine | Phosphodiesterase 9A | PDE9A |
| Sanjoinenine | Lipoxin A4 receptor | FPR2 |
| Sanjoinenine | Vanilloid receptor | TRPV1 |
| Sanjoinenine | Bromodomain-containing protein 4 | BRD4 |
| Sanjoinenine | Vasopressin V2 receptor | AVPR2 |
| Sanjoinenine | Vasopressin V1a receptor | AVPR1A |
| Sanjoinenine | Kappa Opioid receptor | OPRK1 |
| Sanjoinenine | Epoxide hydratase | EPHX2 |
| Sanjoinenine | Muscarinic acetylcholine receptor M3 | CHRM3 |
| Sanjoinenine | Histone deacetylase 3/NCoR1 | NCOR1/HDAC3 |
| Sanjoinenine | Serotonin 2b (5-HT2b) receptor | HTR2B |
| Sanjoinenine | Histamine H1 receptor | HRH1 |
| Sanjoinenine | Chymotrypsin C | CTRC |
| Sanjoinenine | Prolactin-releasing peptide receptor | PRLHR |
| Sanjoinenine | Cyclophilin A | PPIA |
| Sanjoinenine | Lysosomal Pro-X carboxypeptidase | PRCP |
| Sanjoinenine | MAP kinase p38 alpha | MAPK14 |
| Sanjoinenine | G-protein coupled receptor kinase 2 | GRK2 |
| Sanjoinenine | Dopamine D1 receptor | DRD1 |
| Sanjoinenine | Geranylgeranyl transferase type I | PGGT1B/FNTA |
| Sanjoinenine | 3-phosphoinositide dependent protein kinase-1 | PDPK1 |
| Sanjoinenine | Ileal bile acid transporter | SLC10A2 |
| Sanjoinenine | LDL-associated phospholipase A2 | PLA2G7 |
| Sanjoinenine | Voltage-gated potassium channel subunit Kv1.5 | KCNA5 |
| Sanjoinenine | P2X purinoceptor 7 | P2RX7 |
| Sanjoinenine | Secreted frizzled-related protein 1 | SFRP1 |
| Sanjoinenine | Type-1 angiotensin II receptor | AGTR1 |
| Sanjoinenine | Cathepsin (V and K) | CTSV |
| Sanjoinenine | MAP kinase p38 beta | MAPK11 |
| Sanjoinenine | Proteinase-activated receptor 2 | F2RL1 |
| Sanjoinenine | Cytochrome P450 19A1 | CYP19A1 |
| Swertisin | Aldose reductase | AKR1B1 |
| Swertisin | Heat shock protein HSP 90-alpha | HSP90AA1 |
| Swertisin | Interleukin-2 | IL2 |
| Swertisin | Carbonic anhydrase XII | CA12 |
| Swertisin | TNF-alpha | TNF |
| Zizyphusine | Neuronal acetylcholine receptor; alpha4/beta2 | CHRNA4/CHRNB2 |
| Zizyphusine | Dopamine D2 receptor (by homology) | DRD2 |
| Zizyphusine | Serotonin 2b (5-HT2b) receptor | HTR2B |
| Zizyphusine | Serotonin 1a (5-HT1a) receptor | HTR1A |
| Zizyphusine | Dopamine D3 receptor | DRD3 |
| Zizyphusine | Serotonin 6 (5-HT6) receptor | HTR6 |
| Zizyphusine | Dopamine D1 receptor | DRD1 |
| Zizyphusine | Serotonin 7 (5-HT7) receptor | HTR7 |
| Zizyphusine | Protein tyrosine phosphatase receptor type C-associated protein | PTPRCAP |
| Zizyphusine | Alpha-1a adrenergic receptor | ADRA1A |
| Zizyphusine | Serotonin 2a (5-HT2a) receptor | HTR2A |
| Zizyphusine | Serotonin 5a (5-HT5a) receptor | HTR5A |
| Zizyphusine | Mu opioid receptor | OPRM1 |
| Zizyphusine | Alpha-1d adrenergic receptor | ADRA1D |
| Zizyphusine | Dopamine D5 receptor | DRD5 |
| Zizyphusine | Serotonin 1b (5-HT1b) receptor | HTR1B |
| Zizyphusine | Dopamine D4 receptor | DRD4 |
| Zizyphusine | Alpha-1b adrenergic receptor | ADRA1B |
| Zizyphusine | Serotonin 2c (5-HT2c) receptor | HTR2C |
| Zizyphusine | Alpha-2a adrenergic receptor | ADRA2A |
| Zizyphusine | Adrenergic receptor alpha-2 | ADRA2C |
| Zizyphusine | Serotonin 1d (5-HT1d) receptor | HTR1D |
| Zizyphusine | Serotonin transporter | SLC6A4 |
| Zizyphusine | Histamine H2 receptor | HRH2 |
| Zizyphusine | Alpha-2b adrenergic receptor | ADRA2B |
| Zizyphusine | Beta-1 adrenergic receptor | ADRB1 |
| Zizyphusine | Tyrosine 3-hydroxylase | TH |
| Zizyphusine | Translocator protein | TSPO |
| Zizyphusine | Dopamine transporter | SLC6A3 |
| Zizyphusine | HERG | KCNH2 |
| Zizyphusine | Arachidonate 12-lipoxygenase | ALOX12 |
| Zizyphusine | Rho-associated protein kinase 2 | ROCK2 |
| Zizyphusine | Gamma-secretase | PSEN2 /PSENEN /NCSTN /APH1A /PSEN1 /APH1B |
| Zizyphusine | Serine/threonine-protein kinase/endoribonuclease IRE1 | ERN1 |
| Zizyphusine | Heat shock protein HSP 90-beta | HSP90AB1 |
| Zizyphusine | Sigma opioid receptor (by homology) | SIGMAR1 |
| Zizyphusine | Glucagon receptor | GCGR |
| Zizyphusine | Vanilloid receptor | TRPV1 |
| Zizyphusine | Glycogen synthase kinase-3 beta | GSK3B |
| Zizyphusine | Protein kinase C alpha | PRKCA |
| Zizyphusine | Protein kinase C beta | PRKCB |
| Zizyphusine | Macrophage colony stimulating factor receptor | CSF1R |
| Zizyphusine | P-glycoprotein 1 | ABCB1 |
| Zizyphusine | Adrenergic receptor beta | ADRB2 |
| Zizyphusine | Cathepsin (B and K) | CTSB |
| Zizyphusine | Peptide deformylase mitochondrial | PDF |
| Zizyphusine | Macrophage migration inhibitory factor | MIF |
| Zizyphusine | Nuclear receptor ROR-gamma | RORC |
| Zizyphusine | Thrombin and coagulation factor X | F10 |
| Zizyphusine | Dual specificity protein phosphatase 3 | DUSP3 |
| Zizyphusine | Aminopeptidase N | ANPEP |
| Zizyphusine | Vascular endothelial growth factor receptor 2 | KDR |
| Zizyphusine | Serine/threonine-protein kinase Aurora-A | AURKA |
| Zizyphusine | Platelet-derived growth factor receptor beta | PDGFRB |
| Zizyphusine | Stem cell growth factor receptor | KIT |
| Zizyphusine | Vascular endothelial growth factor receptor 3 | FLT4 |
| Zizyphusine | Tyrosine-protein kinase receptor FLT3 | FLT3 |
| Zizyphusine | Platelet-derived growth factor receptor alpha | PDGFRA |
| Zizyphusine | Nitric oxide synthase, inducible | NOS2 |
| Zizyphusine | Matrix metalloproteinase 13 | MMP13 |
| Zizyphusine | Matrix metalloproteinase 2 | MMP2 |
| Zizyphusine | Adenosine A1 receptor | ADORA1 |
| Zizyphusine | Adenosine A2a receptor | ADORA2A |
| Zizyphusine | Adenosine A3 receptor | ADORA3 |
| Zizyphusine | Cyclin-dependent kinase 2/cyclin A | CDK2 CCNA1 CCNA2 |
| Zizyphusine | Interleukin-8 receptor B | CXCR2 |
| Zizyphusine | Estradiol 17-beta-dehydrogenase 2 | HSD17B2 |
| Zizyphusine | Glycogen synthase kinase-3 alpha | GSK3A |
| Zizyphusine | Estradiol 17-beta-dehydrogenase 1 | HSD17B1 |
| Zizyphusine | Tankyrase-2 | TNKS2 |
| Zizyphusine | Tankyrase-1 | TNKS |
| Zizyphusine | Cyclin-dependent kinase 5/CDK5 activator 1 | CDK5R1 CDK5 |
| Zizyphusine | Cyclin-dependent kinase 2/cyclin E1 | CCNE1 CDK2 |
| Zizyphusine | PI3-kinase p110-alpha/p85-alpha | PIK3CA PIK3R1 |
| Zizyphusine | Matrix metalloproteinase 3 | MMP3 |
| Zizyphusine | PI3-kinase p110-alpha subunit | PIK3CA |
| Zizyphusine | MAP kinase signal-integrating kinase 2 | MKNK2 |
| Zizyphusine | TGF-beta receptor type I | TGFBR1 |
| Zizyphusine | Epidermal growth factor receptor erbB1 | EGFR |
| Zizyphusine | Protein kinase C gamma (by homology) | PRKCG |
| Zizyphusine | Matrix metalloproteinase 1 | MMP1 |
| Zizyphusine | LDL-associated phospholipase A2 | PLA2G7 |
| Zizyphusine | Glutaminyl-peptide cyclotransferase | QPCT |
| Zizyphusine | Matrix metalloproteinase 8 | MMP8 |
| Zizyphusine | MAP kinase-activated protein kinase 2 | MAPKAPK2 |
| Zizyphusine | PI3-kinase p110-gamma subunit | PIK3CG |
| Zizyphusine | Hepatocyte growth factor receptor | MET |
| Zizyphusine | Beta-secretase 1 | BACE1 |
| Zizyphusine | Prostaglandin E synthase | PTGES |
| Zizyphusine | Arachidonate 15-lipoxygenase | ALOX15 |
| Zizyphusine | Phosphodiesterase 5A | PDE5A |
| Zizyphusine | Estradiol 17-beta-dehydrogenase 3 | HSD17B3 |
| Zizyphusine | Acid ceramidase (by homology) | ASAH1 |
| Zizyphusine | Histone deacetylase 5 | HDAC5 |
| Zizyphusine | Histone deacetylase 7 | HDAC7 |
| Zizyphusine | Histone deacetylase 8 | HDAC8 |
| Zizyphusine | Histone deacetylase 4 | HDAC4 |
| Zizyphusine | Matrix metalloproteinase 7 | MMP7 |
| Zizyphusine | Histone deacetylase 9 | HDAC9 |
| Zizyphusine | Matrix metalloproteinase 12 | MMP12 |
| Mairin | DNA polymerase beta (by homology) | POLB |
| Mairin | Aldo-keto reductase family 1 member B10 | AKR1B10 |
| Mairin | SUMO-activating enzyme | SAE1/UBA2 |
| Mairin | 11-beta-hydroxysteroid dehydrogenase 1 | HSD11B1 |
| Mairin | Protein-tyrosine phosphatase 1B | PTPN1 |
| Mairin | Prostaglandin E synthase | PTGES |
| Mairin | Bile acid receptor FXR | NR1H4 |
| Mairin | T-cell protein-tyrosine phosphatase | PTPN2 |
| Mairin | Dual specificity phosphatase Cdc25C | CDC25C |
| Mairin | G-protein coupled bile acid receptor 1 | GPBAR1 |
| Mairin | DNA topoisomerase II alpha | TOP2A |
| Mairin | Nuclear receptor ROR-gamma | RORC |
| Mairin | Receptor-type tyrosine-protein phosphatase F (LAR) | PTPRF |
| Mairin | Low molecular weight phosphotyrosine protein phosphatase | ACP1 |
| Mairin | Androgen Receptor | AR |
| Mairin | Phospholipase A2 group 1B | PLA2G1B |
| Mairin | Dual specificity phosphatase Cdc25B | CDC25B |
| Mairin | Dual specificity phosphatase Cdc25A | CDC25A |
| Mairin | Carboxylesterase 2 | CES2 |
| Mairin | Phosphodiesterase 4D | PDE4D |
| Mairin | Fatty acid-binding protein, liver | FABP1 |
| Mairin | Vitamin D receptor | VDR |
| Mairin | UDP-glucuronosyltransferase 2B7 | UGT2B7 |
| Mairin | Peroxisome proliferator-activated receptor gamma | PPARG |
| Mairin | Acyl-CoA desaturase | SCD |
| Mairin | Corticosteroid binding globulin | SERPINA6 |
| Mairin | Testis-specific androgen-binding protein | SHBG |
| Mairin | Glucose-6-phosphate 1-dehydrogenase | G6PD |
| Mairin | Cytochrome P450 51 (by homology) | CYP51A1 |
| Mairin | 5-lipoxygenase activating protein | ALOX5AP |
| Mairin | Cytochrome P450 19A1 | CYP19A1 |
| Mairin | Estradiol 17-beta-dehydrogenase 3 | HSD17B3 |
| Mairin | GABA-B receptor (by homology) | GABBR1 |
| Mairin | Protein kinase C eta | PRKCH |
| Mairin | 11-beta-hydroxysteroid dehydrogenase 2 | HSD11B2 |
| Mairin | Protein-tyrosine phosphatase 2C | PTPN11 |
| Mairin | Telomerase reverse transcriptase | TERT |
| Mairin | DNA topoisomerase I | TOP1 |
| Mairin | Niemann-Pick C1-like protein 1 | NPC1L1 |
| Mairin | Sigma opioid receptor | SIGMAR1 |
| Mairin | Cytochrome P450 17A1 | CYP17A1 |
| Mairin | GABA A receptor alpha-2/beta-2/gamma-2 | GABRA2 /GABRB2 /GABRG2 |
| Mairin | Cyclooxygenase-2 | PTGS2 |
| Mairin | p53-binding protein Mdm-2 | MDM2 |
| Mairin | Toll-like receptor (TLR7/TLR9) | TLR9 |
| Mairin | Fatty acid binding protein intestinal | FABP2 |
| Mairin | CD81 antigen | CD81 |
| Mairin | Protein-tyrosine phosphatase 1C | PTPN6 |
| Mairin | Prostanoid EP2 receptor (by homology) | PTGER2 |
| Mairin | LXR-alpha | NR1H3 |
| Jujuboside A | Platelet activating factor receptor | PTAFR |
| Jujuboside A | Protein phosphatase 2C beta | PPM1B |
| Jujuboside A | Protein-tyrosine phosphatase 1B | PTPN1 |
| Jujuboside A | Serine/threonine protein phosphatase PP1-gamma catalytic subunit | PPP1CC |
| Jujuboside A | Serine/threonine protein phosphatase 2A, catalytic subunit, alpha isoform | PPP2CA |
| Jujuboside A | Serine/threonine protein phosphatase 2A, 56 kDa regulatory subunit, alpha isoform | PPP2R5A |
| Jujuboside A | Interleukin-2 | IL2 |
| Jujuboside A | Vascular endothelial growth factor A | VEGFA |
| Jujuboside A | Acidic fibroblast growth factor | FGF1 |
| Jujuboside A | Basic fibroblast growth factor | FGF2 |
| Jujuboside A | Heparanase | HPSE |
| Spinosin | Aldose reductase | AKR1B1 |
| Spinosin | TNF-alpha | TNF |
| Spinosin | Heat shock protein HSP 90-alpha | HSP90AA1 |
| Spinosin | Interleukin-2 | IL2 |
| Spinosin | Xanthine dehydrogenase | XDH |
