# Supplementary material for: Exploration of Ziziphi Spinosae Semen in Treating Insomnia Based on Network Pharmacology Strategy
Source: Evid Based Complement Alternat Med. 2021 Oct 28;2021:9888607. doi: 10.1155/2021/9888607 (PMC8568550; doi:10.1155/2021/9888607)
Supplement: Supplementary Materials — Table S1: detailed information of related targets and gene symbols of compounds in ZSS. Table S2: the known therapeutic gene targets for insomnia. Table S3: detailed information about the D-C-G-D network. Table S4: overlapping gene symbols and corresponding degree values between disease and drug. Table S5: details of GO enrichment analyses. Table S6: details of KEGG pathway enrichment analyses. [file 9888607.f1.zip › 9888607.f1/Supplementary Table S2-The known therapeutic gene targets for Insomnia (1).doc]

**The known therapeutic gene targets for Insomnia**

**gene targets**

PRNP

SNCA

HCRT

HTT

IL6

MAPT

PER2

CSF3R

IL10RA

GALNT2

PRND

DSPS

GFAP

SERPINA3

RLS1

APP

DCTN1

MSMB

SPRN

PRODH

ERCC6

MIRLET7I

ERI3

TXNDC9

CLOCK

HLA-DQB1

HLA-DRB1

LRRK2

PRKN

PARK7

SLC6A4

PINK1

GRIN2B

RLS7

FMR1

CRP

TSPO

SLC2A3

ABCB11

HMBS

CPOX

NAGS

CRY1

APOE

CYP2B6

MEIS1

PER3

GABRB3

GSS

HTR2A

UCHL1

HTRA2

SYNJ1

DNAJC6

PODXL

VPS13C

MTNR1B

LIMK1

TUBB3

NR1H4

ABCB4

MEN1

SLC25A13

SLC12A3

ELN

RFC2

FARS2

CLCNKB

GTF2IRD1

GTF2I

BAZ1B

ATP8B1

TBL2

CLIP2

ZNF365

QARS1

FRAXA

RLS2

BDNF

LEP

C12orf57

ALAD

THRB

DRD4

RLS4

RLS3

RLS5

RLS6

RLS8

HCRTR2

PACRG

PSEN2

STAG3

ALB

MECP2

DHX30

GHRL

MLXIPL

AHDC1

MTNR1A

OPN4

ASMT

LOC110806262

HTR1A

TNF

MAP2

JAK2

CHEK2

GALT

DRD5

PMM2

RAD54L

MAP1B

BRDT

CDK20

MCM9

VPS13B

HIVEP1

INSL6

SCAPER

SGO2

ADHD1

LOC107032825

ADHD2

ADHD3

ADHD4

HCRTR1

CDKN1C

MIR132

MIR29C

LOC100505549

PINK1-AS

LOC109461479

NRCLP2

HTR2C

IL1B

BTBD9

GABRA1

EPO

PSEN1

CRH

TCF4

ENO2

TH

DRD2

MAOA

CYP3A4

ARNTL

SATB2

IL10

MAOB

RBFOX1

ADORA2A

SLC1A3

GRIN2A

SOD1

CSNK1D

EDN1

GSK3B

HTR3A

PPARGC1A

OPRM1

TPH2

HRH1

NPAS2

DRD1

IGF1

CEP152

GRM5

NTF3

TP53

CASP3

NPY

ATXN3

HIF1A

IL17A

SLC6A1

AANAT

MIR182

CSNK1E

NR1D1

CRY2

FBXL3

INS

CXCL10

ATXN7

NPSR1

DOCK3

ASXL3

PRL

ACE

YWHAQ

CD59

MBP

ADIPOQ

C9orf72

TCHH

EP300

ATN1

CHRM1

CREB1

CYCS

ATF6

HSPA8

ERN1

IL1A

CCL5

PRRT1

TPH1

SLC2A13

PPIG

GRIN1

SNAP25

TBP

CLIP1

FOXP2

ETAA1

REN

CYP2D6

GPT

NMT1

MSRA

CYP2E1

TTF2

SNCAIP

HDAC9

YWHAG

COMT

TARDBP

CXCL13

CXCL8

PSMD9

RASA2

ZBTB38

SLC6A3

FDFT1

HBA1

MEF2C

MAP2K5

GLO1

FOXP1

PTPRD

ADGRL3

CASC16

SLC18A2

MOG

PRPH

CAT

EIF2AK3

HSPA5

ATF4

DDIT3

XBP1

EIF2S1

TLR4

GRIA1

TNFRSF1A

PML

SLC22A6

PIGK

UBR5

IFT57

XK

MNT

SLC2A4RG

NOP2

CPO

NPIPA5

NEDD4L

P2RY2

MTUS2

PRIMA1

CNTNAP2

CACNA1C

POMC

FTL

PHOX2B

HMOX1

GAD1

ABCB1

NGF

RARB

CD4

CYP17A1

VCP

SLC1A2

GDNF

DLG4

STX1A

AVP

GRN

MC4R

CES1

TACR1

AFP

NGFR

S100B

IL17RA

GRIK1

CHMP2B

ATXN1

TAC1

PDYN

PPP1R1B

BCL11B

CNTF

DCTN2

BICD2

OXT

MAPRE3

STMN2

TMEM106B

IGLON5

ACTR3C

FHIP1B

SLC6A2

GRIK2

VEGFA

GNAO1

PAX8

SMARCC2

LAMB2

KMT2A

EDC3

BSCL2

ALMS1

DPH1

MYT1L

MED12L

TANC2

ADAM10

NOTCH1

GRIN2D

MAPK3

FYN

CAV1

C1QA

CR2

C1QB

C1QC

STIP1

HSPA1A

MIR342

TTR

DRD3

IFNA1

ADRA1A

DDC

DBH

CAMK2A

GCH1

NR4A2

SYT1

SCN2A

SLC9A6

NKX2-1

HNMT

TFAP2B

ANK3

ATXN2

ARX

TSHB

MED13

DEAF1

TCF20

C12orf4

PIK3C2A

CNR1

NDUFS4

SST

FGFR3

AKT1

CREBBP

TLR3

CHAT

UBE3A

NRXN1

RUNX2

WFS1

TAF1

IDUA

OCA2

VPS13A

SLC25A22

UBTF

LIN28B

PSMC3

BVES

PLCD3

RFX3

HEXIM1

ACBD4

FMNL1

DCAKD

CNIH2

TMUB2

WDR27

ASB16-AS1

LINC02210

LINC01122

LRRC37A4P

MEIS1-AS2

LIN28B-AS1

LINC01798

ENSG00000260793

ENSG00000239268

ENSG00000262372

RPP40P2

ENSG00000273925

ENSG00000285733

ENSG00000266918

LOC105379109

lnc-SPRED2-19

ENSG00000273919

RNU7-147P

ENSG00000287722

HSALNG0015594

piR-32677-423

NONHSAG044482.2

ENSG00000230355

LOC100419513

MMP9

ICAM1

GSR

AGT

IL18

TMEM132E

HTR5A

FGF12

EBF3

TLN2

ADAMTS14

FBXO15

NAALADL2

PLLP

CPQ

TMC5

L3MBTL4

TUSC1

LINC02288

LOC101927066

ACHE

OPRK1

MTHFR

ADORA1

ADRA2B

BAK1

ASXL1

FOS

NOS1

BCHE

TXNRD2

CACNA1A

CTSZ

HRH3

MGRN1

PRTG

SPP2

PROSER1

BSPH1

NR3C2

GABRA2

VIP

SLC17A5

CYP19A1

CACNA1B

FAAH

TTN

HTR1D

GABRA4

HTR1B

HTR4

TRH

MMP2

PPARG

NOS3

CASP1

SERPINE1

B2M

YWHAB

CD40LG

IRF7

FECH

VDAC1

ALAS2

CHKB

UROD

VCAM1

CPT1B

GALE

KCNJ10

HLA-G

ALAS1

NPPB

RETN

THBD

PPOX

HIP1

HSPA4

UROS

HLA-E

FLVCR2

GCG

TPPP3

H2AC18

CCK

ESR1

F2

GAPDH

SLCO1B1

SDHB

BACE1

CPS1

IL2

IGFBP3

MIR146A

GABRG2

MAGEL2

MAP2K1

CDH2

TACR3

MAPKAPK3

HDAC3

CYLD

STAT6

PTPN6

PRKDC

MVK

PTH1R

MST1R

SERPINC1

LRP1

GNAI2

FES

ATP2B2

CACNA1D

NDUFS3

PPARD

RORB

RASGRP1

FURIN

IRF8

MAD1L1

ITPR3

FANCL

GNAT1

DCC

DDR1

AMT

C1S

DAG1

TCF12

PRKAR2A

SLC25A20

PARN

NT5C2

RPA1

GPC3

HLA-B

CTNND1

TRPS1

MMAB

TGFBI

POLG

PDE2A

PNPO

FOXC2

DLK1

GRM4

HTR1F

HMGA2

GFM1

ADARB1

B3GAT3

CHD4

NLGN1

TET2

SORL1

SALL1

PDE4B

PAFAH1B1

PBRM1

MYL3

PTPRS

DAXX

DAB1

B3GAT1

C8B

KDM4B

IL9

NFIB

GMPPB

EFNA5

CLPB

CITED2

UBE3B

TAPBP

NADK

SRR

SLC39A8

PAM

MLF1

RREB1

RIPK4

SH3GL1

MYO1C

HOXB1

SLC33A1

ARFGAP1

ADPGK

FOXF1

CTNND2

DGKI

LTC4S

MAN1A2

HACE1

HYAL2

HS6ST1

KIF2A

LRPPRC

EFTUD2

FANCE

CNNM2

CLK3

DIAPH3

DLG2

AK5

ACTR3

ELAVL2

TERF1

VARS2

VRK2

USP4

UBA7

POLR2J

MTA3

PRIM1

PRPF6

SMAD5

RHCG

PGC

P2RY6

RAB1B

RPL4

RPS13

RPL10A

KLF7

TNFRSF6B

LTBR

MICB

DNAH11

UBE2Z

PPA2

PRPF8

MLLT10

RELT

SEMA3F

ARAP1

AGAP1

CCT7

HOXB9

GUCA1B

IGF2BP1

CACNA2D3

GDF3

CYB561

ACTR1A

ADI1

ARAP3

EMG1

NBEAL2

NCAPD2

NAB2

TRAIP

SSR1

PRPF4B

RGS19

SKAP1

PHB2

RTEL1

HOXB2

RAB6A

RARRES1

TNNI3K

ANO6

BAZ2A

CD276

CA10

IP6K3

KSR2

HOXB5

LMO4

MAML1

NEGR1

GLIS3

DEF6

CNKSR2

CTDSP2

CXXC4

CYP2W1

AAGAB

ARIH2

DAP

UBR2

TAF11

UHRF1

WNK2

UNC5D

TEAD3

TIPIN

SP2

SMG6

MMRN1

PPIP5K2

SLC25A39

PLA2R1

NPAS3

PCDH15

RAB11FIP5

RRN3

RGS16

RBM5

SAFB

ZMYND8

COG5

BAZ2B

LRRTM4

LEMD2

GPRC5B

HBP1

LY75

DIS3L

ADAP1

CDK5RAP3

RNF130

TSR1

TRAF3IP1

PLCL1

PLCH1

PHF2

PPP1R10

COPS7A

KLHDC8B

LSAMP

MAN2A2

HIGD1A

HYAL3

INHBE

NDFIP1

BRAT1

CDK5RAP1

DNAH8

BANK1

ASCC3

BYSL

ARFRP1

ARHGAP10

CHCHD3

ENPP6

NAT8

TCEA2

KMT5A

RANBP3

ZNF76

USP49

UCKL1

WARS1

ZPBP

SYT14

STAU1

SMYD4

PDXDC1

OLFM4

PCDH1

PCDH9

NTAN1

ORAI2

PAN2

MDGA1

HOXB3

RASGRF2

ZFAND3

DNAJC1

BARX1

LECT2

MFSD1

HEXIM2

IFRD2

MED27

FAM172A

BRPF3

CNTN5

CPPED1

CUL9

EIF4E3

ZNF384

MON1A

NAV1

TMEM161B

RBM6

WDR81

TRPC7

YTHDF1

ZGPAT

TAF8

POMGNT2

SNAPC5

PCDHB4

RHOU

SGSM2

NOL4

MED20

RASGEF1B

ANKS1A

FRS3

HEMK1

GPR22

HS6ST3

KCTD10

LRWD1

FBXL17

GPATCH8

GPR139

GPR162

EXOC6B

CLSTN3

ALPK2

ADCK5

BEGAIN

ARHGEF10L

CCDC68

CSMD3

TM9SF4

WDR6

SPSB2

STOML1

PRICKLE4

SDK1

PCDHB11

MLF2

QRICH1

QSOX2

SEC11C

SCAMP5

MDGA2

KCNMB2

RASGEF1C

RBM4B

PTMS

ZDHHC21

CD302

FIBCD1

CCDC148

ALS2CL

MON1B

ZWILCH

TUSC2

UHRF1BP1

VPS8

PSD4

PDRG1

MYO1H

MLN

RC3H2

SLC35B4

ANKRD52

COX19

COPZ2

HHLA2

MFSD6

NICN1

GMEB2

DUS3L

CYB561D2

ALKBH4

PURG

VWC2

WBP1

YPEL4

TENM2

PRDM15

PCMTD2

PIANP

PCDHB5

SCRN2

SCFD2

NKAIN2

R3HDM2

RAB6C

SNX29

DBF4B

C2CD2

CCDC12

GIN1

FIZ1

GPR146

ACTL8

CCDC106

ELFN1

TMEM219

ZNF580

ZNF585B

ZNF628

WDSUB1

SNRNP48

TMEM86B

PCDHGB4

PCDHGC4

PRKRIP1

OAZ3

PHF14

PCDHB12

PCDHB3

PCDHGC5

OR9Q1

P3H3

RAP1GAP2

MED19

NRARP

ZBTB9

EXD3

C16orf72

WDR25

WDR17

WDR90

PCDHGA2

MUC21

PCDHB13

PATJ

RELL2

C7orf50

CCSER1

FAM50B

FBXO41

FAM222B

CYLC1

ZNF784

MRM2

ZNF616

TTC36

VWC2L

PCDHGB3

PCDHGA9

OR5V1

PRSS55

RTL1

TOMM6

FAM166A

ARMC12

ZNF524

ZNRF4

TMEM64

TEX29

PSORS1C1

PCDHGB6

PCDHGA4

NOL4L

PRR15L

C15orf61

C3orf62

BRINP3

LRMDA

FAM180B

GOLGA6A

EFCAB13

CCDC167

CBWD2

EIPR1

TENT4B

PLPP6

METTL16

PUM3

SAMD5

DELE1

ZNF841

C3orf70

MARCHF7

DUSP29

VMAC

DMAC1

IHO1

LHFPL6

ZNF865

SMIM4

SFTA2

NEMP1

NAA80

INKA1

MACIR

SKIDA1

INSYN1

GASK1A

FAM169B

BICRAL

CCDC160

ARIH2OS

LINC02694

DELEC1

EEF1AKMT3

YJU2

IQCJ-SCHIP1

SOX2-OT

RSKR

MIR433

FPGT-TNNI3K

C3orf84

LBHD1

MIR127

LINC00479

LINC00523

GGNBP1

TMEM78

NPIPA2

MIR9-3

RBM14-RBM4

MIR136

MIR339

MIR431

LRRC53

TSPY26P

SCARNA12

MIR337

MIR340

HOXB-AS3

MIR493

LOC284009

LINC01140

TUSC7

C20orf204

HOXB-AS1

LINC00112

LINC00917

TCAM1P

MEIS1-AS3

MIR9-3HG

TMEM161B-AS1

NOL4L-DT

SNORD16

SAMMSON

IQCH-AS1

MAP3K14-AS1

LINC00458

C1RL-AS1

OR7E14P

MEG9

SNORD18A

AGAP2-AS1

DPP9-AS1

IRAIN

IQCM

IPO9-AS1

LINC01524

IGKV1OR2-108

HOXB-AS4

LINC00333

LINC00534

LINC01016

CASC22

UBL7-AS1

TET2-AS1

SPAG5-AS1

PRKAR2A-AS1

PTPRD-AS1

MIR647

PCDHB19P

LINC00989

PABPC1P2

SATB1-AS1

HOXB-AS2

LINC00378

ALMS1P1

TCF4-AS1

LOC283856

SMIM32

STRIT1

PKD1P6

SEMA3F-AS1

KCNMB2-AS1

LINC00558

LINC00602

MIR1297

LINC02801

LINC02055

FMNL1-DT

BTBD9-AS1

LOC100507477

TTC36-AS1

VWC2L-IT1

LOC100505915

MRPL45P2

LINC01812

MIR548AU

LINC00550

LINC00648

LINC01360

KLF7-IT1

LINC01392

LOC730100

SOX1-OT

LINC01799

MIR548A1HG

MIR548AI

MIR548Y

MYOSLID

LINC01065

ARAP1-AS1

LINC02147

LINC02461

HNRNPA1P48

NFE2L1-DT

BAK1P1

LOC100630923

LOC101927450

SP2-DT

ENSG00000240207

SBNO1-AS1

RPS2P47

ZFAND2A-DT

LINC01861

HNRNPA1P16

KRT18P24

LOC100289561

ENSG00000256568

ENSG00000266601

ENSG00000272505

ENSG00000251259

ENSG00000254239

ENSG00000255310

ENSG00000256967

ENSG00000260478

ENSG00000261238

ENSG00000263345

ENSG00000264701

ENSG00000267505

ENSG00000258472

ENSG00000260604

ENSG00000263050

ENSG00000271904

ENSG00000250049

ENSG00000224505

ENSG00000226266

ENSG00000230454

ENSG00000232306

ENSG00000214919

ENSG00000224079

ENSG00000225146

ENSG00000233293

ENSG00000234997

ENSG00000237838

ENSG00000223725

ENSG00000232937

ENSG00000233175

ENSG00000215841

ENSG00000233891

LOC105371795

LOC112268269

ENSG00000240440

PPIAP13

PDE2A-AS1

PKD1P3

MIR7111

LINC01982

OR5AZ1P

RPL35AP9

RSL24D1P6

MYOSLID-AS1

ENSG00000245768

ENSG00000245156

DNM1P33

GAPDHP72

LINC02238

MAPK8IP1P2

LINC02815

CARM1P1

CICP4

EEF1A1P11

MTND4P14

RN7SL809P

RN7SL608P

RNU5A-2P

ENSG00000258317

ENSG00000260289

ENSG00000264019

ENSG00000267571

ENSG00000271955

ENSG00000272374

ENSG00000251095

ENSG00000261351

ENSG00000267255

ENSG00000267344

ENSG00000269807

ENSG00000269918

ENSG00000285367

ENSG00000260850

ENSG00000261261

ENSG00000267360

ENSG00000248373

ENSG00000258526

ENSG00000267030

ENSG00000269892

ENSG00000271778

ENSG00000272247

ENSG00000236545

ENSG00000238280

ENSG00000233417

ENSG00000228322

ENSG00000231460

ENSG00000234902

OR5BA1P

RNU6-829P

RNU4-32P

RNU6-1082P

ENSG00000245261

BDH2P1

LPGAT1P1

EXTL2P1

LOC105371253

MRPS36P5

RNA5SP459

ENSG00000250576

ENSG00000257002

ENSG00000259995

ENSG00000263655

ENSG00000263798

ENSG00000283240

ENSG00000255041

ENSG00000260349

ENSG00000267769

ENSG00000272121

ENSG00000273291

ENSG00000249602

ENSG00000249815

ENSG00000250039

ENSG00000250567

ENSG00000263244

ENSG00000264174

ENSG00000265994

ENSG00000285708

ENSG00000286044

ENSG00000286863

ENSG00000248601

ENSG00000249959

ENSG00000252193

ENSG00000257729

ENSG00000267223

ENSG00000276728

ENSG00000197585

ENSG00000224342

ENSG00000228737

ENSG00000231421

ENSG00000234273

ENSG00000236744

ENSG00000238277

ENSG00000233420

ENSG00000224152

ENSG00000227935

ENSG00000228714

ENSG00000229066

ENSG00000237324

ENSG00000206805

ENSG00000207371

ENSG00000219410

ENSG00000233894

ENSG00000237473

ENSG00000237720

ENSG00000238685

LOC105375861

LOC105372143

ENSG00000239991

MIR6074

PCDH8P1

RNU7-88P

RPS25P7

RPS27AP16

RPS3AP22

RNU6-351P

RNU6-367P

RNU7-105P

RPL21P61

RPL7P20

RNU4-58P

ENSG00000247853

DNAJA1P1

ATP5MFP6

KRT17P3

LINC02496

GEMIN8P2

LOC105370224

TERF1P4

RN7SL761P

RN7SL782P

RN7SL831P

RNA5SP167

ENSG00000266222

ENSG00000272848

ENSG00000275163

ENSG00000283538

ENSG00000285082

ENSG00000286192

ENSG00000251170

ENSG00000271399

ENSG00000283638

ENSG00000254171

ENSG00000284977

ENSG00000252136

ENSG00000257124

ENSG00000284418

ENSG00000200072

ENSG00000229603

ENSG00000176349

ENSG00000228998

ENSG00000232597

ENSG00000124593

ENSG00000237371

ENSG00000237532

LOC105375341

LOC644456

SOD1P2

lnc-ENTPD8-2

lnc-GPR27-16

lnc-MGST1-7

lnc-PRR15L-1

lnc-RASA1-19

RN7SKP122

RN7SL259P

RN7SL401P

RPS10P12

RNU6-956P

RNU6ATAC6P

RPL6P25

RN7SKP157

RNU6-578P

RPL26P31

NOP56P2

ENSG00000287669

ENSG00000288000

HSALNG0113871

lnc-GLIS3-2

lnc-SALL1-2

lnc-SALL1-9

lnc-CCSAP-6

lnc-FANCL-6

lnc-MDH1B-8

lnc-CCDC68-4

lnc-KIAA0141-4

BUB3P1

BTBD7P2

ECM1P2

HEAT2

NAPGP2

RNU1-29P

ENSG00000284705

ENSG00000286225

ENSG00000286908

ENSG00000273399

ENSG00000285638

ENSG00000260103

ENSG00000260838

ENSG00000271749

ENSG00000284720

ENSG00000232068

ENSG00000207922

ENSG00000228391

ENSG00000237137

ENSG00000238694

LOC100420430

LOC646058

lnc-C2CD2-3

lnc-FER-11

lnc-NEMP1-1

lnc-NEMP1-2

lnc-PANK3-22

lnc-PDE2A-2

lnc-PIK3C2A-1

lnc-STAT6-1

lnc-UHRF1-3

HSALNG0035799

HSALNG0055594

PHBP21

RNU6-1296P

ENSG00000287640

ENSG00000287870

ENSG00000288038

lnc-ZNF296-6

lnc-ZNF784-1

HSALNG0026758

HSALNG0047872

HSALNG0019920

HSALNG0030171

HSALNG0043357-002

HSALNG0043364

HSALNG0044010

HSALNG0049833

HSALNG0088908

HSALNG0111389

HSALNG0116820

NONHSAG010333.2

piR-51137-090

HSALNG0069708

HSALNG0069709

HSALNG0069710

HSALNG0088906

HSALNG0111390

HSALNG0113899

NONHSAG011408.2

lnc-METTL16-2

lnc-METTL16-3

lnc-PNPO-1

lnc-RRN3-1

lnc-SALL1-7

lnc-SALL1-8

RF00017-4493

RF00017-4745

RF00017-4746

RF00017-4912

RF00017-5990

HSALNG0103759

HSALNG0109783

lnc-DMRTA1-27

lnc-MAD1L1-6

lnc-NAB2-1

lnc-NMT1-1-001

lnc-PAX8-2

lnc-SGSM2-2

RF00017-4943

RF00017-4944

RF00017-4977

HSALNG0069934

HSALNG0129581

lnc-C20orf203-2

lnc-EIPR1-11

lnc-HDDC3-3

lnc-KIAA0141-5

lnc-LRP1-1

lnc-MAN2A2-1

lnc-NCKIPSD-1

lnc-NOXA1-1

lnc-NTF3-3

lnc-OLFM4-1

lnc-SPRED2-20

lnc-VRK2-17

MK280269-057

MN297160

NONHSAG023944.2-002

piR-30832-008

piR-49120

piR-56005

RF00017-1633

RF00017-2422

RF00017-5033

RF00017-5037

RF00017-5441

RF00017-6018

LOC105369333

LOC105370667

ENSG00000285191

ENSG00000287543

ENSG00000287180

ENSG00000251473

ENSG00000253480

ENSG00000276807

ENSG00000278428

HSALNG0011267-002

HSALNG0012539

LOC126987

LOC107986989

LOC105379189

lnc-PCDH20-22

lnc-PRSS55-4

HSALNG0026288

HSALNG0044024

HSALNG0045074

HSALNG0047148

HSALNG0055593

HSALNG0060168

SGO1P2

lnc-ZFAND3-9

lnc-C15orf59-3

HE856054

HSALNG0011272

HSALNG0030771

HSALNG0036145

HSALNG0044025

HSALNG0025921

HSALNG0053899

HSALNG0075231

HSALNG0113292

HSALNG0113293

HSALNG0116827-001

HSALNG0116821

HSALNG0117050

HSALNG0117053

HSALNG0117094-001

HSALNG0117096

HSALNG0127690

NONHSAG017383.2

piR-32129

piR-32214-596

HSALNG0097330

HSALNG0111383

lnc-ASTN2-5

NONHSAG005377.2

NONHSAG048512.2

piR-43976

piR-46736

lnc-ECT2-5

piR-56037-024

piR-59412-008

RF00017-4696

RF00017-7462

RF00237-002

HSALNG0084677

HSALNG0123439

lnc-IQCH-2

lnc-IQCH-3

lnc-TDRD3-11

piR-31937-163

piR-37656-066

piR-48759-125

piR-51085

RF00017-3011

RF00066-035

HSALNG0076596

HSALNG0116814

lnc-TTC36-2

NONHSAG017392.2

NONHSAG029641.2

NONHSAG032319.2

piR-33422-275

piR-57460-563

RF00017-2184

RF00017-5996

piR-57133-219

piR-59850

RF00017-7134

LOC105371261

LOC105375025

LOC105375433

LOC105373567

LOC101927400

LOC107984425

LOC107984777

ENSG00000279856

ENSG00000275250

ENSG00000280451

ENSG00000286653

ENSG00000268473

ENSG00000270248

ENSG00000270342

ENSG00000270976

ENSG00000278965

ENSG00000219391

ENSG00000213247

ENSG00000228429

ENSG00000217135

HSALNG0004493

HSALNG0004569

HSALNG0014571

LOC105378808

LOC105376244

LOC105371857

lnc-FER-10

lnc-PURG-2

lnc-TDRD3-9

lnc-UQCC2-1

HSALNG0034708

HSALNG0040182-002

HSALNG0043892

HSALNG0049542

HSALNG0053900

HSALNG0055790

ENSG00000288085

HSALNG0015972

HSALNG0015595

HSALNG0011277

HSALNG0025726

HSALNG0025727

HSALNG0025920

HSALNG0043949

HSALNG0049864

HSALNG0050060

HSALNG0053897-002

HSALNG0018714

HSALNG0025460

HSALNG0040188

HSALNG0044017

HSALNG0044359

HSALNG0046381

HSALNG0060409

HSALNG0060411

HSALNG0060413

HSALNG0018049

HSALNG0049217

HSALNG0049578

HSALNG0060162

HSALNG0120815

MK280144-505

NONHSAG029010.2-001

piR-32214-084

piR-41306-230

HSALNG0069671

HSALNG0075408

HSALNG0084326

HSALNG0087542

HSALNG0097483

HSALNG0120632

piR-35672-002

lnc-IZUMO3-2

lnc-PTN-5

RF00017-4946

RF00017-8101

RF00026-985

lnc-ARIH2-3

lnc-CXXC4-5

lnc-NOL4-3

lnc-SLC35E2-1

lnc-SLC3A2-4

lnc-TDRD3-12

MN309174-298

piR-34163-018

piR-50308-547

piR-50437-234

piR-50443-358

HSALNG0069672

HSALNG0070098-001

HSALNG0074031

HSALNG0087541

HSALNG0093893

HSALNG0093895

HSALNG0097465

HSALNG0108033

HSALNG0115519

HSALNG0117094-002

HSALNG0117394

hsa-miR-5095-426

lnc-HS6ST1-8

NONHSAG043779.2

piR-31199-053

piR-39135

piR-60051-012

RF00017-080

RF00017-2423

RF00994-853

LOC105371298

LOC105372043

ENSG00000283043

ENSG00000258358

ENSG00000230987

HSALNG0004471

HSALNG0018052-002

HSALNG0043891

HSALNG0015970

HSALNG0015971

HSALNG0015973

HSALNG0049053

HSALNG0018052-001

piR-50357-130

HSALNG0101017-001

NONHSAG026010.2

piR-38051-302

hsa-miR-5095-368

piR-41525-286

piR-58297-011

KDR

NTRK2

VDR

NTRK3

FTH1

ALDH2

PRKG1

NR3C1

ADRB2

CHRNA4

EPAS1

THRA

SERPINA1

NTRK1

ADRB1

FANCA

TFRC

PIK3CG

IGF2

SLC11A2

KCNJ6

ADRA2A

ISL1

FKBP5

PTH

SCN8A

ADRA2C

LMX1B

GRM7

IGF2R

GNAL

CHRNA7

ARRB2

ACO1

FLNC

HTR3B

KIF5B

GNB5

FHIT

FADS1

FADS2

ADAMTS1

TDO2

STS

PNMT

NEU1

KNG1

CORO1A

DCLK1

AVPR1B

HAMP

IREB2

HTR6

GIT1

CNTFR

ADH1B

BAIAP2

BMP6

CARTPT

CDH13

VAMP2

SYP

NTF4

KCNA4

ITGA1

GRIK4

IL16

HAPLN1

KHDRBS1

DLGAP1

ADAMTS2

DAGLA

MEIS2

SPRED1

TBR1

SYN3

PICK1

AS3MT

AFF2

LIN7A

HTR1E

KCNIP1

NFIL3

FGD1

CHAF1B

TRAPPC6B

NTS

KLK11

DCDC2

IMMP2L

KTN1

MAGED2

KIAA0319

NLGN4X

AUTS2

DACT1

SLC9A9

BRWD1

CALY

FTMT

GPER1

CADPS2

DAZAP2

ANKK1

ADAMTS6

CHMP7

NXPH1

NAV2

MTMR7

TMX3

TRIB2

TOX3

DPPA2

CSN2

CSMD2

TBC1D9

WDR83

PCDHA3

PPM1H

NEUROD6

BNC1

GABRR3

LRRC41

ATG9B

ZNF544

ZNF804A

NOBOX

TMEM47

GFOD1

FBXO33

CSN1S1

DMRT3

CHRFAM7A

ZNF385D

TCERG1L

SKOR1

SEM1

NCKAP5

RNF122

MIRLET7D

LOC108663987

HCNR606

HCNR617

HHC2:066650

GRIA3

GPHN

RAB11A

MFN2

BTK

MTOR

STAT1

MAPK10

HSPB1

HDAC2

CTSD

CASP8

CFTR

TLR2

DNMT3B

GRIA2

GRM1

MAPK8

GCK

CASR

SOD2

LEPR

HDAC1

IFNGR1

ESR2

TF

TGFB2

PIK3C3

SIRT1

SLC2A2

MYH9

PLAT

MYD88

NOS2

APOA1

ASS1

ITPR1

CYP1B1

CASP6

CCL2

MDH2

SQSTM1

MMP7

RUNX1

ODC1

RAB7A

RB1

C3

MAP3K5

HSPD1

KCNJ1

MAX

IFNG

FGF2

CYP1A1

CYP27B1

ABCC2

ALDH7A1

CASP9

TLR7

TLR5

TRPC3

YWHAH

TLR1

TGM2

SSTR2

STAT2

PPIA

SLC11A1

SLC12A2

SLC12A1

PDX1

PFN1

LIMK2

YWHAZ

APAF1

ABCC8

FOLH1

FBN1

IRF3

GRM8

GLP1R

FEN1

DDX58

CFI

AQP1

AQP4

CLU

ABCC3

ASL

BECN1

NCAM1

TRPM6

WNK1

PDE6B

PRDX6

SLC25A12

OGG1

PDE10A

NR1I2

PIK3R4

RAB5A

CTSL

IL1R1

HNF1A

CFH

APOB

ELOVL4

EIF2AK2

UCP2

TXN

TP73

PON1

KCNA5

FZD9

CTPS1

CYP51A1

CCR6

CD274

CD14

IL23R

LARS2

HAAO

HP

LAMA3

IL13

GIPR

CPE

AMD1

ADD1

BTC

CST3

PTPRN2

ULK1

TUBB2B

TLR9

TFAM

SP1

STK24

NR1I3

RPSA

REST

KMO

PTPRN

SLC34A1

APOH

ATP12A

FOXO3

DNAJB2

C4A

IL12B

HLA-DPB1

KALRN

CD34

DNAJB1

ABCB7

ENPP2

SPI1

TLR6

WNK4

SPINK1

STK39

SLC25A15

SLCO1B3

SLC4A2

NSD1

MLX

PAX4

RAB3A

RPS27A

PPIF

NEUROD1

FXYD2

APOL1

KLHL3

LAMP1

HSD3B7

LTA

HLA-DQA1

DFFB

DYNLL1

CHGA

CLDN16

CALB1

CALCA

ATG7

CRHR2

MNX1

VAC14

TREM2

POLI

PTPN4

SLC13A3

SETDB1

SH3GL3

MX1

RB1CC1

ATP4A

IAPP

IFNB1

MAFA

LIPF

JPH3

CFHR1

CLDN4

EIF4H

SRM

SSTR5

FLVCR1

CPLX2

BSND

ICA1

HLA-DPA1

FKBP10

CLDN11

CLCNKA

CLDN3

AAK1

TCERG1

TBPL1

MNDA

RHD

RUNX1T1

SMARCA5

PDLIM1

PCSK2

RPS15

RCOR1

SHBG

HAP1

LAT2

HPR

HPX

KCNJ16

NIF3L1

GAST

FKBP6

GPBAR1

AIF1

AADAT

ADAMTS12

SPG11

WNK3

YBX3

TAF4

SLCO1A2

SLC15A2

PVALB

DHX58

AMBRA1

WIPI1

SLC25A37

RASD2

ATG13

INSM1

UCN

ZDHHC17

MXD1

NSUN6

NSUN5

SCGN

SIGLEC5

ZDHHC13

ABHD11

BCL7B

ATG14

LARS1

CFHR2

CH25H

OMP

PLCXD3

PSG3

NSUN3

MXD4

CHERP

ATG101

MARS1

ERP29

ANKMY1

TUT1

IL31

INSM2

SCT

IFNA4

LMLN

ATP5F1B

CALHM1

DNAJC30

ZFP3

SLC25A2

NSUN7

MT-CO1

GTF2IRD2

FAM71A

MT-ND5

MT-ND6

ZNF395

TRIM50

GALNT17

BUD23

VPS37D

GTF2IRD2B

CRPPA

TRIM74

MEG3

POM121C

ANGPTL8

TRIM73

SYNDIG1L

SPDYE1

METTL27

INTS6L

CBARP

RCC1L

PRNT

NEAT1

TUG1

TMEM270

STAG3L1

NSUN5P2

MIR29A

STAG3L4

MIR204

MIR22

DGCR5

STAG3L2

MIR9-1

MIR148A

MIR330

HAR1A

BDNF-AS

NSUN5P1

HAR1B

SPDYE7P

STAG3L3

HTT-AS

ABHD11-AS1

TUNAR

LINC00851

GTF2IP1

GTF2IRD2P1

MMP2-AS1

GTF2IP4

SPDYE14

SPDYE8

SPDYE15

SPDYE12P

SPDYE10P

SPDYE13

EIF4HP1

SPDYE9

WBSCR2

WBSCR23

LOC109029536

LOC106029312

LOC108228208

LOC108228209

LOC106029311

LOC106029313

CYP2A6

CCKAR

PER1

AHR

ADRA1B

AHRR

FGFR1

CTNNB1

FGFR2

BRAF

PPP3CA

RET

PIK3CA

SLC2A1

RPS6KA3

HRAS

HDAC4

TERT

DNMT1

FLT1

DYRK1A

SPR

PTCH1

NF1

FBP1

CSNK2B

AIFM1

ACAT1

CAMK2B

TSC2

TBK1

TRPV4

SMO

PDGFB

SHH

GABBR2

IDS

GSN

GLRB

DMPK

CASK

USP8

USP7

TPO

STXBP1

PYGL

NF2

IRF5

HDAC8

GJB1

GLI2

GNAI3

GBA

FGF8

TSHR

TRAF3

TSC1

ZEB2

SOX2

PLCB4

KIF11

PTS

COL3A1

CTSH

DPYS

MAN1B1

HMGCL

HNRNPK

GLRA1

DDX6

EEF1A2

CLTC

DHCR7

BAP1

BMP2

EIF4G1

CTCF

TWIST1

TGIF1

ST3GAL3

SMC1A

POMT1

SLC22A5

SLC25A1

SET

SLC6A5

PHKG2

MUSK

OTX2

NEFH

MYOD1

PRPS1

SIK1

SIN3A

DLL1

EHMT1

EZR

ERCC4

VAMP1

SMARCB1

PNKP

PPT1

SMC3

SLC5A5

SMARCE1

PHKA2

RAD21

NDST1

KCNA1

CORIN

AGA

ASPA

IL12RB1

HDC

HERC2

NKX2-5

FBXW11

DYNC1H1

DKK1

AIP

ASCL1

CDH15

CDON

CACNG2

AGRN

TRIO

P2RY11

STAG2

TAOK1

SYNGAP1

TICAM1

TNFSF15

SOX3

POLR2A

PRMT7

SKI

SGSH

NONO

NSDHL

SCN1B

NGLY1

SNRPN

TNIK

FOXG1

GNE

HIBCH

FXR1

IL12A

KDM5B

GNS

CDKL5

CRADD

DCPS

CUX1

ARNT2

DUOX2

SPOP

VPS35

TRHR

ZIC2

SYT2

TECR

SUFU

POMT2

POU1F1

SLC18A3

SLC26A4

SLC5A7

SEPSECS

PIGQ

KCNQ5

LIAS

KAT6A

FOXE1

FOXH1

DOCK8

ADNP

ATXN10

IQSEC1

KIRREL3

MED23

KIF1A

IYD

NFIX

GATAD2B

FBXO7

FLII

GLUD2

GAS1

ERCC8

BRSK2

CRBN

AIMP1

ACTL6B

ADH1C

CDH23

CIC

EPM2A

EPB41L1

TUSC3

NDP

NAGLU

PUF60

TRPM3

TRMT1

TG

PSMD12

SARDH

NSUN2

MTRR

NODAL

SIX3

NDN

NEUROD2

NAA10

RAPSN

FLCN

AFF4

LARP7

LTBP3

CISD2

DNA2

ACTL6A

ARCN1

CEP57

TDGF1

TRAF7

ZMYND11

TDP2

PROKR2

RERE

LHX4

FKRP

COL13A1

COQ2

B3GALNT2

MBOAT7

GNPTAB

CNOT1

ASH1L

ATAD1

CHD8

NUP88

SPIB

NACC1

WDFY3

WDR45

TSEN2

TSPYL1

ZC3H14

ZMIZ1

TNFSF4

TRAPPC4

PIGC

POU3F3

PROP1

PRSS12

PGAP1

POGZ

PCGF2

NTNG1

NTNG2

SETD1A

SH3BP2

NHLRC1

NIPBL

LHX3

SIM1

ATP10A

FKTN

COLQ

DOK7

ATAD3A

MED25

HESX1

LRRC32

GIGYF2

FMN2

BPTF

CC2D1A

C2CD3

EML1

TSEN15

MKRN3

UNC93B1

WAC

TELO2

SRPX2

SLC35C1

SLITRK1

RLIM

SHANK3

PIGT

RSRC1

NALCN

KMT2E

RAI1

TNPO3

DISP1

MYO9A

WDR4

TSEN34

TRAPPC9

STOX1

PIGP

GPR101

CHAMP1

IQSEC2

HGSNAT

LMAN2L

HIVEP2

DNAJC13

TRIM8

NADK2

TSEN54

TBC1D24

PGAP3

POU2AF1

SETD5

MFSD8

MBD5

FBXO31

EPG5

MMEL1

VPS51

WDR73

KIF15

DUOXA2

CCDC47

DMXL2

TTI2

P4HTM

SEPTIN9

RTTN

SARS1

BCORL1

METTL23

FRRS1L

CARS1

CERT1

TWNK

SHROOM4

LARGE1

UNC80

SLC45A1

LINS1

SPART

RIC1

WASHC4

SVBP

NPAP1

COA8

ATXN8OS

PWRN1

IPW

SNORD115-1

PWAR1

SNORD116-1

MKRN3-AS1

ADRA1D

ARHGEF9

ADK

ADA

NT5E

BRCA1

AHCY

BRCA2

BAX

GABRB2

CYP2C19

PNLIP

SLC29A1

GABRA5

HMGCR

CYP1A2

GABRA3

SLC29A2

MB

SLC29A3

GPR50

SLC29A4

SLC28A1

SLC28A2

BCL2A1

THAP1

NT5C1B

ERBB2

CCND1

CDKN2A

TNFRSF10B

NFE2L2

EDNRA

EDNRB

PARP1

UGT1A1

MEF2A

KAT2B

GNB3

ATP7B

CAMK2G

TNFAIP3

KLK3

HSP90B1

F2RL1

ALPP

PTGS1

MDH1

GABRD

CRHR1

GABRA6

ADM

GAL

HRH2

GDI1

F3

AKR1A1

UCP1

PTGDS

CHI3L1

MBTPS2

MBTPS1

ALG1

TRIB3

ATP13A2

GNRH1

ADCYAP1

SULT1A1

KRIT1

ALDH9A1

COG2

DIO2

PPP1R15A

APBB2

AGFG1

TSC22D3

SLPI

PLEK

NES

TIMELESS

SLC36A1

IL1RAPL2

CELA3B

ADPRH

LEO1

EHD3

RBFOX3

TAS2R38

FREM3

PRIP

KURU

CJD
