# Supplementary material for: Exploration of Ziziphi Spinosae Semen in Treating Insomnia Based on Network Pharmacology Strategy
Source: Evid Based Complement Alternat Med. 2021 Oct 28;2021:9888607. doi: 10.1155/2021/9888607 (PMC8568550; doi:10.1155/2021/9888607)
Supplement: Supplementary Materials — Table S1: detailed information of related targets and gene symbols of compounds in ZSS. Table S2: the known therapeutic gene targets for insomnia. Table S3: detailed information about the D-C-G-D network. Table S4: overlapping gene symbols and corresponding degree values between disease and drug. Table S5: details of GO enrichment analyses. Table S6: details of KEGG pathway enrichment analyses. [file 9888607.f1.zip › 9888607.f1/Supplementary Table S3-Detailed information about D-C-G-D network (1).doc]

| **Detailed information about D-C-G-D network** | | | |
| --- | --- | --- | --- |
| **Drug** | **compounds** | **Targets** | **Disease** |
| Ziziphi Spinosae Semen | Ceanothic acid | NR1H4\ACE\OPRM1\ESR2\SHBG\CYP51A1\GPBAR1 | Insomnia |
|  | (S)-Coclaurine | DRD2\ADRB2\ADRB1\DRD1\DRD3\SLC6A3\ADRA1D\ADRA1A\DRD4\HTR1A\DRD5\ADRA1B\F3\HTR2A\MAOA\OPRK1\SLC6A4\HCRTR2\HCRTR1\ACHE\DHCR7\SLC6A2\CHRNA4\HTR2C\BCHE\ADRA2A\ADRA2C\ABCB1\SLC18A2\JAK2\GRIK2\HDAC1\ADRA2B\MTNR1B |  |
|  | Daucosterol | IL2\PSEN1\PSEN2\FLT1 |  |
|  | Jujubogenin | PDE10A\REN\MMP2\MMP7\NOS2\PTGS1\FAAH\MAPK8 |  |
|  | Phytosterol | HMGCR\CYP19A1\CYP17A1\ESR1\CYP2C19\VDR\GLRA1\PPARD\PTPN6\NR1I3\FDFT1\NR3C1\PPARG |  |
|  | Sanjoinenine | SHH\HTR1A\ADRA1D\ADRA1A\ADRA1B\OPRM1\JAK2\TACR3\SLC6A4\NOS2\TBK1\MAPK8\TACR1\SMO\NR1H4\HCRTR2\HCRTR1\HLA-DRB1\CHRM1\TERT\PDE10A\TSPO\ADRA2A\HTR6\KIF11\PDE2A\PLAT\KCNJ6\OPRK1\HDAC3\HRH1\PPIA\DRD1\KCNA5\F2RL1\CYP19A1 |  |
|  | Swertisin | TNF\IL2 |  |
|  | Zizyphusine | CHRNA4\DRD2\HTR1A\DRD3\HTR6\DRD1\ADRA1A\HTR2A\HTR5A\OPRM1\ADRA1D\DRD5\HTR1B\DRD4\ADRA1B\HTR2C\ADRA2A\ADRA2C\HTR1D\SLC6A4\HRH2\ADRA2B\ADRB1\TH\TSPO\SLC6A3\PSEN1\PSEN2\ERN1\GSK3B\ABCB1\ADRB2\KDR\NOS2\MMP2\ADORA1\ADORA2A\PIK3CA\PIK3CG\BACE1\HDAC8\HDAC4\MMP7\HDAC9 |  |
|  | Mairin | NR1H4\GPBAR1\VDR\PPARG\SHBG\CYP51A1\CYP19A1\TERT\CYP17A1\GABRG2\GABRB2\GABRA2\TLR9\PTPN6 |  |
|  | Jujuboside A | VEGFA\FGF2\IL2 |  |
|  | Spinosin | TNF\IL2 |  |
