# Supplementary material for: Exploration of Ziziphi Spinosae Semen in Treating Insomnia Based on Network Pharmacology Strategy
Source: Evid Based Complement Alternat Med. 2021 Oct 28;2021:9888607. doi: 10.1155/2021/9888607 (PMC8568550; doi:10.1155/2021/9888607)
Supplement: Supplementary Materials — Table S1: detailed information of related targets and gene symbols of compounds in ZSS. Table S2: the known therapeutic gene targets for insomnia. Table S3: detailed information about the D-C-G-D network. Table S4: overlapping gene symbols and corresponding degree values between disease and drug. Table S5: details of GO enrichment analyses. Table S6: details of KEGG pathway enrichment analyses. [file 9888607.f1.zip › 9888607.f1/Supplementary Table S4-Overlapping gene symbols and corresponding degree values between disease and drug (1).doc]

| **Overlapping gene symbols and corresponding degree values between disease and drug** | |
| --- | --- |
| **gene symbols** | **degree** |
| TH | 35 |
| SLC6A4 | 34 |
| DRD2 | 33 |
| MAOA | 32 |
| VEGFA | 32 |
| ACHE | 31 |
| CHRNA4 | 31 |
| OPRM1 | 30 |
| ESR1 | 29 |
| HTR1A | 29 |
| REN | 29 |
| ACE | 28 |
| PIK3CA | 27 |
| DRD4 | 26 |
| HTR2A | 26 |
| ADRA1B | 25 |
| ADRA2A | 25 |
| SLC6A2 | 25 |
| ADRA2C | 24 |
| DRD1 | 24 |
| MAPK8 | 24 |
| SLC18A2 | 24 |
| SLC6A3 | 24 |
| ADRA1A | 23 |
| ADRA1D | 23 |
| ADRB2 | 23 |
| DRD3 | 23 |
| ADRA2B | 22 |
| FGF2 | 22 |
| HTR5A | 22 |
| OPRK1 | 22 |
| CHRM1 | 21 |
| BCHE | 20 |
| DRD5 | 20 |
| HTR2C | 20 |
| NR3C1 | 20 |
| HTR1B | 19 |
| IL2 | 19 |
| PPARG | 19 |
| ADORA1 | 18 |
| ADRB1 | 18 |
| KDR | 18 |
| JAK2 | 17 |
| MMP2 | 17 |
| CYP19A1 | 16 |
| HDAC1 | 16 |
| HTR1D | 16 |
| GSK3B | 14 |
| MTNR1B | 14 |
| TACR1 | 14 |
| F2RL1 | 13 |
| HCRTR1 | 13 |
| ABCB1 | 12 |
| GABRB2 | 12 |
| HCRTR2 | 12 |
| HRH1 | 12 |
| SHH | 12 |
| TACR3 | 12 |
| F3 | 11 |
| GLRA1 | 11 |
| NOS2 | 11 |
| BACE1 | 10 |
| GABRA2 | 10 |
| VDR | 10 |
| CYP2C19 | 9 |
| FLT1 | 9 |
| GABRG2 | 9 |
| GPBAR1 | 9 |
| HMGCR | 9 |
| KCNJ6 | 9 |
| PSEN1 | 9 |
| CYP17A1 | 8 |
| ESR2 | 8 |
| HDAC3 | 8 |
| HDAC4 | 8 |
| PLAT | 8 |
| TLR9 | 8 |
| ADORA2A | 7 |
| HTR6 | 7 |
| MMP7 | 7 |
| TERT | 7 |
| DHCR7 | 6 |
| HDAC9 | 6 |
| HRH2 | 6 |
| NR1H4 | 6 |
| PSEN2 | 6 |
| PTPN6 | 6 |
| CYP51A1 | 5 |
| FAAH | 5 |
| HLA-DRB1 | 5 |
| PTGS1 | 5 |
| HDAC8 | 4 |
| PIK3CG | 4 |
| PPIA | 4 |
| SHBG | 4 |
| TBK1 | 4 |
| FDFT1 | 3 |
| GRIK2 | 3 |
| NR1I3 | 3 |
| TSPO | 3 |
| ERN1 | 2 |
| PDE10A | 2 |
| PPARD | 2 |
| KIF11 | 1 |
| SMO | 1 |
