# Supplementary material for: Exploration of Ziziphi Spinosae Semen in Treating Insomnia Based on Network Pharmacology Strategy
Source: Evid Based Complement Alternat Med. 2021 Oct 28;2021:9888607. doi: 10.1155/2021/9888607 (PMC8568550; doi:10.1155/2021/9888607)
Supplement: Supplementary Materials — Table S1: detailed information of related targets and gene symbols of compounds in ZSS. Table S2: the known therapeutic gene targets for insomnia. Table S3: detailed information about the D-C-G-D network. Table S4: overlapping gene symbols and corresponding degree values between disease and drug. Table S5: details of GO enrichment analyses. Table S6: details of KEGG pathway enrichment analyses. [file 9888607.f1.zip › 9888607.f1/Supplementary Table S5-Details of GO enrichment analyses (1).doc]

| **Details of GO enrichment analyses** | | | | | | |
| --- | --- | --- | --- | --- | --- | --- |
| **ONTOLOGY** | **ID** | **Description** | **pvalue** | **p.adjust** | **geneID** | **Count** |
| BP | GO:0007187 | G protein-coupled receptor signaling pathway, coupled to cyclic nucleotide second messenger | 1.19E-30 | 2.8E-27 | DRD2/OPRM1/HTR1A/DRD4/HTR2A/ADRA1B/ADRA2A/ADRA2C/DRD1/ADRA1A/ADRA1D/ADRB2/DRD3/ADRA2B/HTR5A/OPRK1/CHRM1/DRD5/HTR2C/HTR1B/ADORA1/ADRB1/HTR1D | 23 |
| BP | GO:0007188 | adenylate cyclase-modulating G protein-coupled receptor signaling pathway | 2.21E-28 | 2.59E-25 | DRD2/OPRM1/HTR1A/DRD4/ADRA1B/ADRA2A/ADRA2C/DRD1/ADRA1A/ADRA1D/ADRB2/DRD3/ADRA2B/HTR5A/OPRK1/CHRM1/DRD5/HTR1B/ADORA1/ADRB1/HTR1D | 21 |
| BP | GO:0015844 | monoamine transport | 4.15E-26 | 3.24E-23 | SLC6A4/DRD2/CHRNA4/HTR1A/DRD4/HTR2A/ADRA2A/SLC6A2/ADRA2C/DRD1/SLC18A2/SLC6A3/DRD3/ADRA2B/OPRK1/HTR1B | 16 |
| BP | GO:0015850 | organic hydroxy compound transport | 2.6E-23 | 1.52E-20 | SLC6A4/DRD2/CHRNA4/HTR1A/REN/DRD4/HTR2A/ADRA2A/SLC6A2/ADRA2C/DRD1/SLC18A2/SLC6A3/DRD3/ADRA2B/OPRK1/HTR1B/PPARG/CYP19A1 | 19 |
| BP | GO:0003018 | vascular process in circulatory system | 4.97E-23 | 1.99E-20 | SLC6A4/VEGFA/HTR1A/ACE/HTR2A/ADRA1B/ADRA2A/ADRA2C/DRD1/ADRA1A/ADRB2/ADRA2B/DRD5/HTR1B/ADORA1/ADRB1/HTR1D | 17 |
| BP | GO:0051937 | catecholamine transport | 5.1E-23 | 1.99E-20 | DRD2/CHRNA4/DRD4/HTR2A/ADRA2A/SLC6A2/ADRA2C/DRD1/SLC18A2/SLC6A3/DRD3/ADRA2B/OPRK1/HTR1B | 14 |
| BP | GO:0035296 | regulation of tube diameter | 1.08E-22 | 3.17E-20 | SLC6A4/HTR1A/ACE/HTR2A/ADRA1B/ADRA2A/ADRA2C/DRD1/ADRA1A/ADRB2/ADRA2B/DRD5/HTR1B/ADORA1/ADRB1/HTR1D | 16 |
| BP | GO:0097746 | regulation of blood vessel diameter | 1.08E-22 | 3.17E-20 | SLC6A4/HTR1A/ACE/HTR2A/ADRA1B/ADRA2A/ADRA2C/DRD1/ADRA1A/ADRB2/ADRA2B/DRD5/HTR1B/ADORA1/ADRB1/HTR1D | 16 |
| BP | GO:0035150 | regulation of tube size | 1.22E-22 | 3.17E-20 | SLC6A4/HTR1A/ACE/HTR2A/ADRA1B/ADRA2A/ADRA2C/DRD1/ADRA1A/ADRB2/ADRA2B/DRD5/HTR1B/ADORA1/ADRB1/HTR1D | 16 |
| BP | GO:0015837 | amine transport | 2.58E-21 | 6.04E-19 | TH/DRD2/CHRNA4/DRD4/HTR2A/ADRA2A/ADRA2C/DRD1/SLC18A2/DRD3/ADRA2B/OPRK1/HTR1B/ADORA1 | 14 |
| BP | GO:0001505 | regulation of neurotransmitter levels | 8.68E-21 | 1.85E-18 | TNF/TH/SLC6A4/DRD2/MAOA/ACHE/CHRNA4/HTR1A/DRD4/HTR2A/SLC6A2/DRD1/SLC18A2/SLC6A3/ADRA1A/DRD3/BCHE/HTR1B/JAK2 | 19 |
| BP | GO:0097756 | negative regulation of blood vessel diameter | 1.99E-20 | 3.89E-18 | SLC6A4/HTR1A/ACE/HTR2A/ADRA1B/ADRA2A/ADRA2C/ADRA1A/ADRA2B/DRD5/HTR1B/ADORA1/HTR1D | 13 |
| BP | GO:1903351 | cellular response to dopamine | 8.13E-20 | 1.47E-17 | DRD2/OPRM1/HTR1A/DRD4/HTR2A/DRD1/DRD3/HTR5A/CHRM1/DRD5/HTR2C/HTR1B/HTR1D | 13 |
| BP | GO:1903350 | response to dopamine | 9.42E-20 | 1.58E-17 | DRD2/OPRM1/HTR1A/DRD4/HTR2A/DRD1/DRD3/HTR5A/CHRM1/DRD5/HTR2C/HTR1B/HTR1D | 13 |
| BP | GO:0071880 | adenylate cyclase-activating adrenergic receptor signaling pathway | 1.97E-19 | 3.08E-17 | DRD2/ADRA1B/ADRA2A/ADRA2C/ADRA1A/ADRA1D/ADRB2/DRD3/ADRA2B/ADRB1 | 10 |
| BP | GO:0035690 | cellular response to drug | 3.68E-19 | 5.38E-17 | TNF/TH/DRD2/CHRNA4/OPRM1/HTR1A/REN/DRD4/HTR2A/DRD1/DRD3/HTR5A/CHRM1/DRD5/HTR2C/NR3C1/HTR1B/KDR/HTR1D | 19 |
| BP | GO:0042310 | vasoconstriction | 5.17E-19 | 6.87E-17 | SLC6A4/HTR1A/ACE/HTR2A/ADRA1B/ADRA2A/ADRA2C/ADRA1A/ADRA2B/DRD5/HTR1B/HTR1D | 12 |
| BP | GO:0015872 | dopamine transport | 5.28E-19 | 6.87E-17 | DRD2/CHRNA4/DRD4/HTR2A/SLC6A2/DRD1/SLC18A2/SLC6A3/DRD3/OPRK1/HTR1B | 11 |
| BP | GO:0071875 | adrenergic receptor signaling pathway | 5.8E-19 | 7.15E-17 | DRD2/ADRA1B/ADRA2A/ADRA2C/ADRA1A/ADRA1D/ADRB2/DRD3/ADRA2B/ADRB1 | 10 |
| BP | GO:0071868 | cellular response to monoamine stimulus | 7.12E-19 | 7.95E-17 | DRD2/OPRM1/HTR1A/DRD4/HTR2A/DRD1/DRD3/HTR5A/CHRM1/DRD5/HTR2C/HTR1B/HTR1D | 13 |
| BP | GO:0071870 | cellular response to catecholamine stimulus | 7.12E-19 | 7.95E-17 | DRD2/OPRM1/HTR1A/DRD4/HTR2A/DRD1/DRD3/HTR5A/CHRM1/DRD5/HTR2C/HTR1B/HTR1D | 13 |
| BP | GO:0043279 | response to alkaloid | 1.03E-18 | 1.1E-16 | TH/DRD2/OPRM1/DRD4/HTR2A/DRD1/SLC6A3/DRD3/OPRK1/BCHE/DRD5/HTR1B/PPARG | 13 |
| BP | GO:0071867 | response to monoamine | 1.16E-18 | 1.13E-16 | DRD2/OPRM1/HTR1A/DRD4/HTR2A/DRD1/DRD3/HTR5A/CHRM1/DRD5/HTR2C/HTR1B/HTR1D | 13 |
| BP | GO:0071869 | response to catecholamine | 1.16E-18 | 1.13E-16 | DRD2/OPRM1/HTR1A/DRD4/HTR2A/DRD1/DRD3/HTR5A/CHRM1/DRD5/HTR2C/HTR1B/HTR1D | 13 |
| BP | GO:0019935 | cyclic-nucleotide-mediated signaling | 3.21E-18 | 3.01E-16 | DRD2/OPRM1/ADRA1B/ADRA2A/ADRA2C/DRD1/ADRA1A/ADRA1D/ADRB2/DRD3/ADRA2B/HTR5A/DRD5/HTR2C/ADRB1 | 15 |
| BP | GO:0007193 | adenylate cyclase-inhibiting G protein-coupled receptor signaling pathway | 3.86E-18 | 3.48E-16 | DRD2/OPRM1/HTR1A/DRD4/ADRA2A/DRD3/HTR5A/OPRK1/CHRM1/HTR1B/ADORA1/HTR1D | 12 |
| BP | GO:0051952 | regulation of amine transport | 6.73E-18 | 5.84E-16 | DRD2/CHRNA4/DRD4/HTR2A/ADRA2A/ADRA2C/DRD1/DRD3/ADRA2B/OPRK1/HTR1B/ADORA1 | 12 |
| BP | GO:0019932 | second-messenger-mediated signaling | 1.31E-17 | 1.09E-15 | TNF/DRD2/VEGFA/OPRM1/ADRA1B/ADRA2A/ADRA2C/DRD1/ADRA1A/ADRA1D/ADRB2/DRD3/ADRA2B/HTR5A/DRD5/HTR2C/ADRB1/KDR | 18 |
| BP | GO:0007200 | phospholipase C-activating G protein-coupled receptor signaling pathway | 1.68E-17 | 1.35E-15 | DRD2/OPRM1/ESR1/HTR2A/ADRA1B/ADRA2A/DRD1/ADRA1A/OPRK1/CHRM1/DRD5/HTR2C | 12 |
| BP | GO:0019933 | cAMP-mediated signaling | 1.78E-17 | 1.39E-15 | DRD2/OPRM1/ADRA1B/ADRA2A/ADRA2C/DRD1/ADRA1A/ADRA1D/ADRB2/DRD3/ADRA2B/HTR5A/DRD5/ADRB1 | 14 |
| BP | GO:0007189 | adenylate cyclase-activating G protein-coupled receptor signaling pathway | 1.88E-17 | 1.42E-15 | DRD2/OPRM1/ADRA1B/ADRA2A/ADRA2C/DRD1/ADRA1A/ADRA1D/ADRB2/DRD3/ADRA2B/DRD5/ADRB1 | 13 |
| BP | GO:0006836 | neurotransmitter transport | 1.22E-16 | 8.83E-15 | TH/SLC6A4/DRD2/CHRNA4/HTR1A/DRD4/HTR2A/SLC6A2/DRD1/SLC18A2/SLC6A3/ADRA1A/DRD3/HTR2C/HTR1B | 15 |
| BP | GO:0042220 | response to cocaine | 1.24E-16 | 8.83E-15 | DRD2/OPRM1/DRD4/HTR2A/DRD1/SLC6A3/DRD3/OPRK1/DRD5/HTR1B | 10 |
| BP | GO:0009410 | response to xenobiotic stimulus | 3.94E-16 | 2.71E-14 | TH/DRD2/OPRM1/DRD4/HTR2A/DRD1/SLC18A2/SLC6A3/DRD3/OPRK1/BCHE/DRD5/NR3C1/HTR1B/PPARG | 15 |
| BP | GO:0006939 | smooth muscle contraction | 3.35E-15 | 2.24E-13 | DRD2/HTR2A/ADRA1B/ADRA2A/ADRA2C/DRD1/ADRA1A/ADRB2/ADRA2B/ADORA1/HTR1D | 11 |
| BP | GO:0072347 | response to anesthetic | 5.13E-15 | 3.34E-13 | DRD2/OPRM1/DRD4/HTR2A/DRD1/SLC6A3/DRD3/OPRK1/DRD5/HTR1B | 10 |
| BP | GO:0050804 | modulation of chemical synaptic transmission | 6.77E-15 | 4.29E-13 | TNF/SLC6A4/DRD2/ACHE/CHRNA4/DRD4/HTR2A/DRD1/ADRA1A/ADRB2/DRD3/BCHE/DRD5/HTR1B/ADORA1/JAK2 | 16 |
| BP | GO:0099177 | regulation of trans-synaptic signaling | 7.02E-15 | 4.33E-13 | TNF/SLC6A4/DRD2/ACHE/CHRNA4/DRD4/HTR2A/DRD1/ADRA1A/ADRB2/DRD3/BCHE/DRD5/HTR1B/ADORA1/JAK2 | 16 |
| BP | GO:0001963 | synaptic transmission, dopaminergic | 7.79E-15 | 4.68E-13 | TH/SLC6A4/DRD2/DRD4/SLC6A2/DRD1/DRD3/DRD5 | 8 |
| BP | GO:0098664 | G protein-coupled serotonin receptor signaling pathway | 1.78E-14 | 1.04E-12 | HTR1A/DRD4/HTR2A/HTR5A/CHRM1/HTR2C/HTR1B/HTR1D | 8 |
| BP | GO:0008217 | regulation of blood pressure | 2.62E-14 | 1.5E-12 | DRD2/REN/ACE/ADRA1B/ADRA1A/ADRB2/DRD3/ADRA2B/DRD5/PPARG/ADORA1/ADRB1 | 12 |
| BP | GO:0003073 | regulation of systemic arterial blood pressure | 3.2E-14 | 1.79E-12 | DRD2/REN/ACE/ADRA1B/ADRA1A/ADRB2/DRD3/DRD5/ADORA1/ADRB1 | 10 |
| BP | GO:0050433 | regulation of catecholamine secretion | 4.06E-14 | 2.21E-12 | DRD2/CHRNA4/HTR2A/ADRA2A/ADRA2C/DRD3/ADRA2B/OPRK1/HTR1B | 9 |
| BP | GO:0051588 | regulation of neurotransmitter transport | 4.66E-14 | 2.48E-12 | SLC6A4/DRD2/CHRNA4/HTR1A/DRD4/HTR2A/DRD1/ADRA1A/DRD3/HTR2C/HTR1B | 11 |
| BP | GO:0050432 | catecholamine secretion | 5.49E-14 | 2.86E-12 | DRD2/CHRNA4/HTR2A/ADRA2A/ADRA2C/DRD3/ADRA2B/OPRK1/HTR1B | 9 |
| BP | GO:0007210 | serotonin receptor signaling pathway | 5.99E-14 | 2.98E-12 | HTR1A/DRD4/HTR2A/HTR5A/CHRM1/HTR2C/HTR1B/HTR1D | 8 |
| BP | GO:0042417 | dopamine metabolic process | 5.99E-14 | 2.98E-12 | TH/DRD2/MAOA/HTR1A/DRD4/DRD1/SLC6A3/DRD3 | 8 |
| BP | GO:0050805 | negative regulation of synaptic transmission | 1.67E-13 | 8.16E-12 | SLC6A4/DRD2/ACHE/HTR2A/DRD1/BCHE/DRD5/HTR1B/ADORA1 | 9 |
| BP | GO:0001504 | neurotransmitter uptake | 2.5E-13 | 1.2E-11 | SLC6A4/DRD2/DRD4/SLC6A2/DRD1/SLC18A2/SLC6A3/DRD3 | 8 |
| BP | GO:0006874 | cellular calcium ion homeostasis | 2.91E-13 | 1.36E-11 | DRD2/ESR1/DRD4/HTR2A/ADRA1B/DRD1/ADRA1A/DRD3/FGF2/DRD5/HTR2C/HTR1B/IL2/ADORA1/JAK2 | 15 |
| BP | GO:0050890 | cognition | 3.61E-13 | 1.66E-11 | TNF/TH/SLC6A4/DRD2/CHRNA4/HTR2A/DRD1/DRD3/OPRK1/CHRM1/BCHE/DRD5/ADORA1 | 13 |
| BP | GO:1903522 | regulation of blood circulation | 3.77E-13 | 1.7E-11 | TH/DRD2/ACE/HTR2A/ADRA1B/ADRA2A/ADRA2C/ADRA1A/ADRA2B/IL2/ADORA1/ADRB1/JAK2 | 13 |
| BP | GO:0098810 | neurotransmitter reuptake | 4.01E-13 | 1.77E-11 | SLC6A4/DRD2/DRD4/SLC6A2/DRD1/SLC18A2/DRD3 | 7 |
| BP | GO:0003044 | regulation of systemic arterial blood pressure mediated by a chemical signal | 4.3E-13 | 1.86E-11 | REN/ACE/ADRA1B/ADRA1A/ADRB2/DRD3/DRD5/ADRB1 | 8 |
| BP | GO:0055074 | calcium ion homeostasis | 4.36E-13 | 1.86E-11 | DRD2/ESR1/DRD4/HTR2A/ADRA1B/DRD1/ADRA1A/DRD3/FGF2/DRD5/HTR2C/HTR1B/IL2/ADORA1/JAK2 | 15 |
| BP | GO:0072503 | cellular divalent inorganic cation homeostasis | 8.42E-13 | 3.52E-11 | DRD2/ESR1/DRD4/HTR2A/ADRA1B/DRD1/ADRA1A/DRD3/FGF2/DRD5/HTR2C/HTR1B/IL2/ADORA1/JAK2 | 15 |
| BP | GO:0006584 | catecholamine metabolic process | 9.84E-13 | 3.97E-11 | TH/DRD2/MAOA/HTR1A/DRD4/DRD1/SLC6A3/DRD3 | 8 |
| BP | GO:0009712 | catechol-containing compound metabolic process | 9.84E-13 | 3.97E-11 | TH/DRD2/MAOA/HTR1A/DRD4/DRD1/SLC6A3/DRD3 | 8 |
| BP | GO:0090494 | dopamine uptake | 1.03E-12 | 4.08E-11 | DRD2/DRD4/SLC6A2/DRD1/SLC6A3/DRD3 | 6 |
| BP | GO:0001975 | response to amphetamine | 1.51E-12 | 5.89E-11 | TH/DRD2/DRD4/DRD1/SLC18A2/DRD3/DRD5 | 7 |
| BP | GO:0090493 | catecholamine uptake | 1.64E-12 | 6.3E-11 | DRD2/DRD4/SLC6A2/DRD1/SLC6A3/DRD3 | 6 |
| BP | GO:0007626 | locomotory behavior | 2.32E-12 | 8.76E-11 | TH/DRD2/CHRNA4/OPRM1/DRD4/DRD1/SLC18A2/SLC6A3/DRD3/OPRK1/HTR2C | 11 |
| BP | GO:0015893 | drug transport | 3.39E-12 | 1.26E-10 | DRD2/CHRNA4/DRD4/HTR2A/SLC6A2/DRD1/SLC18A2/SLC6A3/DRD3/OPRK1/HTR1B | 11 |
| BP | GO:0045777 | positive regulation of blood pressure | 4.57E-12 | 1.67E-10 | ACE/ADRA1B/ADRA1A/ADRA2B/DRD5/ADORA1/ADRB1 | 7 |
| BP | GO:1903531 | negative regulation of secretion by cell | 4.88E-12 | 1.76E-10 | TNF/DRD2/OPRM1/DRD4/ADRA2A/ADRA2C/DRD3/ADRA2B/OPRK1/HTR1B/ADORA1 | 11 |
| BP | GO:0048148 | behavioral response to cocaine | 7.88E-12 | 2.8E-10 | DRD2/DRD4/HTR2A/DRD1/DRD3/OPRK1 | 6 |
| BP | GO:0048511 | rhythmic process | 8.13E-12 | 2.84E-10 | TH/SLC6A4/DRD2/OPRM1/ESR1/DRD4/MAPK8/DRD3/OPRK1/PPARG/ADORA1/HDAC1 | 12 |
| BP | GO:0051048 | negative regulation of secretion | 1.71E-11 | 5.89E-10 | TNF/DRD2/OPRM1/DRD4/ADRA2A/ADRA2C/DRD3/ADRA2B/OPRK1/HTR1B/ADORA1 | 11 |
| BP | GO:0001659 | temperature homeostasis | 1.73E-11 | 5.89E-10 | TNF/DRD2/VEGFA/ACHE/HTR2A/DRD1/ADRB2/ADORA1/ADRB1/JAK2 | 10 |
| BP | GO:0014075 | response to amine | 2.33E-11 | 7.7E-10 | TH/DRD2/DRD4/DRD1/SLC18A2/DRD3/DRD5 | 7 |
| BP | GO:0045776 | negative regulation of blood pressure | 2.33E-11 | 7.7E-10 | DRD2/ADRA1A/ADRB2/DRD3/DRD5/ADORA1/ADRB1 | 7 |
| BP | GO:0032147 | activation of protein kinase activity | 3.33E-11 | 1.09E-09 | TNF/DRD2/VEGFA/PIK3CA/DRD4/ADRA2A/ADRA2C/ADRB2/ADRA2B/FGF2/ADORA1/JAK2 | 12 |
| BP | GO:0051583 | dopamine uptake involved in synaptic transmission | 4.14E-11 | 1.31E-09 | DRD2/DRD4/SLC6A2/DRD1/DRD3 | 5 |
| BP | GO:0051934 | catecholamine uptake involved in synaptic transmission | 4.14E-11 | 1.31E-09 | DRD2/DRD4/SLC6A2/DRD1/DRD3 | 5 |
| BP | GO:0046883 | regulation of hormone secretion | 4.81E-11 | 1.5E-09 | TNF/DRD2/HTR1A/REN/ADRA2A/ADRA2C/OPRK1/HTR2C/ADORA1/JAK2/CYP19A1 | 11 |
| BP | GO:0043112 | receptor metabolic process | 4.87E-11 | 1.5E-09 | TNF/DRD2/VEGFA/ACHE/DRD4/DRD3/HTR1B/PPARG/JAK2/HDAC1 | 10 |
| BP | GO:0097366 | response to bronchodilator | 5E-11 | 1.52E-09 | TH/DRD2/DRD4/DRD1/SLC18A2/DRD3/DRD5 | 7 |
| BP | GO:0051480 | regulation of cytosolic calcium ion concentration | 7.46E-11 | 2.24E-09 | DRD2/ESR1/HTR2A/ADRA1B/DRD1/ADRA1A/FGF2/HTR2C/HTR1B/IL2/ADORA1/JAK2 | 12 |
| BP | GO:0006936 | muscle contraction | 9.04E-11 | 2.68E-09 | DRD2/PIK3CA/HTR2A/ADRA1B/ADRA2A/ADRA2C/DRD1/ADRA1A/ADRB2/ADRA2B/ADORA1/HTR1D | 12 |
| BP | GO:0019229 | regulation of vasoconstriction | 1.28E-10 | 3.75E-09 | ACE/HTR2A/ADRA1B/ADRA2A/ADRA2C/ADRA1A/ADRA2B | 7 |
| BP | GO:0099003 | vesicle-mediated transport in synapse | 1.36E-10 | 3.92E-09 | TH/DRD2/CHRNA4/DRD4/HTR2A/DRD1/SLC18A2/ADRA1A/DRD3/HTR1B | 10 |
| BP | GO:0042133 | neurotransmitter metabolic process | 1.68E-10 | 4.8E-09 | TNF/TH/SLC6A4/MAOA/ACHE/HTR1A/SLC6A3/BCHE/JAK2 | 9 |
| BP | GO:0007631 | feeding behavior | 1.75E-10 | 4.93E-09 | TH/DRD2/OPRM1/REN/DRD1/OPRK1/HTR2C/HTR1B | 8 |
| BP | GO:0018958 | phenol-containing compound metabolic process | 2.05E-10 | 5.71E-09 | TH/DRD2/MAOA/HTR1A/DRD4/DRD1/SLC6A3/DRD3 | 8 |
| BP | GO:2001023 | regulation of response to drug | 2.58E-10 | 7.12E-09 | DRD2/CHRNA4/DRD4/HTR2A/DRD1/DRD3/OPRK1/HTR1B | 8 |
| BP | GO:0046879 | hormone secretion | 2.71E-10 | 7.37E-09 | TNF/DRD2/HTR1A/REN/ADRA2A/ADRA2C/OPRK1/HTR2C/ADORA1/JAK2/CYP19A1 | 11 |
| BP | GO:0006940 | regulation of smooth muscle contraction | 2.93E-10 | 7.88E-09 | ADRA1B/ADRA2A/ADRA2C/ADRA1A/ADRB2/ADRA2B/ADORA1 | 7 |
| BP | GO:0009914 | hormone transport | 3.67E-10 | 9.78E-09 | TNF/DRD2/HTR1A/REN/ADRA2A/ADRA2C/OPRK1/HTR2C/ADORA1/JAK2/CYP19A1 | 11 |
| BP | GO:0032225 | regulation of synaptic transmission, dopaminergic | 3.88E-10 | 1.02E-08 | SLC6A4/DRD2/DRD4/DRD1/DRD3 | 5 |
| BP | GO:0050795 | regulation of behavior | 5.52E-10 | 1.44E-08 | DRD2/HTR1A/DRD3/OPRK1/HTR1B/ADORA1/HTR1D | 7 |
| BP | GO:0014046 | dopamine secretion | 7.5E-10 | 1.91E-08 | DRD2/CHRNA4/HTR2A/DRD3/OPRK1/HTR1B | 6 |
| BP | GO:0014059 | regulation of dopamine secretion | 7.5E-10 | 1.91E-08 | DRD2/CHRNA4/HTR2A/DRD3/OPRK1/HTR1B | 6 |
| BP | GO:0045471 | response to ethanol | 9.71E-10 | 2.45E-08 | TH/DRD2/OPRM1/DRD4/SLC6A3/OPRK1/HTR1B/IL2 | 8 |
| BP | GO:0050731 | positive regulation of peptidyl-tyrosine phosphorylation | 1.27E-09 | 3.15E-08 | TNF/VEGFA/ACE/HTR2A/ADRA2A/ADRA1A/IL2/ADORA1/JAK2 | 9 |
| BP | GO:0007212 | dopamine receptor signaling pathway | 1.37E-09 | 3.38E-08 | DRD2/OPRM1/DRD4/DRD1/DRD3/DRD5 | 6 |
| BP | GO:0003012 | muscle system process | 1.81E-09 | 4.42E-08 | DRD2/PIK3CA/HTR2A/ADRA1B/ADRA2A/ADRA2C/DRD1/ADRA1A/ADRB2/ADRA2B/ADORA1/HTR1D | 12 |
| BP | GO:0060047 | heart contraction | 2.08E-09 | 5.02E-08 | TH/DRD2/ACE/PIK3CA/ADRA1B/ADRA1A/IL2/ADORA1/ADRB1/JAK2 | 10 |
| BP | GO:0060078 | regulation of postsynaptic membrane potential | 2.4E-09 | 5.73E-08 | DRD2/CHRNA4/OPRM1/DRD4/ADRB2/CHRM1/ADORA1/ADRB1 | 8 |
| BP | GO:0007623 | circadian rhythm | 2.56E-09 | 6.06E-08 | TH/SLC6A4/DRD2/DRD4/MAPK8/DRD3/PPARG/ADORA1/HDAC1 | 9 |
| BP | GO:0003015 | heart process | 2.91E-09 | 6.81E-08 | TH/DRD2/ACE/PIK3CA/ADRA1B/ADRA1A/IL2/ADORA1/ADRB1/JAK2 | 10 |
| BP | GO:0030534 | adult behavior | 2.99E-09 | 6.95E-08 | DRD2/CHRNA4/OPRM1/DRD4/HTR2A/DRD1/DRD3/OPRK1 | 8 |
| BP | GO:0002791 | regulation of peptide secretion | 3.46E-09 | 7.95E-08 | TNF/DRD2/ACHE/OPRM1/DRD4/ADRA2A/ADRA2C/DRD3/HTR2C/IL2/ADORA1/JAK2 | 12 |
| BP | GO:0051953 | negative regulation of amine transport | 4.63E-09 | 0.000000105 | DRD2/ADRA2A/ADRA2C/ADRA2B/ADORA1 | 5 |
| BP | GO:0032800 | receptor biosynthetic process | 5.73E-09 | 0.000000129 | TNF/ACHE/PPARG/JAK2/HDAC1 | 5 |
| BP | GO:0007204 | positive regulation of cytosolic calcium ion concentration | 6.78E-09 | 0.000000151 | DRD2/ESR1/HTR2A/ADRA1B/DRD1/ADRA1A/FGF2/HTR2C/IL2/JAK2 | 10 |
| BP | GO:0097305 | response to alcohol | 6.91E-09 | 0.000000153 | TH/DRD2/OPRM1/DRD4/SLC6A3/OPRK1/HTR1B/IL2/PPARG | 9 |
| BP | GO:0007191 | adenylate cyclase-activating dopamine receptor signaling pathway | 7.98E-09 | 0.000000175 | OPRM1/DRD1/DRD3/DRD5 | 4 |
| BP | GO:0042391 | regulation of membrane potential | 9.84E-09 | 0.000000213 | DRD2/CHRNA4/OPRM1/DRD4/DRD1/ADRA1A/ADRB2/CHRM1/ADORA1/ADRB1/KDR | 11 |
| BP | GO:0071902 | positive regulation of protein serine/threonine kinase activity | 1.05E-08 | 0.000000226 | TNF/VEGFA/DRD4/HTR2A/ADRA2A/ADRB2/ADRA2B/FGF2/ADORA1/JAK2 | 10 |
| BP | GO:0007611 | learning or memory | 1.57E-08 | 0.000000331 | TH/SLC6A4/DRD2/HTR2A/DRD1/DRD3/OPRK1/BCHE/DRD5 | 9 |
| BP | GO:0050730 | regulation of peptidyl-tyrosine phosphorylation | 1.57E-08 | 0.000000331 | TNF/VEGFA/ACE/HTR2A/ADRA2A/ADRA1A/IL2/ADORA1/JAK2 | 9 |
| BP | GO:0043406 | positive regulation of MAP kinase activity | 1.68E-08 | 0.00000035 | TNF/VEGFA/DRD4/HTR2A/ADRA2A/ADRA2B/FGF2/ADORA1/JAK2 | 9 |
| BP | GO:0040014 | regulation of multicellular organism growth | 1.69E-08 | 0.00000035 | DRD2/PIK3CA/SLC6A3/ADRB2/DRD3/ADRB1 | 6 |
| BP | GO:0018108 | peptidyl-tyrosine phosphorylation | 2.31E-08 | 0.000000475 | TNF/VEGFA/ACE/HTR2A/ADRA2A/ADRA1A/IL2/ADORA1/KDR/JAK2 | 10 |
| BP | GO:2000273 | positive regulation of signaling receptor activity | 2.39E-08 | 0.000000486 | ADRA2A/ADRA2C/ADRB2/ADRA2B/ADORA1 | 5 |
| BP | GO:0051966 | regulation of synaptic transmission, glutamatergic | 2.42E-08 | 0.000000489 | TNF/DRD2/HTR2A/DRD1/HTR1B/ADORA1 | 6 |
| BP | GO:0018212 | peptidyl-tyrosine modification | 0.000000025 | 0.000000501 | TNF/VEGFA/ACE/HTR2A/ADRA2A/ADRA1A/IL2/ADORA1/KDR/JAK2 | 10 |
| BP | GO:0098657 | import into cell | 2.75E-08 | 0.000000547 | SLC6A4/DRD2/DRD4/SLC6A2/DRD1/SLC18A2/SLC6A3/DRD3 | 8 |
| BP | GO:0043270 | positive regulation of ion transport | 2.91E-08 | 0.000000572 | SLC6A4/DRD2/DRD4/ADRA2A/DRD1/ADRB2/OPRK1/CHRM1/ADORA1 | 9 |
| BP | GO:0099504 | synaptic vesicle cycle | 2.99E-08 | 0.000000583 | TH/DRD2/CHRNA4/HTR2A/DRD1/SLC18A2/ADRA1A/HTR1B | 8 |
| BP | GO:0033555 | multicellular organismal response to stress | 3.12E-08 | 0.000000604 | HTR1A/DRD4/SLC6A2/DRD1/HTR2C/ADRB1 | 6 |
| BP | GO:0048871 | multicellular organismal homeostasis | 3.15E-08 | 0.000000604 | TNF/DRD2/VEGFA/ACHE/PIK3CA/HTR2A/DRD1/ADRB2/ADORA1/ADRB1/JAK2 | 11 |
| BP | GO:2000300 | regulation of synaptic vesicle exocytosis | 4.67E-08 | 0.000000889 | DRD2/CHRNA4/HTR2A/DRD1/ADRA1A/HTR1B | 6 |
| BP | GO:0033604 | negative regulation of catecholamine secretion | 5.14E-08 | 0.000000963 | DRD2/ADRA2A/ADRA2C/ADRA2B | 4 |
| BP | GO:0060134 | prepulse inhibition | 5.14E-08 | 0.000000963 | DRD2/DRD1/SLC6A3/DRD3 | 4 |
| BP | GO:0042596 | fear response | 5.58E-08 | 0.00000104 | HTR1A/DRD4/DRD1/HTR2C/ADRB1 | 5 |
| BP | GO:0006837 | serotonin transport | 6.84E-08 | 0.00000126 | SLC6A4/HTR1A/SLC18A2/HTR1B | 4 |
| BP | GO:0070374 | positive regulation of ERK1 and ERK2 cascade | 0.000000069 | 0.00000126 | TNF/DRD2/OPRM1/HTR2A/ADRA1A/FGF2/HTR2C/KDR | 8 |
| BP | GO:1904705 | regulation of vascular smooth muscle cell proliferation | 7.84E-08 | 0.00000141 | TNF/FGF2/HTR1B/PPARG/JAK2/MMP2 | 6 |
| BP | GO:1990874 | vascular smooth muscle cell proliferation | 7.84E-08 | 0.00000141 | TNF/FGF2/HTR1B/PPARG/JAK2/MMP2 | 6 |
| BP | GO:0007612 | learning | 8.31E-08 | 0.00000149 | TH/DRD2/DRD1/DRD3/OPRK1/BCHE/DRD5 | 7 |
| BP | GO:0051047 | positive regulation of secretion | 0.000000108 | 0.00000192 | TNF/SLC6A4/DRD2/ACHE/DRD3/OPRK1/IL2/ADORA1/JAK2/CYP19A1 | 10 |
| BP | GO:0006816 | calcium ion transport | 0.000000123 | 0.00000217 | DRD2/CHRNA4/OPRM1/DRD4/HTR2A/ADRA2A/DRD1/ADRA1A/FGF2/HTR2C | 10 |
| BP | GO:0046677 | response to antibiotic | 0.000000128 | 0.00000223 | TH/DRD2/OPRM1/DRD4/SLC6A3/OPRK1/HTR1B/IL2/JAK2 | 9 |
| BP | GO:0035249 | synaptic transmission, glutamatergic | 0.000000135 | 0.00000233 | TNF/DRD2/HTR2A/DRD1/HTR1B/ADORA1 | 6 |
| BP | GO:0043271 | negative regulation of ion transport | 0.000000143 | 0.00000247 | DRD2/DRD4/HTR2A/ADRA2A/DRD3/HTR1B/ADORA1 | 7 |
| BP | GO:0003085 | negative regulation of systemic arterial blood pressure | 0.000000145 | 0.00000248 | ADRA1A/ADRB2/ADORA1/ADRB1 | 4 |
| BP | GO:0035094 | response to nicotine | 0.000000159 | 0.00000268 | TNF/TH/DRD2/CHRNA4/SLC6A3 | 5 |
| BP | GO:1904707 | positive regulation of vascular smooth muscle cell proliferation | 0.000000159 | 0.00000268 | TNF/FGF2/HTR1B/JAK2/MMP2 | 5 |
| BP | GO:1903305 | regulation of regulated secretory pathway | 0.000000163 | 0.00000273 | DRD2/CHRNA4/HTR2A/ADRA2A/DRD1/ADRA1A/HTR1B | 7 |
| BP | GO:0043405 | regulation of MAP kinase activity | 0.000000165 | 0.00000274 | TNF/VEGFA/DRD4/HTR2A/ADRA2A/ADRA2B/FGF2/ADORA1/JAK2 | 9 |
| BP | GO:0042053 | regulation of dopamine metabolic process | 0.000000181 | 0.00000294 | HTR1A/DRD4/DRD1/SLC6A3 | 4 |
| BP | GO:0042069 | regulation of catecholamine metabolic process | 0.000000181 | 0.00000294 | HTR1A/DRD4/DRD1/SLC6A3 | 4 |
| BP | GO:0051580 | regulation of neurotransmitter uptake | 0.000000181 | 0.00000294 | DRD2/DRD4/DRD1/DRD3 | 4 |
| BP | GO:0045744 | negative regulation of G protein-coupled receptor signaling pathway | 0.000000195 | 0.00000315 | DRD2/OPRM1/ADRB2/DRD3/HTR1B | 5 |
| BP | GO:0048638 | regulation of developmental growth | 0.000000211 | 0.00000339 | SLC6A4/DRD2/VEGFA/PIK3CA/SLC6A3/ADRB2/DRD3/FGF2/ADRB1 | 9 |
| BP | GO:0002029 | desensitization of G protein-coupled receptor signaling pathway | 0.000000223 | 0.0000035 | DRD2/ADRB2/DRD3/HTR1B | 4 |
| BP | GO:0015874 | norepinephrine transport | 0.000000223 | 0.0000035 | ADRA2A/SLC6A2/ADRA2C/ADRA2B | 4 |
| BP | GO:0022401 | negative adaptation of signaling pathway | 0.000000223 | 0.0000035 | DRD2/ADRB2/DRD3/HTR1B | 4 |
| BP | GO:0008016 | regulation of heart contraction | 0.000000226 | 0.00000353 | TH/DRD2/ADRA1B/ADRA1A/IL2/ADORA1/ADRB1/JAK2 | 8 |
| BP | GO:0050806 | positive regulation of synaptic transmission | 0.000000227 | 0.00000353 | TNF/DRD2/DRD4/DRD1/ADRA1A/ADRB2/ADORA1 | 7 |
| BP | GO:0007269 | neurotransmitter secretion | 0.000000247 | 0.00000378 | DRD2/CHRNA4/HTR2A/DRD1/SLC18A2/ADRA1A/HTR1B | 7 |
| BP | GO:0099643 | signal release from synapse | 0.000000247 | 0.00000378 | DRD2/CHRNA4/HTR2A/DRD1/SLC18A2/ADRA1A/HTR1B | 7 |
| BP | GO:0006937 | regulation of muscle contraction | 0.000000257 | 0.0000039 | ADRA1B/ADRA2A/ADRA2C/ADRA1A/ADRB2/ADRA2B/ADORA1 | 7 |
| BP | GO:0019233 | sensory perception of pain | 0.000000262 | 0.00000394 | CHRNA4/OPRM1/HTR2A/ADRA2C/OPRK1/ADORA1 | 6 |
| BP | GO:0046928 | regulation of neurotransmitter secretion | 0.000000262 | 0.00000394 | DRD2/CHRNA4/HTR2A/DRD1/ADRA1A/HTR1B | 6 |
| BP | GO:0050708 | regulation of protein secretion | 0.000000268 | 0.000004 | TNF/DRD2/ACHE/OPRM1/DRD4/ADRA2A/ADRA2C/DRD3/IL2/JAK2 | 10 |
| BP | GO:0023058 | adaptation of signaling pathway | 0.000000272 | 0.00000403 | DRD2/ADRB2/DRD3/HTR1B | 4 |
| BP | GO:0043266 | regulation of potassium ion transport | 0.000000278 | 0.00000409 | DRD2/HTR2A/ADRA2A/DRD1/OPRK1/ADORA1 | 6 |
| BP | GO:0042136 | neurotransmitter biosynthetic process | 0.000000294 | 0.0000043 | TNF/TH/SLC6A4/ACHE/SLC6A3/JAK2 | 6 |
| BP | GO:0070838 | divalent metal ion transport | 0.000000307 | 0.00000447 | DRD2/CHRNA4/OPRM1/DRD4/HTR2A/ADRA2A/DRD1/ADRA1A/FGF2/HTR2C | 10 |
| BP | GO:0099565 | chemical synaptic transmission, postsynaptic | 0.000000328 | 0.00000475 | DRD2/CHRNA4/OPRM1/DRD4/ADRB2/ADORA1 | 6 |
| BP | GO:0072511 | divalent inorganic cation transport | 0.000000351 | 0.00000504 | DRD2/CHRNA4/OPRM1/DRD4/HTR2A/ADRA2A/DRD1/ADRA1A/FGF2/HTR2C | 10 |
| BP | GO:0031623 | receptor internalization | 0.000000386 | 0.00000551 | DRD2/VEGFA/ACHE/DRD4/DRD3/HTR1B | 6 |
| BP | GO:0098693 | regulation of synaptic vesicle cycle | 0.000000407 | 0.00000578 | DRD2/CHRNA4/HTR2A/DRD1/ADRA1A/HTR1B | 6 |
| BP | GO:0042752 | regulation of circadian rhythm | 0.000000452 | 0.00000638 | DRD2/DRD4/MAPK8/DRD3/PPARG/ADORA1 | 6 |
| BP | GO:0048545 | response to steroid hormone | 0.000000485 | 0.0000068 | TNF/TH/ESR1/BCHE/NR3C1/HTR1B/PPARG/JAK2/HDAC1 | 9 |
| BP | GO:0042745 | circadian sleep/wake cycle | 0.000000551 | 0.00000769 | TH/DRD2/DRD3/ADORA1 | 4 |
| BP | GO:0016079 | synaptic vesicle exocytosis | 0.000000612 | 0.00000846 | DRD2/CHRNA4/HTR2A/DRD1/ADRA1A/HTR1B | 6 |
| BP | GO:0010959 | regulation of metal ion transport | 0.000000614 | 0.00000846 | DRD2/DRD4/HTR2A/ADRA2A/DRD1/ADRB2/DRD3/OPRK1/ADORA1 | 9 |
| BP | GO:0001964 | startle response | 0.000000646 | 0.00000885 | DRD2/DRD1/SLC6A3/DRD3 | 4 |
| BP | GO:0032102 | negative regulation of response to external stimulus | 0.000000696 | 0.00000948 | DRD2/DRD1/SLC6A3/DRD3/FGF2/IL2/PPARG/ADORA1/CYP19A1 | 9 |
| BP | GO:0045822 | negative regulation of heart contraction | 0.000000871 | 0.0000118 | ADRA1A/IL2/ADORA1/JAK2 | 4 |
| BP | GO:0070372 | regulation of ERK1 and ERK2 cascade | 0.000000874 | 0.0000118 | TNF/DRD2/OPRM1/HTR2A/ADRA1A/FGF2/HTR2C/KDR | 8 |
| BP | GO:0015909 | long-chain fatty acid transport | 0.000000901 | 0.0000121 | DRD2/ACE/DRD4/DRD3/PPARG | 5 |
| BP | GO:0019098 | reproductive behavior | 0.000001 | 0.0000134 | TH/DRD1/OPRK1/DRD5 | 4 |
| BP | GO:0030431 | sleep | 0.00000115 | 0.000015 | DRD2/HTR2A/DRD3/ADORA1 | 4 |
| BP | GO:0050482 | arachidonic acid secretion | 0.00000115 | 0.000015 | DRD2/ACE/DRD4/DRD3 | 4 |
| BP | GO:1903963 | arachidonate transport | 0.00000115 | 0.000015 | DRD2/ACE/DRD4/DRD3 | 4 |
| BP | GO:1903524 | positive regulation of blood circulation | 0.00000119 | 0.0000155 | HTR2A/ADRA1B/ADRA2C/ADRA1A/ADRB1 | 5 |
| BP | GO:0051235 | maintenance of location | 0.00000126 | 0.0000163 | TNF/DRD2/HTR2A/DRD1/SLC18A2/FGF2/HTR2C/PPARG | 8 |
| BP | GO:0017157 | regulation of exocytosis | 0.00000128 | 0.0000165 | DRD2/CHRNA4/HTR2A/ADRA2A/DRD1/ADRA1A/HTR1B | 7 |
| BP | GO:0032228 | regulation of synaptic transmission, GABAergic | 0.00000131 | 0.0000166 | DRD2/ADRA1A/HTR1B/ADORA1 | 4 |
| BP | GO:0042311 | vasodilation | 0.00000131 | 0.0000166 | DRD1/ADRB2/ADORA1/ADRB1 | 4 |
| BP | GO:0045907 | positive regulation of vasoconstriction | 0.00000131 | 0.0000166 | HTR2A/ADRA1B/ADRA2C/ADRA1A | 4 |
| BP | GO:0070371 | ERK1 and ERK2 cascade | 0.00000132 | 0.0000167 | TNF/DRD2/OPRM1/HTR2A/ADRA1A/FGF2/HTR2C/KDR | 8 |
| BP | GO:0031644 | regulation of nervous system process | 0.00000133 | 0.0000167 | OPRM1/ADRA2C/ADRB2/OPRK1/HTR2C/ADORA1 | 6 |
| BP | GO:0070588 | calcium ion transmembrane transport | 0.00000139 | 0.0000173 | DRD2/OPRM1/DRD4/HTR2A/ADRA2A/DRD1/FGF2/HTR2C | 8 |
| BP | GO:0014072 | response to isoquinoline alkaloid | 0.00000149 | 0.0000183 | DRD2/OPRM1/DRD3/OPRK1 | 4 |
| BP | GO:0043278 | response to morphine | 0.00000149 | 0.0000183 | DRD2/OPRM1/DRD3/OPRK1 | 4 |
| BP | GO:2001025 | positive regulation of response to drug | 0.00000149 | 0.0000183 | DRD2/DRD4/DRD3/OPRK1 | 4 |
| BP | GO:0050709 | negative regulation of protein secretion | 0.00000158 | 0.0000192 | TNF/DRD2/OPRM1/DRD4/ADRA2A/DRD3 | 6 |
| BP | GO:0008306 | associative learning | 0.00000166 | 0.0000202 | DRD2/DRD1/DRD3/OPRK1/DRD5 | 5 |
| BP | GO:0001820 | serotonin secretion | 0.00000189 | 0.0000226 | SLC6A4/HTR1A/HTR1B | 3 |
| BP | GO:0042756 | drinking behavior | 0.00000189 | 0.0000226 | DRD2/REN/HTR1B | 3 |
| BP | GO:0048149 | behavioral response to ethanol | 0.00000189 | 0.0000226 | DRD2/OPRM1/DRD4 | 3 |
| BP | GO:0035264 | multicellular organism growth | 0.00000193 | 0.0000229 | DRD2/PIK3CA/SLC6A3/ADRB2/DRD3/ADRB1 | 6 |
| BP | GO:0045834 | positive regulation of lipid metabolic process | 0.00000193 | 0.0000229 | TNF/HTR2A/FGF2/HTR2C/PPARG/ADORA1 | 6 |
| BP | GO:0002792 | negative regulation of peptide secretion | 0.00000209 | 0.0000246 | TNF/DRD2/OPRM1/DRD4/ADRA2A/DRD3 | 6 |
| BP | GO:1904062 | regulation of cation transmembrane transport | 0.00000233 | 0.0000273 | DRD2/OPRM1/DRD4/ADRA2A/DRD1/ADRB2/DRD3/OPRK1 | 8 |
| BP | GO:0001101 | response to acid chemical | 0.00000238 | 0.0000275 | TNF/TH/SLC6A4/VEGFA/BCHE/PPARG/KDR/MMP2 | 8 |
| BP | GO:0001662 | behavioral fear response | 0.00000239 | 0.0000275 | HTR1A/DRD4/DRD1/HTR2C | 4 |
| BP | GO:0098926 | postsynaptic signal transduction | 0.00000239 | 0.0000275 | CHRNA4/OPRM1/CHRM1/JAK2 | 4 |
| BP | GO:0050886 | endocrine process | 0.0000024 | 0.0000276 | REN/ACE/DRD3/OPRK1/DRD5 | 5 |
| BP | GO:0007195 | adenylate cyclase-inhibiting dopamine receptor signaling pathway | 0.00000259 | 0.0000295 | DRD2/DRD4/DRD3 | 3 |
| BP | GO:0034776 | response to histamine | 0.00000259 | 0.0000295 | DRD2/DRD4/DRD3 | 3 |
| BP | GO:0002209 | behavioral defense response | 0.00000266 | 0.0000298 | HTR1A/DRD4/DRD1/HTR2C | 4 |
| BP | GO:0007190 | activation of adenylate cyclase activity | 0.00000266 | 0.0000298 | DRD1/ADRB2/DRD5/ADRB1 | 4 |
| BP | GO:1903523 | negative regulation of blood circulation | 0.00000266 | 0.0000298 | ADRA1A/IL2/ADORA1/JAK2 | 4 |
| BP | GO:0050769 | positive regulation of neurogenesis | 0.00000283 | 0.0000315 | TNF/DRD2/VEGFA/OPRM1/ADRA2C/ADRA2B/IL2/PPARG/HDAC1 | 9 |
| BP | GO:0051930 | regulation of sensory perception of pain | 0.00000296 | 0.0000329 | OPRM1/ADRA2C/OPRK1/ADORA1 | 4 |
| BP | GO:0060402 | calcium ion transport into cytosol | 0.00000306 | 0.0000338 | DRD2/HTR2A/DRD1/ADRA1A/FGF2/HTR2C | 6 |
| BP | GO:0001990 | regulation of systemic arterial blood pressure by hormone | 0.00000328 | 0.0000358 | REN/ACE/DRD3/DRD5 | 4 |
| BP | GO:0007618 | mating | 0.00000328 | 0.0000358 | TH/SLC6A4/DRD1/DRD5 | 4 |
| BP | GO:0051931 | regulation of sensory perception | 0.00000328 | 0.0000358 | OPRM1/ADRA2C/OPRK1/ADORA1 | 4 |
| BP | GO:0007589 | body fluid secretion | 0.00000398 | 0.0000431 | VEGFA/SLC6A3/OPRK1/CHRM1/ADORA1 | 5 |
| BP | GO:0090257 | regulation of muscle system process | 0.00000413 | 0.0000446 | ADRA1B/ADRA2A/ADRA2C/ADRA1A/ADRB2/ADRA2B/ADORA1 | 7 |
| BP | GO:0031667 | response to nutrient levels | 0.0000043 | 0.0000462 | TH/SLC6A4/OPRM1/MAPK8/ADRB2/BCHE/HTR2C/PPARG/ADRB1 | 9 |
| BP | GO:0010863 | positive regulation of phospholipase C activity | 0.00000441 | 0.0000472 | ESR1/HTR2A/ADRA1A/FGF2 | 4 |
| BP | GO:0003084 | positive regulation of systemic arterial blood pressure | 0.00000448 | 0.0000476 | ACE/ADRA1A/ADORA1 | 3 |
| BP | GO:0010469 | regulation of signaling receptor activity | 0.00000451 | 0.0000476 | OPRM1/ADRA2A/ADRA2C/ADRB2/ADRA2B/ADORA1 | 6 |
| BP | GO:0048660 | regulation of smooth muscle cell proliferation | 0.00000451 | 0.0000476 | TNF/FGF2/HTR1B/PPARG/JAK2/MMP2 | 6 |
| BP | GO:0048659 | smooth muscle cell proliferation | 0.00000482 | 0.0000507 | TNF/FGF2/HTR1B/PPARG/JAK2/MMP2 | 6 |
| BP | GO:0015908 | fatty acid transport | 0.00000489 | 0.000051 | DRD2/ACE/DRD4/DRD3/PPARG | 5 |
| BP | GO:0120162 | positive regulation of cold-induced thermogenesis | 0.00000489 | 0.000051 | VEGFA/ACHE/ADRB2/ADRB1/JAK2 | 5 |
| BP | GO:0043491 | protein kinase B signaling | 0.0000053 | 0.0000542 | TNF/DRD2/ESR1/PIK3CA/DRD3/FGF2/KDR | 7 |
| BP | GO:0032309 | icosanoid secretion | 0.0000053 | 0.0000542 | DRD2/ACE/DRD4/DRD3 | 4 |
| BP | GO:0051932 | synaptic transmission, GABAergic | 0.0000053 | 0.0000542 | DRD2/ADRA1A/HTR1B/ADORA1 | 4 |
| BP | GO:1900274 | regulation of phospholipase C activity | 0.0000053 | 0.0000542 | ESR1/HTR2A/ADRA1A/FGF2 | 4 |
| BP | GO:0060079 | excitatory postsynaptic potential | 0.00000569 | 0.0000578 | DRD2/CHRNA4/OPRM1/ADRB2/ADORA1 | 5 |
| BP | GO:0060401 | cytosolic calcium ion transport | 0.00000569 | 0.0000578 | DRD2/HTR2A/DRD1/ADRA1A/FGF2/HTR2C | 6 |
| BP | GO:0048512 | circadian behavior | 0.0000058 | 0.0000585 | TH/DRD2/DRD3/ADORA1 | 4 |
| BP | GO:0048661 | positive regulation of smooth muscle cell proliferation | 0.00000597 | 0.00006 | TNF/FGF2/HTR1B/JAK2/MMP2 | 5 |
| BP | GO:0007622 | rhythmic behavior | 0.00000632 | 0.0000633 | TH/DRD2/DRD3/ADORA1 | 4 |
| BP | GO:0070482 | response to oxygen levels | 0.00000661 | 0.0000659 | TH/SLC6A4/DRD2/VEGFA/CHRNA4/PPARG/ADORA1/MMP2 | 8 |
| BP | GO:0043268 | positive regulation of potassium ion transport | 0.00000689 | 0.0000684 | ADRA2A/DRD1/OPRK1/ADORA1 | 4 |
| BP | GO:2001028 | positive regulation of endothelial cell chemotaxis | 0.0000071 | 0.0000702 | VEGFA/FGF2/KDR | 3 |
| BP | GO:0032409 | regulation of transporter activity | 0.00000739 | 0.0000727 | DRD2/OPRM1/DRD4/ADRA2A/ADRB2/DRD3/PPARG | 7 |
| BP | GO:0071715 | icosanoid transport | 0.00000748 | 0.0000731 | DRD2/ACE/DRD4/DRD3 | 4 |
| BP | GO:1901571 | fatty acid derivative transport | 0.00000748 | 0.0000731 | DRD2/ACE/DRD4/DRD3 | 4 |
| BP | GO:1903532 | positive regulation of secretion by cell | 0.00000779 | 0.0000758 | TNF/SLC6A4/DRD2/ACHE/OPRK1/IL2/JAK2/CYP19A1 | 8 |
| BP | GO:0014061 | regulation of norepinephrine secretion | 0.00000872 | 0.0000838 | ADRA2A/ADRA2C/ADRA2B | 3 |
| BP | GO:0042447 | hormone catabolic process | 0.00000872 | 0.0000838 | ACHE/ACE/CYP19A1 | 3 |
| BP | GO:0045986 | negative regulation of smooth muscle contraction | 0.00000872 | 0.0000838 | ADRA2C/ADRB2/ADORA1 | 3 |
| BP | GO:0019216 | regulation of lipid metabolic process | 0.00000883 | 0.0000845 | TNF/HTR2A/ADRA2A/DRD3/FGF2/HTR2C/PPARG/ADORA1 | 8 |
| BP | GO:0035176 | social behavior | 0.00000951 | 0.0000906 | TH/SLC6A4/DRD4/DRD3 | 4 |
| BP | GO:0010876 | lipid localization | 0.00000965 | 0.0000912 | TNF/DRD2/REN/ACE/DRD4/DRD3/PPARG/CYP19A1 | 8 |
| BP | GO:0051224 | negative regulation of protein transport | 0.00000966 | 0.0000912 | TNF/DRD2/OPRM1/DRD4/ADRA2A/DRD3 | 6 |
| BP | GO:0002031 | G protein-coupled receptor internalization | 0.0000106 | 0.0000983 | DRD2/DRD3/HTR1B | 3 |
| BP | GO:0031649 | heat generation | 0.0000106 | 0.0000983 | TNF/ADRB2/ADRB1 | 3 |
| BP | GO:0045187 | regulation of circadian sleep/wake cycle, sleep | 0.0000106 | 0.0000983 | DRD2/DRD3/ADORA1 | 3 |
| BP | GO:0048243 | norepinephrine secretion | 0.0000106 | 0.0000983 | ADRA2A/ADRA2C/ADRA2B | 3 |
| BP | GO:1904950 | negative regulation of establishment of protein localization | 0.0000109 | 0.000101 | TNF/DRD2/OPRM1/DRD4/ADRA2A/DRD3 | 6 |
| BP | GO:0051703 | intraspecies interaction between organisms | 0.0000111 | 0.000102 | TH/SLC6A4/DRD4/DRD3 | 4 |
| BP | GO:0002090 | regulation of receptor internalization | 0.0000119 | 0.000109 | DRD2/VEGFA/DRD4/DRD3 | 4 |
| BP | GO:0061098 | positive regulation of protein tyrosine kinase activity | 0.0000119 | 0.000109 | ACE/ADRA2A/ADRA1A/ADORA1 | 4 |
| BP | GO:0007613 | memory | 0.0000122 | 0.000112 | TH/SLC6A4/DRD2/HTR2A/DRD1 | 5 |
| BP | GO:0048732 | gland development | 0.0000133 | 0.000121 | TNF/DRD2/VEGFA/ESR1/PIK3CA/SLC6A3/JAK2/CYP19A1 | 8 |
| BP | GO:0003014 | renal system process | 0.0000138 | 0.000125 | DRD2/REN/ADRA1A/DRD3/ADORA1 | 5 |
| BP | GO:0006898 | receptor-mediated endocytosis | 0.0000145 | 0.000131 | DRD2/VEGFA/ACHE/DRD4/ADRB2/DRD3/HTR1B | 7 |
| BP | GO:0090276 | regulation of peptide hormone secretion | 0.0000148 | 0.000133 | TNF/DRD2/ADRA2A/ADRA2C/HTR2C/JAK2 | 6 |
| BP | GO:0071229 | cellular response to acid chemical | 0.0000152 | 0.000136 | TNF/SLC6A4/VEGFA/PPARG/KDR/MMP2 | 6 |
| BP | GO:0010518 | positive regulation of phospholipase activity | 0.0000158 | 0.00014 | ESR1/HTR2A/ADRA1A/FGF2 | 4 |
| BP | GO:0097755 | positive regulation of blood vessel diameter | 0.0000158 | 0.00014 | DRD1/ADRB2/ADORA1/ADRB1 | 4 |
| BP | GO:0014013 | regulation of gliogenesis | 0.0000162 | 0.000142 | TNF/DRD3/CHRM1/PPARG/HDAC1 | 5 |
| BP | GO:0046717 | acid secretion | 0.0000162 | 0.000142 | DRD2/ACE/DRD4/DRD3/ADORA1 | 5 |
| BP | GO:0051209 | release of sequestered calcium ion into cytosol | 0.0000162 | 0.000142 | DRD2/HTR2A/DRD1/FGF2/HTR2C | 5 |
| BP | GO:0051283 | negative regulation of sequestering of calcium ion | 0.0000175 | 0.000153 | DRD2/HTR2A/DRD1/FGF2/HTR2C | 5 |
| BP | GO:0050802 | circadian sleep/wake cycle, sleep | 0.0000176 | 0.000154 | DRD2/DRD3/ADORA1 | 3 |
| BP | GO:0034763 | negative regulation of transmembrane transport | 0.0000189 | 0.000164 | TNF/DRD2/DRD4/ADRA2A/DRD3 | 5 |
| BP | GO:0051282 | regulation of sequestering of calcium ion | 0.0000189 | 0.000164 | DRD2/HTR2A/DRD1/FGF2/HTR2C | 5 |
| BP | GO:0044106 | cellular amine metabolic process | 0.0000197 | 0.000169 | MAOA/HTR1A/DRD4/DRD1/SLC6A3 | 5 |
| BP | GO:0032496 | response to lipopolysaccharide | 0.00002 | 0.000172 | TNF/TH/OPRM1/REN/MAPK8/OPRK1/JAK2 | 7 |
| BP | GO:0007588 | excretion | 0.0000205 | 0.000172 | DRD2/ADRA1A/DRD3/ADORA1 | 4 |
| BP | GO:0046888 | negative regulation of hormone secretion | 0.0000205 | 0.000172 | DRD2/ADRA2A/OPRK1/ADORA1 | 4 |
| BP | GO:0014821 | phasic smooth muscle contraction | 0.0000205 | 0.000172 | DRD2/DRD1/HTR1D | 3 |
| BP | GO:0035813 | regulation of renal sodium excretion | 0.0000205 | 0.000172 | DRD2/DRD3/ADORA1 | 3 |
| BP | GO:0042749 | regulation of circadian sleep/wake cycle | 0.0000205 | 0.000172 | DRD2/DRD3/ADORA1 | 3 |
| BP | GO:0050951 | sensory perception of temperature stimulus | 0.0000205 | 0.000172 | HTR2A/OPRK1/ADORA1 | 3 |
| BP | GO:0051208 | sequestering of calcium ion | 0.0000212 | 0.000177 | DRD2/HTR2A/DRD1/FGF2/HTR2C | 5 |
| BP | GO:0045444 | fat cell differentiation | 0.0000219 | 0.000183 | TNF/HTR2A/ADRB2/HTR2C/PPARG/ADRB1 | 6 |
| BP | GO:0007617 | mating behavior | 0.0000237 | 0.000196 | TH/DRD1/DRD5 | 3 |
| BP | GO:0010869 | regulation of receptor biosynthetic process | 0.0000237 | 0.000196 | PPARG/JAK2/HDAC1 | 3 |
| BP | GO:0002237 | response to molecule of bacterial origin | 0.0000256 | 0.000211 | TNF/TH/OPRM1/REN/MAPK8/OPRK1/JAK2 | 7 |
| BP | GO:0009308 | amine metabolic process | 0.0000272 | 0.000222 | MAOA/HTR1A/DRD4/DRD1/SLC6A3 | 5 |
| BP | GO:0022410 | circadian sleep/wake cycle process | 0.0000272 | 0.000222 | DRD2/DRD3/ADORA1 | 3 |
| BP | GO:0035812 | renal sodium excretion | 0.0000272 | 0.000222 | DRD2/DRD3/ADORA1 | 3 |
| BP | GO:0050995 | negative regulation of lipid catabolic process | 0.0000272 | 0.000222 | TNF/ADRA2A/ADORA1 | 3 |
| BP | GO:0009755 | hormone-mediated signaling pathway | 0.000028 | 0.000227 | ESR1/REN/NR3C1/PPARG/JAK2/HDAC1 | 6 |
| BP | GO:0042445 | hormone metabolic process | 0.000028 | 0.000227 | TNF/ACHE/ESR1/REN/ACE/CYP19A1 | 6 |
| BP | GO:0034765 | regulation of ion transmembrane transport | 0.0000288 | 0.000232 | DRD2/OPRM1/DRD4/ADRA2A/DRD1/ADRB2/DRD3/OPRK1 | 8 |
| BP | GO:0097553 | calcium ion transmembrane import into cytosol | 0.0000292 | 0.000234 | DRD2/HTR2A/DRD1/FGF2/HTR2C | 5 |
| BP | GO:0044062 | regulation of excretion | 0.0000311 | 0.000247 | DRD2/DRD3/ADORA1 | 3 |
| BP | GO:2001026 | regulation of endothelial cell chemotaxis | 0.0000311 | 0.000247 | VEGFA/FGF2/KDR | 3 |
| BP | GO:0010517 | regulation of phospholipase activity | 0.0000311 | 0.000247 | ESR1/HTR2A/ADRA1A/FGF2 | 4 |
| BP | GO:0008277 | regulation of G protein-coupled receptor signaling pathway | 0.0000312 | 0.000247 | DRD2/OPRM1/ADRB2/DRD3/HTR1B | 5 |
| BP | GO:0030879 | mammary gland development | 0.0000323 | 0.000253 | VEGFA/ESR1/SLC6A3/JAK2/CYP19A1 | 5 |
| BP | GO:0106106 | cold-induced thermogenesis | 0.0000323 | 0.000253 | VEGFA/ACHE/ADRB2/ADRB1/JAK2 | 5 |
| BP | GO:0120161 | regulation of cold-induced thermogenesis | 0.0000323 | 0.000253 | VEGFA/ACHE/ADRB2/ADRB1/JAK2 | 5 |
| BP | GO:0033002 | muscle cell proliferation | 0.0000323 | 0.000253 | TNF/FGF2/HTR1B/PPARG/JAK2/MMP2 | 6 |
| BP | GO:0006813 | potassium ion transport | 0.0000331 | 0.000258 | DRD2/HTR2A/ADRA2A/DRD1/OPRK1/ADORA1 | 6 |
| BP | GO:0042593 | glucose homeostasis | 0.0000339 | 0.000263 | TH/PIK3CA/ADRA1B/ADRA2A/OPRK1/PPARG | 6 |
| BP | GO:0001666 | response to hypoxia | 0.0000343 | 0.000265 | TH/SLC6A4/DRD2/VEGFA/CHRNA4/ADORA1/MMP2 | 7 |
| BP | GO:0033500 | carbohydrate homeostasis | 0.0000347 | 0.000267 | TH/PIK3CA/ADRA1B/ADRA2A/OPRK1/PPARG | 6 |
| BP | GO:0060193 | positive regulation of lipase activity | 0.0000348 | 0.000267 | ESR1/HTR2A/ADRA1A/FGF2 | 4 |
| BP | GO:0009266 | response to temperature stimulus | 0.0000355 | 0.000272 | HTR2A/ADRB2/HTR1B/PPARG/ADORA1/ADRB1 | 6 |
| BP | GO:0051896 | regulation of protein kinase B signaling | 0.0000363 | 0.000277 | TNF/DRD2/ESR1/PIK3CA/DRD3/FGF2 | 6 |
| BP | GO:0014065 | phosphatidylinositol 3-kinase signaling | 0.0000381 | 0.00029 | TNF/PIK3CA/HTR2A/KDR/JAK2 | 5 |
| BP | GO:0003081 | regulation of systemic arterial blood pressure by renin-angiotensin | 0.0000398 | 0.000302 | REN/ACE/DRD3 | 3 |
| BP | GO:0036293 | response to decreased oxygen levels | 0.0000415 | 0.000313 | TH/SLC6A4/DRD2/VEGFA/CHRNA4/ADORA1/MMP2 | 7 |
| BP | GO:0030072 | peptide hormone secretion | 0.0000416 | 0.000313 | TNF/DRD2/ADRA2A/ADRA2C/HTR2C/JAK2 | 6 |
| BP | GO:0050727 | regulation of inflammatory response | 0.0000444 | 0.000333 | TNF/ESR1/IL2/PPARG/ADORA1/JAK2/CYP19A1 | 7 |
| BP | GO:0035902 | response to immobilization stress | 0.0000447 | 0.000333 | TH/REN/PPARG | 3 |
| BP | GO:0045932 | negative regulation of muscle contraction | 0.0000447 | 0.000333 | ADRA2C/ADRB2/ADORA1 | 3 |
| BP | GO:1990845 | adaptive thermogenesis | 0.000046 | 0.000342 | VEGFA/ACHE/ADRB2/ADRB1/JAK2 | 5 |
| BP | GO:0032413 | negative regulation of ion transmembrane transporter activity | 0.0000477 | 0.000354 | DRD2/DRD4/ADRA2A/DRD3 | 4 |
| BP | GO:0006869 | lipid transport | 0.0000491 | 0.000363 | DRD2/REN/ACE/DRD4/DRD3/PPARG/CYP19A1 | 7 |
| BP | GO:0007205 | protein kinase C-activating G protein-coupled receptor signaling pathway | 0.0000499 | 0.000367 | CHRM1/HTR1B/IL2 | 3 |
| BP | GO:0095500 | acetylcholine receptor signaling pathway | 0.0000499 | 0.000367 | CHRNA4/OPRM1/CHRM1 | 3 |
| BP | GO:0032412 | regulation of ion transmembrane transporter activity | 0.0000517 | 0.000379 | DRD2/OPRM1/DRD4/ADRA2A/ADRB2/DRD3 | 6 |
| BP | GO:0033238 | regulation of cellular amine metabolic process | 0.0000553 | 0.000404 | HTR1A/DRD4/DRD1/SLC6A3 | 4 |
| BP | GO:0031960 | response to corticosteroid | 0.0000586 | 0.000426 | TNF/TH/BCHE/NR3C1/HTR1B | 5 |
| BP | GO:0015718 | monocarboxylic acid transport | 0.0000603 | 0.000437 | DRD2/ACE/DRD4/DRD3/PPARG | 5 |
| BP | GO:0022898 | regulation of transmembrane transporter activity | 0.0000612 | 0.000443 | DRD2/OPRM1/DRD4/ADRA2A/ADRB2/DRD3 | 6 |
| BP | GO:1905144 | response to acetylcholine | 0.0000617 | 0.000443 | CHRNA4/OPRM1/CHRM1 | 3 |
| BP | GO:1905145 | cellular response to acetylcholine | 0.0000617 | 0.000443 | CHRNA4/OPRM1/CHRM1 | 3 |
| BP | GO:0002028 | regulation of sodium ion transport | 0.0000668 | 0.000479 | DRD2/DRD4/ADRB2/DRD3 | 4 |
| BP | GO:0035767 | endothelial cell chemotaxis | 0.0000682 | 0.000485 | VEGFA/FGF2/KDR | 3 |
| BP | GO:0060292 | long-term synaptic depression | 0.0000682 | 0.000485 | DRD1/DRD5/ADORA1 | 3 |
| BP | GO:0014068 | positive regulation of phosphatidylinositol 3-kinase signaling | 0.0000732 | 0.000518 | TNF/PIK3CA/KDR/JAK2 | 4 |
| BP | GO:0051781 | positive regulation of cell division | 0.0000732 | 0.000518 | DRD2/VEGFA/DRD3/FGF2 | 4 |
| BP | GO:0045987 | positive regulation of smooth muscle contraction | 0.0000751 | 0.00053 | ADRA1B/ADRA1A/ADRA2B | 3 |
| BP | GO:0061097 | regulation of protein tyrosine kinase activity | 0.0000765 | 0.000538 | ACE/ADRA2A/ADRA1A/ADORA1 | 4 |
| BP | GO:0034329 | cell junction assembly | 0.000078 | 0.000547 | TNF/DRD2/VEGFA/ACHE/ACE/DRD1/KDR | 7 |
| BP | GO:0032410 | negative regulation of transporter activity | 0.00008 | 0.000559 | DRD2/DRD4/ADRA2A/DRD3 | 4 |
| BP | GO:0042755 | eating behavior | 0.0000824 | 0.000575 | TH/OPRM1/OPRK1 | 3 |
| BP | GO:0001936 | regulation of endothelial cell proliferation | 0.0000867 | 0.000601 | TNF/VEGFA/FGF2/PPARG/KDR | 5 |
| BP | GO:0050796 | regulation of insulin secretion | 0.0000867 | 0.000601 | TNF/DRD2/ADRA2A/ADRA2C/JAK2 | 5 |
| BP | GO:1904063 | negative regulation of cation transmembrane transport | 0.0000872 | 0.000603 | DRD2/DRD4/ADRA2A/DRD3 | 4 |
| BP | GO:0032148 | activation of protein kinase B activity | 0.0000902 | 0.00062 | ADRA2A/ADRA2C/ADRA2B | 3 |
| BP | GO:0070050 | neuron cellular homeostasis | 0.0000902 | 0.00062 | SLC6A2/HTR1B/ADORA1 | 3 |
| BP | GO:0002793 | positive regulation of peptide secretion | 0.0000911 | 0.000624 | TNF/DRD2/ACHE/IL2/ADORA1/JAK2 | 6 |
| BP | GO:0048771 | tissue remodeling | 0.0000939 | 0.000642 | ACE/ADRB2/HTR1B/IL2/MMP2 | 5 |
| BP | GO:0042063 | gliogenesis | 0.0000946 | 0.000644 | TNF/DRD1/DRD3/CHRM1/PPARG/HDAC1 | 6 |
| BP | GO:0051954 | positive regulation of amine transport | 0.0000985 | 0.000665 | DRD2/DRD4/OPRK1 | 3 |
| BP | GO:0071312 | cellular response to alkaloid | 0.0000985 | 0.000665 | TH/OPRM1/HTR1B | 3 |
| BP | GO:0098801 | regulation of renal system process | 0.0000985 | 0.000665 | DRD2/DRD3/ADORA1 | 3 |
| BP | GO:0048015 | phosphatidylinositol-mediated signaling | 0.000099 | 0.000666 | TNF/PIK3CA/HTR2A/KDR/JAK2 | 5 |
| BP | GO:0043401 | steroid hormone mediated signaling pathway | 0.000102 | 0.000682 | ESR1/NR3C1/PPARG/JAK2/HDAC1 | 5 |
| BP | GO:0048017 | inositol lipid-mediated signaling | 0.000107 | 0.000716 | TNF/PIK3CA/HTR2A/KDR/JAK2 | 5 |
| BP | GO:1901020 | negative regulation of calcium ion transmembrane transporter activity | 0.000107 | 0.000716 | DRD2/DRD4/ADRA2A | 3 |
| BP | GO:0060191 | regulation of lipase activity | 0.000116 | 0.000769 | ESR1/HTR2A/ADRA1A/FGF2 | 4 |
| BP | GO:0032941 | secretion by tissue | 0.000117 | 0.000769 | OPRK1/CHRM1/ADORA1 | 3 |
| BP | GO:0038083 | peptidyl-tyrosine autophosphorylation | 0.000117 | 0.000769 | VEGFA/ACE/KDR | 3 |
| BP | GO:0090050 | positive regulation of cell migration involved in sprouting angiogenesis | 0.000117 | 0.000769 | VEGFA/FGF2/KDR | 3 |
| BP | GO:0051222 | positive regulation of protein transport | 0.000123 | 0.000809 | TNF/DRD2/ACHE/MAPK8/CHRM1/IL2/JAK2 | 7 |
| BP | GO:0022600 | digestive system process | 0.000126 | 0.000826 | ADRA2A/DRD3/OPRK1/CHRM1 | 4 |
| BP | GO:0051590 | positive regulation of neurotransmitter transport | 0.000126 | 0.000827 | SLC6A4/DRD2/DRD4 | 3 |
| BP | GO:0001935 | endothelial cell proliferation | 0.000127 | 0.000832 | TNF/VEGFA/FGF2/PPARG/KDR | 5 |
| BP | GO:0048259 | regulation of receptor-mediated endocytosis | 0.000131 | 0.000851 | DRD2/VEGFA/DRD4/DRD3 | 4 |
| BP | GO:0048713 | regulation of oligodendrocyte differentiation | 0.000137 | 0.000886 | DRD3/PPARG/HDAC1 | 3 |
| BP | GO:0009314 | response to radiation | 0.000137 | 0.00089 | TH/DRD2/OPRM1/DRD1/MAPK8/DRD3/OPRK1 | 7 |
| BP | GO:0034766 | negative regulation of ion transmembrane transport | 0.000141 | 0.00091 | DRD2/DRD4/ADRA2A/DRD3 | 4 |
| BP | GO:0001763 | morphogenesis of a branching structure | 0.000144 | 0.000926 | TNF/DRD2/VEGFA/ESR1/FGF2 | 5 |
| BP | GO:1904951 | positive regulation of establishment of protein localization | 0.000153 | 0.000985 | TNF/DRD2/ACHE/MAPK8/CHRM1/IL2/JAK2 | 7 |
| BP | GO:0050905 | neuromuscular process | 0.000163 | 0.001046 | DRD2/DRD1/SLC6A3/DRD3 | 4 |
| BP | GO:0002526 | acute inflammatory response | 0.000169 | 0.001081 | TNF/OPRM1/PPARG/ADORA1 | 4 |
| BP | GO:0035265 | organ growth | 0.000173 | 0.001104 | SLC6A4/ESR1/ADRA1A/FGF2/CYP19A1 | 5 |
| BP | GO:0021761 | limbic system development | 0.000176 | 0.001114 | DRD2/DRD1/HTR5A/HDAC1 | 4 |
| BP | GO:0030100 | regulation of endocytosis | 0.000177 | 0.001123 | DRD2/VEGFA/DRD4/DRD3/PPARG | 5 |
| BP | GO:1903170 | negative regulation of calcium ion transmembrane transport | 0.000183 | 0.001156 | DRD2/DRD4/ADRA2A | 3 |
| BP | GO:0030073 | insulin secretion | 0.000186 | 0.001169 | TNF/DRD2/ADRA2A/ADRA2C/JAK2 | 5 |
| BP | GO:0001655 | urogenital system development | 0.000191 | 0.001202 | VEGFA/ESR1/REN/ACE/FGF2/CYP19A1 | 6 |
| BP | GO:0046942 | carboxylic acid transport | 0.000194 | 0.001218 | DRD2/ACE/DRD4/DRD3/PPARG/ADORA1 | 6 |
| BP | GO:0015849 | organic acid transport | 0.000201 | 0.001255 | DRD2/ACE/DRD4/DRD3/PPARG/ADORA1 | 6 |
| BP | GO:0007596 | blood coagulation | 0.000211 | 0.001314 | PIK3CA/ADRA2A/ADRA2C/ADRA2B/JAK2/HDAC1 | 6 |
| BP | GO:0006661 | phosphatidylinositol biosynthetic process | 0.000223 | 0.001385 | PIK3CA/HTR2A/FGF2/HTR2C | 4 |
| BP | GO:0002673 | regulation of acute inflammatory response | 0.000224 | 0.001389 | TNF/PPARG/ADORA1 | 3 |
| BP | GO:0007599 | hemostasis | 0.000228 | 0.001412 | PIK3CA/ADRA2A/ADRA2C/ADRA2B/JAK2/HDAC1 | 6 |
| BP | GO:0046474 | glycerophospholipid biosynthetic process | 0.000231 | 0.001424 | ACHE/PIK3CA/HTR2A/FGF2/HTR2C | 5 |
| BP | GO:0050817 | coagulation | 0.000232 | 0.001427 | PIK3CA/ADRA2A/ADRA2C/ADRA2B/JAK2/HDAC1 | 6 |
| BP | GO:0010001 | glial cell differentiation | 0.000236 | 0.001447 | TNF/DRD1/DRD3/PPARG/HDAC1 | 5 |
| BP | GO:0050435 | amyloid-beta metabolic process | 0.000239 | 0.001461 | TNF/REN/ACE | 3 |
| BP | GO:0051651 | maintenance of location in cell | 0.000241 | 0.00147 | DRD2/HTR2A/DRD1/FGF2/HTR2C | 5 |
| BP | GO:0072089 | stem cell proliferation | 0.000254 | 0.001536 | DRD2/VEGFA/ACE/FGF2 | 4 |
| BP | GO:0008542 | visual learning | 0.000254 | 0.001536 | DRD2/DRD1/DRD3 | 3 |
| BP | GO:0045933 | positive regulation of muscle contraction | 0.000254 | 0.001536 | ADRA1B/ADRA1A/ADRA2B | 3 |
| BP | GO:1903727 | positive regulation of phospholipid metabolic process | 0.000254 | 0.001536 | HTR2A/FGF2/HTR2C | 3 |
| BP | GO:0044706 | multi-multicellular organism process | 0.000257 | 0.001538 | SLC6A4/ESR1/ADRA2C/ADRA2B/MMP2 | 5 |
| BP | GO:0006914 | autophagy | 0.000257 | 0.001538 | DRD2/PIK3CA/MAPK8/ADRA1A/ADRB2/DRD3/KDR | 7 |
| BP | GO:0061919 | process utilizing autophagic mechanism | 0.000257 | 0.001538 | DRD2/PIK3CA/MAPK8/ADRA1A/ADRB2/DRD3/KDR | 7 |
| BP | GO:0009409 | response to cold | 0.000287 | 0.001698 | ADRB2/PPARG/ADRB1 | 3 |
| BP | GO:0030432 | peristalsis | 0.000287 | 0.001698 | DRD2/DRD1 | 2 |
| BP | GO:0045741 | positive regulation of epidermal growth factor-activated receptor activity | 0.000287 | 0.001698 | ADRA2A/ADORA1 | 2 |
| BP | GO:0070472 | regulation of uterine smooth muscle contraction | 0.000287 | 0.001698 | ADRA2C/ADRA2B | 2 |
| BP | GO:0090037 | positive regulation of protein kinase C signaling | 0.000287 | 0.001698 | VEGFA/ADRA1A | 2 |
| BP | GO:0014066 | regulation of phosphatidylinositol 3-kinase signaling | 0.000288 | 0.001698 | TNF/PIK3CA/KDR/JAK2 | 4 |
| BP | GO:0055078 | sodium ion homeostasis | 0.000323 | 0.001899 | DRD2/DRD3/ADORA1 | 3 |
| BP | GO:0007632 | visual behavior | 0.000341 | 0.002005 | DRD2/DRD1/DRD3 | 3 |
| BP | GO:0030518 | intracellular steroid hormone receptor signaling pathway | 0.000344 | 0.002014 | ESR1/NR3C1/JAK2/HDAC1 | 4 |
| BP | GO:0001976 | nervous system process involved in regulation of systemic arterial blood pressure | 0.000351 | 0.002014 | DRD2/ADRA1A | 2 |
| BP | GO:0021892 | cerebral cortex GABAergic interneuron differentiation | 0.000351 | 0.002014 | DRD2/DRD1 | 2 |
| BP | GO:0031987 | locomotion involved in locomotory behavior | 0.000351 | 0.002014 | DRD2/DRD3 | 2 |
| BP | GO:0040015 | negative regulation of multicellular organism growth | 0.000351 | 0.002014 | ADRB2/ADRB1 | 2 |
| BP | GO:0060159 | regulation of dopamine receptor signaling pathway | 0.000351 | 0.002014 | DRD2/DRD3 | 2 |
| BP | GO:0060736 | prostate gland growth | 0.000351 | 0.002014 | ESR1/CYP19A1 | 2 |
| BP | GO:0070471 | uterine smooth muscle contraction | 0.000351 | 0.002014 | ADRA2C/ADRA2B | 2 |
| BP | GO:0099149 | regulation of postsynaptic neurotransmitter receptor internalization | 0.000351 | 0.002014 | DRD4/DRD3 | 2 |
| BP | GO:0031348 | negative regulation of defense response | 0.00036 | 0.002062 | DRD2/IL2/PPARG/ADORA1/CYP19A1 | 5 |
| BP | GO:0050994 | regulation of lipid catabolic process | 0.000361 | 0.002062 | TNF/ADRA2A/ADORA1 | 3 |
| BP | GO:0045598 | regulation of fat cell differentiation | 0.000365 | 0.002081 | TNF/HTR2A/HTR2C/PPARG | 4 |
| BP | GO:0071333 | cellular response to glucose stimulus | 0.000376 | 0.002136 | TH/PIK3CA/ADRA2A/OPRK1 | 4 |
| BP | GO:0032355 | response to estradiol | 0.000386 | 0.002193 | TH/SLC6A4/ESR1/HTR5A | 4 |
| BP | GO:0071331 | cellular response to hexose stimulus | 0.000398 | 0.00225 | TH/PIK3CA/ADRA2A/OPRK1 | 4 |
| BP | GO:0060688 | regulation of morphogenesis of a branching structure | 0.000402 | 0.002268 | TNF/VEGFA/ESR1 | 3 |
| BP | GO:0071326 | cellular response to monosaccharide stimulus | 0.000409 | 0.002303 | TH/PIK3CA/ADRA2A/OPRK1 | 4 |
| BP | GO:0030900 | forebrain development | 0.000413 | 0.002306 | TH/DRD2/DRD1/SLC6A3/HTR5A/HDAC1 | 6 |
| BP | GO:0002002 | regulation of angiotensin levels in blood | 0.00042 | 0.002306 | REN/ACE | 2 |
| BP | GO:0002003 | angiotensin maturation | 0.00042 | 0.002306 | REN/ACE | 2 |
| BP | GO:0002024 | diet induced thermogenesis | 0.00042 | 0.002306 | ADRB2/ADRB1 | 2 |
| BP | GO:0002674 | negative regulation of acute inflammatory response | 0.00042 | 0.002306 | PPARG/ADORA1 | 2 |
| BP | GO:0019336 | phenol-containing compound catabolic process | 0.00042 | 0.002306 | MAOA/SLC6A3 | 2 |
| BP | GO:0038003 | opioid receptor signaling pathway | 0.00042 | 0.002306 | OPRM1/OPRK1 | 2 |
| BP | GO:0042416 | dopamine biosynthetic process | 0.00042 | 0.002306 | TH/SLC6A3 | 2 |
| BP | GO:0046541 | saliva secretion | 0.00042 | 0.002306 | OPRK1/CHRM1 | 2 |
| BP | GO:0071073 | positive regulation of phospholipid biosynthetic process | 0.00042 | 0.002306 | HTR2A/HTR2C | 2 |
| BP | GO:0150065 | regulation of deacetylase activity | 0.00042 | 0.002306 | VEGFA/MAPK8 | 2 |
| BP | GO:0071383 | cellular response to steroid hormone stimulus | 0.000427 | 0.002336 | ESR1/NR3C1/PPARG/JAK2/HDAC1 | 5 |
| BP | GO:0021537 | telencephalon development | 0.000435 | 0.002374 | TH/DRD2/DRD1/HTR5A/HDAC1 | 5 |
| BP | GO:0007586 | digestion | 0.000444 | 0.002414 | ADRA2A/DRD3/OPRK1/CHRM1 | 4 |
| BP | GO:0035304 | regulation of protein dephosphorylation | 0.000444 | 0.002414 | TNF/DRD2/ADORA1/JAK2 | 4 |
| BP | GO:0045017 | glycerolipid biosynthetic process | 0.000451 | 0.002445 | ACHE/PIK3CA/HTR2A/FGF2/HTR2C | 5 |
| BP | GO:0060048 | cardiac muscle contraction | 0.000456 | 0.002469 | PIK3CA/ADRA1B/ADRA1A/ADORA1 | 4 |
| BP | GO:0010870 | positive regulation of receptor biosynthetic process | 0.000496 | 0.00264 | JAK2/HDAC1 | 2 |
| BP | GO:0014842 | regulation of skeletal muscle satellite cell proliferation | 0.000496 | 0.00264 | FGF2/JAK2 | 2 |
| BP | GO:0021984 | adenohypophysis development | 0.000496 | 0.00264 | DRD2/SLC6A3 | 2 |
| BP | GO:0042711 | maternal behavior | 0.000496 | 0.00264 | DRD1/OPRK1 | 2 |
| BP | GO:0060746 | parental behavior | 0.000496 | 0.00264 | DRD1/OPRK1 | 2 |
| BP | GO:1901550 | regulation of endothelial cell development | 0.000496 | 0.00264 | TNF/VEGFA | 2 |
| BP | GO:1903140 | regulation of establishment of endothelial barrier | 0.000496 | 0.00264 | TNF/VEGFA | 2 |
| BP | GO:0071322 | cellular response to carbohydrate stimulus | 0.000507 | 0.00269 | TH/PIK3CA/ADRA2A/OPRK1 | 4 |
| BP | GO:1904064 | positive regulation of cation transmembrane transport | 0.000507 | 0.00269 | DRD4/DRD1/ADRB2/OPRK1 | 4 |
| BP | GO:0008654 | phospholipid biosynthetic process | 0.000529 | 0.002799 | ACHE/PIK3CA/HTR2A/FGF2/HTR2C | 5 |
| BP | GO:0051384 | response to glucocorticoid | 0.000535 | 0.002821 | TNF/TH/BCHE/NR3C1 | 4 |
| BP | GO:0051091 | positive regulation of DNA-binding transcription factor activity | 0.000539 | 0.002836 | TNF/VEGFA/ESR1/PPARG/JAK2 | 5 |
| BP | GO:0032922 | circadian regulation of gene expression | 0.000542 | 0.002849 | DRD2/DRD3/HDAC1 | 3 |
| BP | GO:0045785 | positive regulation of cell adhesion | 0.000556 | 0.002913 | TNF/VEGFA/PIK3CA/IL2/KDR/JAK2 | 6 |
| BP | GO:0014074 | response to purine-containing compound | 0.000577 | 0.002987 | SLC6A4/REN/SLC6A3/PPARG | 4 |
| BP | GO:0014841 | skeletal muscle satellite cell proliferation | 0.000578 | 0.002987 | FGF2/JAK2 | 2 |
| BP | GO:0014857 | regulation of skeletal muscle cell proliferation | 0.000578 | 0.002987 | FGF2/JAK2 | 2 |
| BP | GO:0035815 | positive regulation of renal sodium excretion | 0.000578 | 0.002987 | DRD2/DRD3 | 2 |
| BP | GO:0097154 | GABAergic neuron differentiation | 0.000578 | 0.002987 | DRD2/DRD1 | 2 |
| BP | GO:1901386 | negative regulation of voltage-gated calcium channel activity | 0.000578 | 0.002987 | DRD2/DRD4 | 2 |
| BP | GO:1901617 | organic hydroxy compound biosynthetic process | 0.000587 | 0.003029 | TNF/TH/SLC6A3/FGF2/CYP19A1 | 5 |
| BP | GO:0048754 | branching morphogenesis of an epithelial tube | 0.000592 | 0.003041 | TNF/VEGFA/ESR1/FGF2 | 4 |
| BP | GO:0050777 | negative regulation of immune response | 0.000592 | 0.003041 | TNF/DRD2/IL2/PPARG | 4 |
| BP | GO:0045600 | positive regulation of fat cell differentiation | 0.000595 | 0.003045 | HTR2A/HTR2C/PPARG | 3 |
| BP | GO:1903672 | positive regulation of sprouting angiogenesis | 0.000595 | 0.003045 | VEGFA/FGF2/KDR | 3 |
| BP | GO:0050714 | positive regulation of protein secretion | 0.000607 | 0.003099 | TNF/DRD2/ACHE/IL2/JAK2 | 5 |
| BP | GO:0000187 | activation of MAPK activity | 0.000622 | 0.003154 | TNF/DRD4/ADRA2B/FGF2 | 4 |
| BP | GO:0001678 | cellular glucose homeostasis | 0.000622 | 0.003154 | TH/PIK3CA/ADRA2A/OPRK1 | 4 |
| BP | GO:1903169 | regulation of calcium ion transmembrane transport | 0.000622 | 0.003154 | DRD2/DRD4/ADRA2A/DRD1 | 4 |
| BP | GO:0045927 | positive regulation of growth | 0.000628 | 0.003178 | DRD2/VEGFA/SLC6A3/FGF2/IL2 | 5 |
| BP | GO:0030168 | platelet activation | 0.000637 | 0.003219 | PIK3CA/ADRA2A/ADRA2C/ADRA2B | 4 |
| BP | GO:0051926 | negative regulation of calcium ion transport | 0.000651 | 0.003282 | DRD2/DRD4/ADRA2A | 3 |
| BP | GO:0014856 | skeletal muscle cell proliferation | 0.000665 | 0.003302 | FGF2/JAK2 | 2 |
| BP | GO:0030730 | sequestering of triglyceride | 0.000665 | 0.003302 | TNF/PPARG | 2 |
| BP | GO:0042754 | negative regulation of circadian rhythm | 0.000665 | 0.003302 | DRD2/ADORA1 | 2 |
| BP | GO:0050961 | detection of temperature stimulus involved in sensory perception | 0.000665 | 0.003302 | HTR2A/ADORA1 | 2 |
| BP | GO:0050965 | detection of temperature stimulus involved in sensory perception of pain | 0.000665 | 0.003302 | HTR2A/ADORA1 | 2 |
| BP | GO:0051044 | positive regulation of membrane protein ectodomain proteolysis | 0.000665 | 0.003302 | TNF/ADRA2A | 2 |
| BP | GO:0051770 | positive regulation of nitric-oxide synthase biosynthetic process | 0.000665 | 0.003302 | KDR/JAK2 | 2 |
| BP | GO:0043542 | endothelial cell migration | 0.000671 | 0.003324 | VEGFA/PIK3CA/FGF2/PPARG/KDR | 5 |
| BP | GO:0034767 | positive regulation of ion transmembrane transport | 0.000686 | 0.003375 | DRD4/DRD1/ADRB2/OPRK1 | 4 |
| BP | GO:0043535 | regulation of blood vessel endothelial cell migration | 0.000686 | 0.003375 | VEGFA/FGF2/PPARG/KDR | 4 |
| BP | GO:1905952 | regulation of lipid localization | 0.000686 | 0.003375 | TNF/REN/PPARG/CYP19A1 | 4 |
| BP | GO:0050918 | positive chemotaxis | 0.000711 | 0.003491 | VEGFA/FGF2/KDR | 3 |
| BP | GO:0030522 | intracellular receptor signaling pathway | 0.000716 | 0.003511 | ESR1/NR3C1/PPARG/JAK2/HDAC1 | 5 |
| BP | GO:0009896 | positive regulation of catabolic process | 0.000734 | 0.003591 | TNF/HTR2A/ADRA2A/ADRB2/ADORA1/KDR | 6 |
| BP | GO:0042698 | ovulation cycle | 0.000742 | 0.003621 | OPRM1/ESR1/OPRK1 | 3 |
| BP | GO:0031958 | corticosteroid receptor signaling pathway | 0.000759 | 0.003674 | NR3C1/JAK2 | 2 |
| BP | GO:0044849 | estrous cycle | 0.000759 | 0.003674 | OPRM1/OPRK1 | 2 |
| BP | GO:0060965 | negative regulation of gene silencing by miRNA | 0.000759 | 0.003674 | ESR1/PPARG | 2 |
| BP | GO:0090036 | regulation of protein kinase C signaling | 0.000759 | 0.003674 | VEGFA/ADRA1A | 2 |
| BP | GO:0072091 | regulation of stem cell proliferation | 0.000774 | 0.003737 | DRD2/VEGFA/ACE | 3 |
| BP | GO:0051090 | regulation of DNA-binding transcription factor activity | 0.000799 | 0.003853 | TNF/VEGFA/ESR1/MAPK8/PPARG/JAK2 | 6 |
| BP | GO:0042531 | positive regulation of tyrosine phosphorylation of STAT protein | 0.000806 | 0.003879 | TNF/IL2/JAK2 | 3 |
| BP | GO:0050673 | epithelial cell proliferation | 0.000819 | 0.003931 | TNF/VEGFA/ESR1/FGF2/PPARG/KDR | 6 |
| BP | GO:0002683 | negative regulation of immune system process | 0.000828 | 0.00397 | TNF/DRD2/IL2/PPARG/ADORA1/CYP19A1 | 6 |
| BP | GO:0030540 | female genitalia development | 0.000859 | 0.004065 | ESR1/CYP19A1 | 2 |
| BP | GO:0048521 | negative regulation of behavior | 0.000859 | 0.004065 | DRD2/ADORA1 | 2 |
| BP | GO:0060080 | inhibitory postsynaptic potential | 0.000859 | 0.004065 | CHRNA4/DRD4 | 2 |
| BP | GO:0098884 | postsynaptic neurotransmitter receptor internalization | 0.000859 | 0.004065 | DRD4/DRD3 | 2 |
| BP | GO:0099509 | regulation of presynaptic cytosolic calcium ion concentration | 0.000859 | 0.004065 | HTR1B/ADORA1 | 2 |
| BP | GO:0140239 | postsynaptic endocytosis | 0.000859 | 0.004065 | DRD4/DRD3 | 2 |
| BP | GO:0043627 | response to estrogen | 0.000874 | 0.004121 | ESR1/OPRK1/PPARG | 3 |
| BP | GO:0045685 | regulation of glial cell differentiation | 0.000874 | 0.004121 | DRD3/PPARG/HDAC1 | 3 |
| BP | GO:0051302 | regulation of cell division | 0.000904 | 0.004254 | DRD2/VEGFA/DRD3/FGF2 | 4 |
| BP | GO:0014015 | positive regulation of gliogenesis | 0.00091 | 0.004271 | TNF/PPARG/HDAC1 | 3 |
| BP | GO:0021543 | pallium development | 0.000924 | 0.004314 | TH/DRD1/HTR5A/HDAC1 | 4 |
| BP | GO:0050728 | negative regulation of inflammatory response | 0.000924 | 0.004314 | IL2/PPARG/ADORA1/CYP19A1 | 4 |
| BP | GO:0051100 | negative regulation of binding | 0.000924 | 0.004314 | ACE/MAPK8/ADRB2/JAK2 | 4 |
| BP | GO:0021756 | striatum development | 0.000965 | 0.004454 | DRD2/DRD1 | 2 |
| BP | GO:0051023 | regulation of immunoglobulin secretion | 0.000965 | 0.004454 | TNF/IL2 | 2 |
| BP | GO:0060749 | mammary gland alveolus development | 0.000965 | 0.004454 | VEGFA/ESR1 | 2 |
| BP | GO:0061377 | mammary gland lobule development | 0.000965 | 0.004454 | VEGFA/ESR1 | 2 |
| BP | GO:1900221 | regulation of amyloid-beta clearance | 0.000965 | 0.004454 | TNF/HDAC1 | 2 |
| BP | GO:0016241 | regulation of macroautophagy | 0.000966 | 0.004454 | PIK3CA/MAPK8/ADRB2/KDR | 4 |
| BP | GO:0018105 | peptidyl-serine phosphorylation | 0.000993 | 0.004571 | TNF/VEGFA/PIK3CA/DRD1/MAPK8 | 5 |
| BP | GO:0008344 | adult locomotory behavior | 0.001021 | 0.004681 | DRD2/DRD4/DRD1 | 3 |
| BP | GO:0016575 | histone deacetylation | 0.001021 | 0.004681 | VEGFA/MAPK8/HDAC1 | 3 |
| BP | GO:0046488 | phosphatidylinositol metabolic process | 0.00103 | 0.004714 | PIK3CA/HTR2A/FGF2/HTR2C | 4 |
| BP | GO:0051897 | positive regulation of protein kinase B signaling | 0.001075 | 0.004822 | TNF/ESR1/PIK3CA/FGF2 | 4 |
| BP | GO:0001991 | regulation of systemic arterial blood pressure by circulatory renin-angiotensin | 0.001076 | 0.004822 | REN/ACE | 2 |
| BP | GO:0002544 | chronic inflammatory response | 0.001076 | 0.004822 | TNF/CYP19A1 | 2 |
| BP | GO:0007213 | G protein-coupled acetylcholine receptor signaling pathway | 0.001076 | 0.004822 | OPRM1/CHRM1 | 2 |
| BP | GO:0007625 | grooming behavior | 0.001076 | 0.004822 | DRD2/DRD1 | 2 |
| BP | GO:0010888 | negative regulation of lipid storage | 0.001076 | 0.004822 | TNF/PPARG | 2 |
| BP | GO:0016048 | detection of temperature stimulus | 0.001076 | 0.004822 | HTR2A/ADORA1 | 2 |
| BP | GO:0060004 | reflex | 0.001076 | 0.004822 | ADRA1A/DRD3 | 2 |
| BP | GO:0060149 | negative regulation of posttranscriptional gene silencing | 0.001076 | 0.004822 | ESR1/PPARG | 2 |
| BP | GO:0060967 | negative regulation of gene silencing by RNA | 0.001076 | 0.004822 | ESR1/PPARG | 2 |
| BP | GO:0071071 | regulation of phospholipid biosynthetic process | 0.001076 | 0.004822 | HTR2A/HTR2C | 2 |
| BP | GO:0006941 | striated muscle contraction | 0.001097 | 0.004907 | PIK3CA/ADRA1B/ADRA1A/ADORA1 | 4 |
| BP | GO:0043536 | positive regulation of blood vessel endothelial cell migration | 0.0011 | 0.004907 | VEGFA/FGF2/KDR | 3 |
| BP | GO:0009895 | negative regulation of catabolic process | 0.001133 | 0.005047 | TNF/PIK3CA/ADRA2A/ADRA1A/ADORA1 | 5 |
| BP | GO:0043534 | blood vessel endothelial cell migration | 0.001168 | 0.005182 | VEGFA/FGF2/PPARG/KDR | 4 |
| BP | GO:2001257 | regulation of cation channel activity | 0.001168 | 0.005182 | DRD2/OPRM1/DRD4/ADRB2 | 4 |
| BP | GO:0021766 | hippocampus development | 0.001182 | 0.005236 | DRD1/HTR5A/HDAC1 | 3 |
| BP | GO:0051767 | nitric-oxide synthase biosynthetic process | 0.001194 | 0.005268 | KDR/JAK2 | 2 |
| BP | GO:0051769 | regulation of nitric-oxide synthase biosynthetic process | 0.001194 | 0.005268 | KDR/JAK2 | 2 |
| BP | GO:0061138 | morphogenesis of a branching epithelium | 0.001216 | 0.005357 | TNF/VEGFA/ESR1/FGF2 | 4 |
| BP | GO:0055117 | regulation of cardiac muscle contraction | 0.001225 | 0.005374 | ADRA1B/ADRA1A/ADORA1 | 3 |
| BP | GO:0090049 | regulation of cell migration involved in sprouting angiogenesis | 0.001225 | 0.005374 | VEGFA/FGF2/KDR | 3 |
| BP | GO:0009416 | response to light stimulus | 0.001234 | 0.005406 | TH/DRD2/DRD1/MAPK8/DRD3 | 5 |
| BP | GO:0048639 | positive regulation of developmental growth | 0.001267 | 0.005535 | DRD2/VEGFA/SLC6A3/FGF2 | 4 |
| BP | GO:0042509 | regulation of tyrosine phosphorylation of STAT protein | 0.001268 | 0.005535 | TNF/IL2/JAK2 | 3 |
| BP | GO:0046889 | positive regulation of lipid biosynthetic process | 0.001313 | 0.005695 | TNF/HTR2A/HTR2C | 3 |
| BP | GO:0009713 | catechol-containing compound biosynthetic process | 0.001317 | 0.005695 | TH/SLC6A3 | 2 |
| BP | GO:0035162 | embryonic hemopoiesis | 0.001317 | 0.005695 | VEGFA/KDR | 2 |
| BP | GO:0042135 | neurotransmitter catabolic process | 0.001317 | 0.005695 | MAOA/ACHE | 2 |
| BP | GO:0042423 | catecholamine biosynthetic process | 0.001317 | 0.005695 | TH/SLC6A3 | 2 |
| BP | GO:0006650 | glycerophospholipid metabolic process | 0.001324 | 0.005712 | ACHE/PIK3CA/HTR2A/FGF2/HTR2C | 5 |
| BP | GO:0048167 | regulation of synaptic plasticity | 0.001344 | 0.00579 | DRD2/DRD1/DRD5/ADORA1 | 4 |
| BP | GO:0018209 | peptidyl-serine modification | 0.00138 | 0.005931 | TNF/VEGFA/PIK3CA/DRD1/MAPK8 | 5 |
| BP | GO:0006919 | activation of cysteine-type endopeptidase activity involved in apoptotic process | 0.001405 | 0.006008 | TNF/PPARG/JAK2 | 3 |
| BP | GO:0007260 | tyrosine phosphorylation of STAT protein | 0.001405 | 0.006008 | TNF/IL2/JAK2 | 3 |
| BP | GO:0060291 | long-term synaptic potentiation | 0.001405 | 0.006008 | DRD2/DRD1/ADORA1 | 3 |
| BP | GO:0002052 | positive regulation of neuroblast proliferation | 0.001447 | 0.00613 | DRD2/VEGFA | 2 |
| BP | GO:0010155 | regulation of proton transport | 0.001447 | 0.00613 | DRD4/DRD3 | 2 |
| BP | GO:0031281 | positive regulation of cyclase activity | 0.001447 | 0.00613 | MAPK8/DRD5 | 2 |
| BP | GO:0048305 | immunoglobulin secretion | 0.001447 | 0.00613 | TNF/IL2 | 2 |
| BP | GO:0060065 | uterus development | 0.001447 | 0.00613 | ESR1/CYP19A1 | 2 |
| BP | GO:0007565 | female pregnancy | 0.001481 | 0.006253 | ESR1/ADRA2C/ADRA2B/MMP2 | 4 |
| BP | GO:1901888 | regulation of cell junction assembly | 0.001481 | 0.006253 | TNF/VEGFA/ACE/KDR | 4 |
| BP | GO:0010506 | regulation of autophagy | 0.001497 | 0.006293 | PIK3CA/MAPK8/ADRA1A/ADRB2/KDR | 5 |
| BP | GO:1903725 | regulation of phospholipid metabolic process | 0.001501 | 0.006293 | HTR2A/FGF2/HTR2C | 3 |
| BP | GO:1904035 | regulation of epithelial cell apoptotic process | 0.001501 | 0.006293 | TNF/KDR/JAK2 | 3 |
| BP | GO:2000177 | regulation of neural precursor cell proliferation | 0.001501 | 0.006293 | SLC6A4/DRD2/VEGFA | 3 |
| BP | GO:0046427 | positive regulation of receptor signaling pathway via JAK-STAT | 0.001551 | 0.006478 | TNF/IL2/JAK2 | 3 |
| BP | GO:1901019 | regulation of calcium ion transmembrane transporter activity | 0.001551 | 0.006478 | DRD2/DRD4/ADRA2A | 3 |
| BP | GO:0032098 | regulation of appetite | 0.001582 | 0.006549 | OPRM1/HTR2C | 2 |
| BP | GO:0048714 | positive regulation of oligodendrocyte differentiation | 0.001582 | 0.006549 | PPARG/HDAC1 | 2 |
| BP | GO:0051043 | regulation of membrane protein ectodomain proteolysis | 0.001582 | 0.006549 | TNF/ADRA2A | 2 |
| BP | GO:0062149 | detection of stimulus involved in sensory perception of pain | 0.001582 | 0.006549 | HTR2A/ADORA1 | 2 |
| BP | GO:0097164 | ammonium ion metabolic process | 0.001582 | 0.006549 | HTR1A/BCHE | 2 |
| BP | GO:0002685 | regulation of leukocyte migration | 0.001598 | 0.006602 | TNF/VEGFA/ADORA1/CYP19A1 | 4 |
| BP | GO:0015711 | organic anion transport | 0.001609 | 0.006636 | DRD2/ACE/DRD4/DRD3/PPARG/ADORA1 | 6 |
| BP | GO:0030336 | negative regulation of cell migration | 0.001621 | 0.006674 | DRD2/FGF2/PPARG/ADORA1/CYP19A1 | 5 |
| BP | GO:0009749 | response to glucose | 0.001628 | 0.006691 | TH/PIK3CA/ADRA2A/OPRK1 | 4 |
| BP | GO:0045833 | negative regulation of lipid metabolic process | 0.001706 | 0.006988 | TNF/ADRA2A/ADORA1 | 3 |
| BP | GO:1904894 | positive regulation of receptor signaling pathway via STAT | 0.001706 | 0.006988 | TNF/IL2/JAK2 | 3 |
| BP | GO:0002092 | positive regulation of receptor internalization | 0.001723 | 0.007009 | DRD2/VEGFA | 2 |
| BP | GO:0021544 | subpallium development | 0.001723 | 0.007009 | DRD2/DRD1 | 2 |
| BP | GO:0021895 | cerebral cortex neuron differentiation | 0.001723 | 0.007009 | DRD2/DRD1 | 2 |
| BP | GO:0099590 | neurotransmitter receptor internalization | 0.001723 | 0.007009 | DRD4/DRD3 | 2 |
| BP | GO:0006476 | protein deacetylation | 0.00176 | 0.007145 | VEGFA/MAPK8/HDAC1 | 3 |
| BP | GO:0009746 | response to hexose | 0.001784 | 0.007231 | TH/PIK3CA/ADRA2A/OPRK1 | 4 |
| BP | GO:0034764 | positive regulation of transmembrane transport | 0.001849 | 0.007483 | DRD4/DRD1/ADRB2/OPRK1 | 4 |
| BP | GO:0003071 | renal system process involved in regulation of systemic arterial blood pressure | 0.00187 | 0.007489 | REN/ADORA1 | 2 |
| BP | GO:0014829 | vascular smooth muscle contraction | 0.00187 | 0.007489 | HTR2A/ADRA2B | 2 |
| BP | GO:0050927 | positive regulation of positive chemotaxis | 0.00187 | 0.007489 | VEGFA/KDR | 2 |
| BP | GO:0051894 | positive regulation of focal adhesion assembly | 0.00187 | 0.007489 | VEGFA/KDR | 2 |
| BP | GO:0060330 | regulation of response to interferon-gamma | 0.00187 | 0.007489 | PPARG/JAK2 | 2 |
| BP | GO:0060334 | regulation of interferon-gamma-mediated signaling pathway | 0.00187 | 0.007489 | PPARG/JAK2 | 2 |
| BP | GO:0008585 | female gonad development | 0.001927 | 0.007692 | VEGFA/ESR1/CYP19A1 | 3 |
| BP | GO:0048010 | vascular endothelial growth factor receptor signaling pathway | 0.001927 | 0.007692 | VEGFA/PIK3CA/KDR | 3 |
| BP | GO:0034284 | response to monosaccharide | 0.00195 | 0.007771 | TH/PIK3CA/ADRA2A/OPRK1 | 4 |
| BP | GO:2000146 | negative regulation of cell motility | 0.001965 | 0.007815 | DRD2/FGF2/PPARG/ADORA1/CYP19A1 | 5 |
| BP | GO:0006942 | regulation of striated muscle contraction | 0.001985 | 0.007869 | ADRA1B/ADRA1A/ADORA1 | 3 |
| BP | GO:0048709 | oligodendrocyte differentiation | 0.001985 | 0.007869 | DRD3/PPARG/HDAC1 | 3 |
| BP | GO:0062197 | cellular response to chemical stress | 0.001989 | 0.007873 | TNF/MAPK8/PPARG/JAK2/MMP2 | 5 |
| BP | GO:0035303 | regulation of dephosphorylation | 0.00202 | 0.007924 | TNF/DRD2/ADORA1/JAK2 | 4 |
| BP | GO:0010460 | positive regulation of heart rate | 0.002022 | 0.007924 | ADRA1A/ADRB1 | 2 |
| BP | GO:0031954 | positive regulation of protein autophosphorylation | 0.002022 | 0.007924 | VEGFA/ACE | 2 |
| BP | GO:0035640 | exploration behavior | 0.002022 | 0.007924 | CHRNA4/HTR1A | 2 |
| BP | GO:0050926 | regulation of positive chemotaxis | 0.002022 | 0.007924 | VEGFA/KDR | 2 |
| BP | GO:0150117 | positive regulation of cell-substrate junction organization | 0.002022 | 0.007924 | VEGFA/KDR | 2 |
| BP | GO:0010631 | epithelial cell migration | 0.002039 | 0.007968 | VEGFA/PIK3CA/FGF2/PPARG/KDR | 5 |
| BP | GO:0002042 | cell migration involved in sprouting angiogenesis | 0.002044 | 0.007968 | VEGFA/FGF2/KDR | 3 |
| BP | GO:0045807 | positive regulation of endocytosis | 0.002044 | 0.007968 | DRD2/VEGFA/PPARG | 3 |
| BP | GO:0009612 | response to mechanical stimulus | 0.002055 | 0.007999 | DRD2/HTR2A/MAPK8/PPARG | 4 |
| BP | GO:0090132 | epithelium migration | 0.002116 | 0.008222 | VEGFA/PIK3CA/FGF2/PPARG/KDR | 5 |
| BP | GO:0002021 | response to dietary excess | 0.002181 | 0.008403 | ADRB2/ADRB1 | 2 |
| BP | GO:0002026 | regulation of the force of heart contraction | 0.002181 | 0.008403 | ADRA1A/ADRB1 | 2 |
| BP | GO:0007176 | regulation of epidermal growth factor-activated receptor activity | 0.002181 | 0.008403 | ADRA2A/ADORA1 | 2 |
| BP | GO:0009651 | response to salt stress | 0.002181 | 0.008403 | TNF/TH | 2 |
| BP | GO:2001039 | negative regulation of cellular response to drug | 0.002181 | 0.008403 | DRD2/DRD3 | 2 |
| BP | GO:0002027 | regulation of heart rate | 0.002228 | 0.008542 | DRD2/ADRA1A/ADRB1 | 3 |
| BP | GO:0046545 | development of primary female sexual characteristics | 0.002228 | 0.008542 | VEGFA/ESR1/CYP19A1 | 3 |
| BP | GO:1903426 | regulation of reactive oxygen species biosynthetic process | 0.002228 | 0.008542 | TNF/SLC18A2/JAK2 | 3 |
| BP | GO:0043281 | regulation of cysteine-type endopeptidase activity involved in apoptotic process | 0.002238 | 0.00857 | TNF/VEGFA/PPARG/JAK2 | 4 |
| BP | GO:0090130 | tissue migration | 0.002276 | 0.008698 | VEGFA/PIK3CA/FGF2/PPARG/KDR | 5 |
| BP | GO:0001823 | mesonephros development | 0.002291 | 0.008742 | VEGFA/REN/FGF2 | 3 |
| BP | GO:0031331 | positive regulation of cellular catabolic process | 0.002303 | 0.008775 | TNF/HTR2A/ADRA2A/ADRB2/KDR | 5 |
| BP | GO:0008406 | gonad development | 0.002315 | 0.00879 | VEGFA/ESR1/REN/CYP19A1 | 4 |
| BP | GO:0050920 | regulation of chemotaxis | 0.002315 | 0.00879 | VEGFA/FGF2/KDR/CYP19A1 | 4 |
| BP | GO:0008209 | androgen metabolic process | 0.002344 | 0.008887 | ESR1/CYP19A1 | 2 |
| BP | GO:0006814 | sodium ion transport | 0.002354 | 0.008887 | DRD2/DRD4/ADRB2/DRD3 | 4 |
| BP | GO:0032091 | negative regulation of protein binding | 0.002355 | 0.008887 | ACE/MAPK8/ADRB2 | 3 |
| BP | GO:0035601 | protein deacylation | 0.002355 | 0.008887 | VEGFA/MAPK8/HDAC1 | 3 |
| BP | GO:0098732 | macromolecule deacylation | 0.002421 | 0.00912 | VEGFA/MAPK8/HDAC1 | 3 |
| BP | GO:0033138 | positive regulation of peptidyl-serine phosphorylation | 0.002488 | 0.009356 | TNF/VEGFA/PIK3CA | 3 |
| BP | GO:0007271 | synaptic transmission, cholinergic | 0.002514 | 0.009394 | ACHE/CHRNA4 | 2 |
| BP | GO:0007628 | adult walking behavior | 0.002514 | 0.009394 | DRD2/DRD1 | 2 |
| BP | GO:0031063 | regulation of histone deacetylation | 0.002514 | 0.009394 | VEGFA/MAPK8 | 2 |
| BP | GO:0070528 | protein kinase C signaling | 0.002514 | 0.009394 | VEGFA/ADRA1A | 2 |
| BP | GO:0045137 | development of primary sexual characteristics | 0.002555 | 0.009531 | VEGFA/ESR1/REN/CYP19A1 | 4 |
| BP | GO:0051098 | regulation of binding | 0.002622 | 0.009765 | ACE/MAPK8/ADRB2/PPARG/JAK2 | 5 |
| BP | GO:0007274 | neuromuscular synaptic transmission | 0.002689 | 0.009985 | CHRNA4/CHRM1 | 2 |
| BP | GO:1901889 | negative regulation of cell junction assembly | 0.002689 | 0.009985 | TNF/ACE | 2 |
| BP | GO:0050678 | regulation of epithelial cell proliferation | 0.002776 | 0.010293 | TNF/VEGFA/FGF2/PPARG/KDR | 5 |
| BP | GO:0010594 | regulation of endothelial cell migration | 0.002811 | 0.010406 | VEGFA/FGF2/PPARG/KDR | 4 |
| BP | GO:0009743 | response to carbohydrate | 0.002856 | 0.010516 | TH/PIK3CA/ADRA2A/OPRK1 | 4 |
| BP | GO:0007202 | activation of phospholipase C activity | 0.00287 | 0.010516 | HTR2A/ADRA1A | 2 |
| BP | GO:0040018 | positive regulation of multicellular organism growth | 0.00287 | 0.010516 | DRD2/SLC6A3 | 2 |
| BP | GO:0045742 | positive regulation of epidermal growth factor receptor signaling pathway | 0.00287 | 0.010516 | ADRA2A/ADORA1 | 2 |
| BP | GO:0051482 | positive regulation of cytosolic calcium ion concentration involved in phospholipase C-activating G protein-coupled signaling pathway | 0.00287 | 0.010516 | DRD1/HTR2C | 2 |
| BP | GO:0090659 | walking behavior | 0.00287 | 0.010516 | DRD2/DRD1 | 2 |
| BP | GO:0040013 | negative regulation of locomotion | 0.002872 | 0.010516 | DRD2/FGF2/PPARG/ADORA1/CYP19A1 | 5 |
| BP | GO:1904019 | epithelial cell apoptotic process | 0.002912 | 0.010645 | TNF/KDR/JAK2 | 3 |
| BP | GO:0071695 | anatomical structure maturation | 0.002946 | 0.010751 | VEGFA/REN/PPARG/MMP2 | 4 |
| BP | GO:0051271 | negative regulation of cellular component movement | 0.002971 | 0.010825 | DRD2/FGF2/PPARG/ADORA1/CYP19A1 | 5 |
| BP | GO:0001938 | positive regulation of endothelial cell proliferation | 0.002987 | 0.010867 | VEGFA/FGF2/KDR | 3 |
| BP | GO:0051385 | response to mineralocorticoid | 0.003056 | 0.011085 | TH/HTR1B | 2 |
| BP | GO:1900745 | positive regulation of p38MAPK cascade | 0.003056 | 0.011085 | VEGFA/OPRK1 | 2 |
| BP | GO:0048640 | negative regulation of developmental growth | 0.003063 | 0.011092 | SLC6A4/ADRB2/ADRB1 | 3 |
| BP | GO:0046777 | protein autophosphorylation | 0.003085 | 0.011154 | VEGFA/ACE/KDR/JAK2 | 4 |
| BP | GO:0045446 | endothelial cell differentiation | 0.00314 | 0.011336 | TNF/VEGFA/KDR | 3 |
| BP | GO:0046660 | female sex differentiation | 0.003218 | 0.011601 | VEGFA/ESR1/CYP19A1 | 3 |
| BP | GO:0010039 | response to iron ion | 0.003248 | 0.011654 | DRD2/SLC6A3 | 2 |
| BP | GO:1901186 | positive regulation of ERBB signaling pathway | 0.003248 | 0.011654 | ADRA2A/ADORA1 | 2 |
| BP | GO:1902692 | regulation of neuroblast proliferation | 0.003248 | 0.011654 | DRD2/VEGFA | 2 |
| BP | GO:2000116 | regulation of cysteine-type endopeptidase activity | 0.003277 | 0.011741 | TNF/VEGFA/PPARG/JAK2 | 4 |
| BP | GO:0043666 | regulation of phosphoprotein phosphatase activity | 0.003379 | 0.012086 | TNF/DRD2/JAK2 | 3 |
| BP | GO:0016486 | peptide hormone processing | 0.003445 | 0.012269 | REN/ACE | 2 |
| BP | GO:0060969 | negative regulation of gene silencing | 0.003445 | 0.012269 | ESR1/PPARG | 2 |
| BP | GO:0140448 | signaling receptor ligand precursor processing | 0.003445 | 0.012269 | REN/ACE | 2 |
| BP | GO:0007173 | epidermal growth factor receptor signaling pathway | 0.003544 | 0.012599 | PIK3CA/ADRA2A/ADORA1 | 3 |
| BP | GO:0022407 | regulation of cell-cell adhesion | 0.003613 | 0.012828 | TNF/VEGFA/PIK3CA/IL2/JAK2 | 5 |
| BP | GO:0042462 | eye photoreceptor cell development | 0.003648 | 0.012873 | TH/VEGFA | 2 |
| BP | GO:0060251 | regulation of glial cell proliferation | 0.003648 | 0.012873 | TNF/CHRM1 | 2 |
| BP | GO:1901385 | regulation of voltage-gated calcium channel activity | 0.003648 | 0.012873 | DRD2/DRD4 | 2 |
| BP | GO:2001024 | negative regulation of response to drug | 0.003648 | 0.012873 | DRD2/DRD3 | 2 |
| BP | GO:0010811 | positive regulation of cell-substrate adhesion | 0.003714 | 0.013084 | VEGFA/KDR/JAK2 | 3 |
| BP | GO:0045926 | negative regulation of growth | 0.003793 | 0.013345 | SLC6A4/ADRB2/PPARG/ADRB1 | 4 |
| BP | GO:1903409 | reactive oxygen species biosynthetic process | 0.0038 | 0.01335 | TNF/SLC18A2/JAK2 | 3 |
| BP | GO:0030224 | monocyte differentiation | 0.003857 | 0.013487 | VEGFA/PPARG | 2 |
| BP | GO:0097242 | amyloid-beta clearance | 0.003857 | 0.013487 | TNF/HDAC1 | 2 |
| BP | GO:1903131 | mononuclear cell differentiation | 0.003857 | 0.013487 | VEGFA/PPARG | 2 |
| BP | GO:0051101 | regulation of DNA binding | 0.003978 | 0.013889 | MAPK8/PPARG/JAK2 | 3 |
| BP | GO:0032368 | regulation of lipid transport | 0.004068 | 0.014111 | REN/PPARG/CYP19A1 | 3 |
| BP | GO:0002714 | positive regulation of B cell mediated immunity | 0.00407 | 0.014111 | TNF/IL2 | 2 |
| BP | GO:0002891 | positive regulation of immunoglobulin mediated immune response | 0.00407 | 0.014111 | TNF/IL2 | 2 |
| BP | GO:0043243 | positive regulation of protein-containing complex disassembly | 0.00407 | 0.014111 | TNF/ADRB2 | 2 |
| BP | GO:0051924 | regulation of calcium ion transport | 0.004071 | 0.014111 | DRD2/DRD4/ADRA2A/DRD1 | 4 |
| BP | GO:0046486 | glycerolipid metabolic process | 0.004094 | 0.014169 | ACHE/PIK3CA/HTR2A/FGF2/HTR2C | 5 |
| BP | GO:0030099 | myeloid cell differentiation | 0.004178 | 0.01444 | TNF/VEGFA/PPARG/JAK2/HDAC1 | 5 |
| BP | GO:0046887 | positive regulation of hormone secretion | 0.004253 | 0.014676 | DRD2/JAK2/CYP19A1 | 3 |
| BP | GO:0016572 | histone phosphorylation | 0.004289 | 0.014758 | DRD1/JAK2 | 2 |
| BP | GO:0046676 | negative regulation of insulin secretion | 0.004289 | 0.014758 | DRD2/ADRA2A | 2 |
| BP | GO:0010595 | positive regulation of endothelial cell migration | 0.004347 | 0.014935 | VEGFA/FGF2/KDR | 3 |
| BP | GO:2000648 | positive regulation of stem cell proliferation | 0.004514 | 0.015485 | DRD2/VEGFA | 2 |
| BP | GO:0052548 | regulation of endopeptidase activity | 0.004574 | 0.015667 | TNF/VEGFA/PPARG/JAK2/HDAC1 | 5 |
| BP | GO:0003158 | endothelium development | 0.004737 | 0.016155 | TNF/VEGFA/KDR | 3 |
| BP | GO:0043280 | positive regulation of cysteine-type endopeptidase activity involved in apoptotic process | 0.004737 | 0.016155 | TNF/PPARG/JAK2 | 3 |
| BP | GO:0032467 | positive regulation of cytokinesis | 0.004744 | 0.016155 | DRD2/DRD3 | 2 |
| BP | GO:0045823 | positive regulation of heart contraction | 0.004744 | 0.016155 | ADRA1A/ADRB1 | 2 |
| BP | GO:0006644 | phospholipid metabolic process | 0.004804 | 0.016337 | ACHE/PIK3CA/HTR2A/FGF2/HTR2C | 5 |
| BP | GO:0001508 | action potential | 0.004838 | 0.016427 | CHRNA4/DRD1/ADRA1A | 3 |
| BP | GO:0048608 | reproductive structure development | 0.004851 | 0.01645 | VEGFA/ESR1/REN/PPARG/CYP19A1 | 5 |
| BP | GO:0046683 | response to organophosphorus | 0.00494 | 0.016725 | SLC6A4/REN/SLC6A3 | 3 |
| BP | GO:0050873 | brown fat cell differentiation | 0.004979 | 0.016785 | ADRB2/ADRB1 | 2 |
| BP | GO:0061028 | establishment of endothelial barrier | 0.004979 | 0.016785 | TNF/VEGFA | 2 |
| BP | GO:0140353 | lipid export from cell | 0.004979 | 0.016785 | REN/CYP19A1 | 2 |
| BP | GO:0061458 | reproductive system development | 0.004995 | 0.016814 | VEGFA/ESR1/REN/PPARG/CYP19A1 | 5 |
| BP | GO:0050921 | positive regulation of chemotaxis | 0.005043 | 0.016952 | VEGFA/FGF2/KDR | 3 |
| BP | GO:0007548 | sex differentiation | 0.005052 | 0.016959 | VEGFA/ESR1/REN/CYP19A1 | 4 |
| BP | GO:0043434 | response to peptide hormone | 0.005092 | 0.017068 | TH/PIK3CA/OPRK1/PPARG/JAK2 | 5 |
| BP | GO:0006509 | membrane protein ectodomain proteolysis | 0.005219 | 0.017445 | TNF/ADRA2A | 2 |
| BP | GO:0021983 | pituitary gland development | 0.005219 | 0.017445 | DRD2/SLC6A3 | 2 |
| BP | GO:0060249 | anatomical structure homeostasis | 0.00524 | 0.017484 | VEGFA/SLC6A2/ADRB2/HTR1B/ADORA1 | 5 |
| BP | GO:0046425 | regulation of receptor signaling pathway via JAK-STAT | 0.005253 | 0.017484 | TNF/IL2/JAK2 | 3 |
| BP | GO:1903670 | regulation of sprouting angiogenesis | 0.005253 | 0.017484 | VEGFA/FGF2/KDR | 3 |
| BP | GO:0031279 | regulation of cyclase activity | 0.005465 | 0.018073 | MAPK8/DRD5 | 2 |
| BP | GO:0045429 | positive regulation of nitric oxide biosynthetic process | 0.005465 | 0.018073 | TNF/JAK2 | 2 |
| BP | GO:0045687 | positive regulation of glial cell differentiation | 0.005465 | 0.018073 | PPARG/HDAC1 | 2 |
| BP | GO:2001038 | regulation of cellular response to drug | 0.005465 | 0.018073 | DRD2/DRD3 | 2 |
| BP | GO:0033135 | regulation of peptidyl-serine phosphorylation | 0.005469 | 0.018073 | TNF/VEGFA/PIK3CA | 3 |
| BP | GO:0042737 | drug catabolic process | 0.005579 | 0.01841 | MAOA/ACHE/SLC6A3 | 3 |
| BP | GO:0001822 | kidney development | 0.005597 | 0.018446 | VEGFA/REN/ACE/FGF2 | 4 |
| BP | GO:0062013 | positive regulation of small molecule metabolic process | 0.00569 | 0.0186 | TNF/HTR2A/PPARG | 3 |
| BP | GO:0014002 | astrocyte development | 0.005716 | 0.0186 | TNF/DRD1 | 2 |
| BP | GO:0035307 | positive regulation of protein dephosphorylation | 0.005716 | 0.0186 | ADORA1/JAK2 | 2 |
| BP | GO:0038084 | vascular endothelial growth factor signaling pathway | 0.005716 | 0.0186 | VEGFA/KDR | 2 |
| BP | GO:0044060 | regulation of endocrine process | 0.005716 | 0.0186 | REN/OPRK1 | 2 |
| BP | GO:0045601 | regulation of endothelial cell differentiation | 0.005716 | 0.0186 | TNF/VEGFA | 2 |
| BP | GO:0046189 | phenol-containing compound biosynthetic process | 0.005716 | 0.0186 | TH/SLC6A3 | 2 |
| BP | GO:0090311 | regulation of protein deacetylation | 0.005716 | 0.0186 | VEGFA/MAPK8 | 2 |
| BP | GO:1904407 | positive regulation of nitric oxide metabolic process | 0.005716 | 0.0186 | TNF/JAK2 | 2 |
| BP | GO:0038127 | ERBB signaling pathway | 0.005802 | 0.018855 | PIK3CA/ADRA2A/ADORA1 | 3 |
| BP | GO:0006979 | response to oxidative stress | 0.005865 | 0.019032 | TNF/CHRNA4/MAPK8/JAK2/MMP2 | 5 |
| BP | GO:0052547 | regulation of peptidase activity | 0.005919 | 0.019181 | TNF/VEGFA/PPARG/JAK2/HDAC1 | 5 |
| BP | GO:0090278 | negative regulation of peptide hormone secretion | 0.005972 | 0.019273 | DRD2/ADRA2A | 2 |
| BP | GO:0099637 | neurotransmitter receptor transport | 0.005972 | 0.019273 | DRD4/DRD3 | 2 |
| BP | GO:1900271 | regulation of long-term synaptic potentiation | 0.005972 | 0.019273 | DRD2/ADORA1 | 2 |
| BP | GO:0016570 | histone modification | 0.006029 | 0.019385 | VEGFA/DRD1/MAPK8/JAK2/HDAC1 | 5 |
| BP | GO:0021700 | developmental maturation | 0.006031 | 0.019385 | VEGFA/REN/PPARG/MMP2 | 4 |
| BP | GO:0072593 | reactive oxygen species metabolic process | 0.006031 | 0.019385 | TNF/SLC18A2/DRD5/JAK2 | 4 |
| BP | GO:0051403 | stress-activated MAPK cascade | 0.006181 | 0.019838 | TNF/VEGFA/MAPK8/OPRK1 | 4 |
| BP | GO:0001754 | eye photoreceptor cell differentiation | 0.006233 | 0.019924 | TH/VEGFA | 2 |
| BP | GO:0002437 | inflammatory response to antigenic stimulus | 0.006233 | 0.019924 | TNF/OPRM1 | 2 |
| BP | GO:0010883 | regulation of lipid storage | 0.006233 | 0.019924 | TNF/PPARG | 2 |
| BP | GO:1904892 | regulation of receptor signaling pathway via STAT | 0.006265 | 0.019999 | TNF/IL2/JAK2 | 3 |
| BP | GO:0001667 | ameboidal-type cell migration | 0.006482 | 0.020551 | VEGFA/PIK3CA/FGF2/PPARG/KDR | 5 |
| BP | GO:0031952 | regulation of protein autophosphorylation | 0.0065 | 0.020551 | VEGFA/ACE | 2 |
| BP | GO:0042461 | photoreceptor cell development | 0.0065 | 0.020551 | TH/VEGFA | 2 |
| BP | GO:0048806 | genitalia development | 0.0065 | 0.020551 | ESR1/CYP19A1 | 2 |
| BP | GO:0060986 | endocrine hormone secretion | 0.0065 | 0.020551 | REN/OPRK1 | 2 |
| BP | GO:0061614 | pri-miRNA transcription by RNA polymerase II | 0.0065 | 0.020551 | NR3C1/PPARG | 2 |
| BP | GO:1900744 | regulation of p38MAPK cascade | 0.0065 | 0.020551 | VEGFA/OPRK1 | 2 |
| BP | GO:0010632 | regulation of epithelial cell migration | 0.006566 | 0.020732 | VEGFA/FGF2/PPARG/KDR | 4 |
| BP | GO:0001819 | positive regulation of cytokine production | 0.006599 | 0.020808 | TNF/DRD2/ADRA2A/IL2/JAK2 | 5 |
| BP | GO:2001056 | positive regulation of cysteine-type endopeptidase activity | 0.006626 | 0.020868 | TNF/PPARG/JAK2 | 3 |
| BP | GO:0072001 | renal system development | 0.006724 | 0.021097 | VEGFA/REN/ACE/FGF2 | 4 |
| BP | GO:0061351 | neural precursor cell proliferation | 0.00675 | 0.021097 | SLC6A4/DRD2/VEGFA | 3 |
| BP | GO:0001774 | microglial cell activation | 0.006771 | 0.021097 | TNF/JAK2 | 2 |
| BP | GO:0002269 | leukocyte activation involved in inflammatory response | 0.006771 | 0.021097 | TNF/JAK2 | 2 |
| BP | GO:0007595 | lactation | 0.006771 | 0.021097 | VEGFA/SLC6A3 | 2 |
| BP | GO:0030850 | prostate gland development | 0.006771 | 0.021097 | ESR1/CYP19A1 | 2 |
| BP | GO:0101023 | vascular endothelial cell proliferation | 0.006771 | 0.021097 | FGF2/PPARG | 2 |
| BP | GO:1905562 | regulation of vascular endothelial cell proliferation | 0.006771 | 0.021097 | FGF2/PPARG | 2 |
| BP | GO:0016236 | macroautophagy | 0.006884 | 0.021421 | PIK3CA/MAPK8/ADRB2/KDR | 4 |
| BP | GO:0002686 | negative regulation of leukocyte migration | 0.007048 | 0.021871 | ADORA1/CYP19A1 | 2 |
| BP | GO:0043124 | negative regulation of I-kappaB kinase/NF-kappaB signaling | 0.007048 | 0.021871 | ESR1/HDAC1 | 2 |
| BP | GO:0050729 | positive regulation of inflammatory response | 0.007127 | 0.022089 | TNF/IL2/JAK2 | 3 |
| BP | GO:0016569 | covalent chromatin modification | 0.007206 | 0.022302 | VEGFA/DRD1/MAPK8/JAK2/HDAC1 | 5 |
| BP | GO:0055067 | monovalent inorganic cation homeostasis | 0.007256 | 0.022428 | DRD2/DRD3/ADORA1 | 3 |
| BP | GO:0014009 | glial cell proliferation | 0.007329 | 0.022626 | TNF/CHRM1 | 2 |
| BP | GO:0034599 | cellular response to oxidative stress | 0.007467 | 0.02302 | TNF/MAPK8/JAK2/MMP2 | 4 |
| BP | GO:0001954 | positive regulation of cell-matrix adhesion | 0.007616 | 0.023326 | VEGFA/KDR | 2 |
| BP | GO:0002712 | regulation of B cell mediated immunity | 0.007616 | 0.023326 | TNF/IL2 | 2 |
| BP | GO:0002889 | regulation of immunoglobulin mediated immune response | 0.007616 | 0.023326 | TNF/IL2 | 2 |
| BP | GO:0048260 | positive regulation of receptor-mediated endocytosis | 0.007616 | 0.023326 | DRD2/VEGFA | 2 |
| BP | GO:2001258 | negative regulation of cation channel activity | 0.007616 | 0.023326 | DRD2/DRD4 | 2 |
| BP | GO:0060326 | cell chemotaxis | 0.007639 | 0.023367 | VEGFA/FGF2/KDR/CYP19A1 | 4 |
| BP | GO:0001764 | neuron migration | 0.00765 | 0.023369 | DRD2/VEGFA/DRD1 | 3 |
| BP | GO:2000179 | positive regulation of neural precursor cell proliferation | 0.007908 | 0.024094 | DRD2/VEGFA | 2 |
| BP | GO:2000649 | regulation of sodium ion transmembrane transporter activity | 0.007908 | 0.024094 | DRD4/DRD3 | 2 |
| BP | GO:0007259 | receptor signaling pathway via JAK-STAT | 0.00792 | 0.024099 | TNF/IL2/JAK2 | 3 |
| BP | GO:0000186 | activation of MAPKK activity | 0.008205 | 0.024869 | ADORA1/JAK2 | 2 |
| BP | GO:0022029 | telencephalon cell migration | 0.008205 | 0.024869 | DRD2/DRD1 | 2 |
| BP | GO:0038066 | p38MAPK cascade | 0.008205 | 0.024869 | VEGFA/OPRK1 | 2 |
| BP | GO:1901214 | regulation of neuron death | 0.008447 | 0.02557 | TNF/PIK3CA/ADORA1/JAK2 | 4 |
| BP | GO:1904645 | response to amyloid-beta | 0.008506 | 0.025717 | ADRB2/MMP2 | 2 |
| BP | GO:0031098 | stress-activated protein kinase signaling cascade | 0.008634 | 0.026068 | TNF/VEGFA/MAPK8/OPRK1 | 4 |
| BP | GO:0001541 | ovarian follicle development | 0.008813 | 0.026575 | VEGFA/ESR1 | 2 |
| BP | GO:0050900 | leukocyte migration | 0.008893 | 0.026782 | TNF/VEGFA/PIK3CA/ADORA1/CYP19A1 | 5 |
| BP | GO:0043010 | camera-type eye development | 0.009015 | 0.027116 | TH/VEGFA/ACHE/HDAC1 | 4 |
| BP | GO:0021885 | forebrain cell migration | 0.009124 | 0.027409 | DRD2/DRD1 | 2 |
| BP | GO:0034614 | cellular response to reactive oxygen species | 0.009203 | 0.027561 | TNF/MAPK8/MMP2 | 3 |
| BP | GO:0006470 | protein dephosphorylation | 0.00921 | 0.027561 | TNF/DRD2/ADORA1/JAK2 | 4 |
| BP | GO:0007568 | aging | 0.00921 | 0.027561 | TH/HTR2A/SLC6A3/ADRA1A | 4 |
| BP | GO:0060562 | epithelial tube morphogenesis | 0.009309 | 0.02782 | TNF/VEGFA/ESR1/FGF2 | 4 |
| BP | GO:0097696 | receptor signaling pathway via STAT | 0.009352 | 0.027914 | TNF/IL2/JAK2 | 3 |
| BP | GO:0048008 | platelet-derived growth factor receptor signaling pathway | 0.009441 | 0.028107 | VEGFA/JAK2 | 2 |
| BP | GO:1903428 | positive regulation of reactive oxygen species biosynthetic process | 0.009441 | 0.028107 | TNF/JAK2 | 2 |
| BP | GO:0010634 | positive regulation of epithelial cell migration | 0.009656 | 0.02871 | VEGFA/FGF2/KDR | 3 |
| BP | GO:0035306 | positive regulation of dephosphorylation | 0.009762 | 0.028953 | ADORA1/JAK2 | 2 |
| BP | GO:0061900 | glial cell activation | 0.009762 | 0.028953 | TNF/JAK2 | 2 |
| BP | GO:0032874 | positive regulation of stress-activated MAPK cascade | 0.00981 | 0.029057 | TNF/VEGFA/OPRK1 | 3 |
| BP | GO:0070304 | positive regulation of stress-activated protein kinase signaling cascade | 0.009965 | 0.02948 | TNF/VEGFA/OPRK1 | 3 |
| BP | GO:0001658 | branching involved in ureteric bud morphogenesis | 0.010088 | 0.02962 | VEGFA/FGF2 | 2 |
| BP | GO:0001885 | endothelial cell development | 0.010088 | 0.02962 | TNF/VEGFA | 2 |
| BP | GO:0033619 | membrane protein proteolysis | 0.010088 | 0.02962 | TNF/ADRA2A | 2 |
| BP | GO:0043388 | positive regulation of DNA binding | 0.010088 | 0.02962 | PPARG/JAK2 | 2 |
| BP | GO:0045824 | negative regulation of innate immune response | 0.010088 | 0.02962 | DRD2/PPARG | 2 |
| BP | GO:2000351 | regulation of endothelial cell apoptotic process | 0.010088 | 0.02962 | TNF/KDR | 2 |
| BP | GO:0010921 | regulation of phosphatase activity | 0.01028 | 0.030145 | TNF/DRD2/JAK2 | 3 |
| BP | GO:0051893 | regulation of focal adhesion assembly | 0.010419 | 0.030439 | VEGFA/KDR | 2 |
| BP | GO:0090109 | regulation of cell-substrate junction assembly | 0.010419 | 0.030439 | VEGFA/KDR | 2 |
| BP | GO:0150116 | regulation of cell-substrate junction organization | 0.010419 | 0.030439 | VEGFA/KDR | 2 |
| BP | GO:0016042 | lipid catabolic process | 0.010438 | 0.030457 | TNF/ADRA2A/ADORA1/CYP19A1 | 4 |
| BP | GO:0001959 | regulation of cytokine-mediated signaling pathway | 0.010601 | 0.030815 | TNF/PPARG/JAK2 | 3 |
| BP | GO:0007416 | synapse assembly | 0.010601 | 0.030815 | DRD2/ACHE/DRD1 | 3 |
| BP | GO:0048469 | cell maturation | 0.010601 | 0.030815 | VEGFA/REN/PPARG | 3 |
| BP | GO:0007405 | neuroblast proliferation | 0.010755 | 0.031172 | DRD2/VEGFA | 2 |
| BP | GO:1905953 | negative regulation of lipid localization | 0.010755 | 0.031172 | TNF/PPARG | 2 |
| BP | GO:0010950 | positive regulation of endopeptidase activity | 0.010763 | 0.031172 | TNF/PPARG/JAK2 | 3 |
| BP | GO:0051099 | positive regulation of binding | 0.010927 | 0.031607 | ACE/PPARG/JAK2 | 3 |
| BP | GO:0022408 | negative regulation of cell-cell adhesion | 0.011092 | 0.031858 | VEGFA/IL2/JAK2 | 3 |
| BP | GO:0071466 | cellular response to xenobiotic stimulus | 0.011092 | 0.031858 | OPRM1/BCHE/NR3C1 | 3 |
| BP | GO:1905330 | regulation of morphogenesis of an epithelium | 0.011092 | 0.031858 | TNF/VEGFA/ESR1 | 3 |
| BP | GO:0007585 | respiratory gaseous exchange by respiratory system | 0.011095 | 0.031858 | CHRNA4/ADORA1 | 2 |
| BP | GO:0051898 | negative regulation of protein kinase B signaling | 0.011095 | 0.031858 | DRD2/DRD3 | 2 |
| BP | GO:1902305 | regulation of sodium ion transmembrane transport | 0.011095 | 0.031858 | DRD4/DRD3 | 2 |
| BP | GO:0002637 | regulation of immunoglobulin production | 0.01144 | 0.032729 | TNF/IL2 | 2 |
| BP | GO:0042982 | amyloid precursor protein metabolic process | 0.01144 | 0.032729 | TNF/ACHE | 2 |
| BP | GO:0046530 | photoreceptor cell differentiation | 0.01144 | 0.032729 | TH/VEGFA | 2 |
| BP | GO:0002040 | sprouting angiogenesis | 0.011597 | 0.033137 | VEGFA/FGF2/KDR | 3 |
| BP | GO:0010721 | negative regulation of cell development | 0.011653 | 0.033256 | TNF/SLC6A4/VEGFA/DRD3 | 4 |
| BP | GO:1900449 | regulation of glutamate receptor signaling pathway | 0.01179 | 0.033606 | OPRM1/ADRB2 | 2 |
| BP | GO:0045216 | cell-cell junction organization | 0.011941 | 0.033996 | TNF/VEGFA/ACE | 3 |
| BP | GO:0070997 | neuron death | 0.012117 | 0.034408 | TNF/PIK3CA/ADORA1/JAK2 | 4 |
| BP | GO:0032729 | positive regulation of interferon-gamma production | 0.012145 | 0.034408 | TNF/IL2 | 2 |
| BP | GO:0060675 | ureteric bud morphogenesis | 0.012145 | 0.034408 | VEGFA/FGF2 | 2 |
| BP | GO:0072577 | endothelial cell apoptotic process | 0.012145 | 0.034408 | TNF/KDR | 2 |
| BP | GO:0006367 | transcription initiation from RNA polymerase II promoter | 0.012468 | 0.035212 | ESR1/NR3C1/PPARG | 3 |
| BP | GO:0051052 | regulation of DNA metabolic process | 0.012472 | 0.035212 | PIK3CA/FGF2/IL2/PPARG | 4 |
| BP | GO:0045428 | regulation of nitric oxide biosynthetic process | 0.012504 | 0.035212 | TNF/JAK2 | 2 |
| BP | GO:0072171 | mesonephric tubule morphogenesis | 0.012504 | 0.035212 | VEGFA/FGF2 | 2 |
| BP | GO:0099072 | regulation of postsynaptic membrane neurotransmitter receptor levels | 0.012504 | 0.035212 | DRD4/DRD3 | 2 |
| BP | GO:0060759 | regulation of response to cytokine stimulus | 0.012827 | 0.036078 | TNF/PPARG/JAK2 | 3 |
| BP | GO:0042108 | positive regulation of cytokine biosynthetic process | 0.012868 | 0.03615 | TNF/JAK2 | 2 |
| BP | GO:0016239 | positive regulation of macroautophagy | 0.013236 | 0.036963 | ADRB2/KDR | 2 |
| BP | GO:0019915 | lipid storage | 0.013236 | 0.036963 | TNF/PPARG | 2 |
| BP | GO:0035924 | cellular response to vascular endothelial growth factor stimulus | 0.013236 | 0.036963 | VEGFA/KDR | 2 |
| BP | GO:0046323 | glucose import | 0.013236 | 0.036963 | TNF/DRD1 | 2 |
| BP | GO:0071230 | cellular response to amino acid stimulus | 0.013236 | 0.036963 | TNF/MMP2 | 2 |
| BP | GO:0045862 | positive regulation of proteolysis | 0.013327 | 0.037173 | TNF/ADRA2A/PPARG/JAK2 | 4 |
| BP | GO:1901654 | response to ketone | 0.013376 | 0.037264 | TH/NR3C1/PPARG | 3 |
| BP | GO:0050766 | positive regulation of phagocytosis | 0.013609 | 0.037824 | TNF/PPARG | 2 |
| BP | GO:0071300 | cellular response to retinoic acid | 0.013609 | 0.037824 | SLC6A4/PPARG | 2 |
| BP | GO:2000377 | regulation of reactive oxygen species metabolic process | 0.013749 | 0.038168 | TNF/SLC18A2/JAK2 | 3 |
| BP | GO:0001654 | eye development | 0.013832 | 0.038308 | TH/VEGFA/ACHE/HDAC1 | 4 |
| BP | GO:0010038 | response to metal ion | 0.013832 | 0.038308 | TH/DRD2/MAPK8/SLC6A3 | 4 |
| BP | GO:0010952 | positive regulation of peptidase activity | 0.014128 | 0.039082 | TNF/PPARG/JAK2 | 3 |
| BP | GO:0046890 | regulation of lipid biosynthetic process | 0.01432 | 0.039566 | TNF/HTR2A/HTR2C | 3 |
| BP | GO:0150063 | visual system development | 0.014349 | 0.0396 | TH/VEGFA/ACHE/HDAC1 | 4 |
| BP | GO:0030198 | extracellular matrix organization | 0.014612 | 0.040278 | TNF/FGF2/KDR/MMP2 | 4 |
| BP | GO:0006909 | phagocytosis | 0.014745 | 0.040387 | TNF/PIK3CA/PPARG/ADORA1 | 4 |
| BP | GO:0043062 | extracellular structure organization | 0.014745 | 0.040387 | TNF/FGF2/KDR/MMP2 | 4 |
| BP | GO:0019226 | transmission of nerve impulse | 0.014755 | 0.040387 | DRD1/DRD5 | 2 |
| BP | GO:0038034 | signal transduction in absence of ligand | 0.014755 | 0.040387 | TNF/IL2 | 2 |
| BP | GO:0061180 | mammary gland epithelium development | 0.014755 | 0.040387 | ESR1/JAK2 | 2 |
| BP | GO:0097192 | extrinsic apoptotic signaling pathway in absence of ligand | 0.014755 | 0.040387 | TNF/IL2 | 2 |
| BP | GO:0045666 | positive regulation of neuron differentiation | 0.015013 | 0.040996 | VEGFA/ADRA2C/ADRA2B/IL2 | 4 |
| BP | GO:0048880 | sensory system development | 0.015013 | 0.040996 | TH/VEGFA/ACHE/HDAC1 | 4 |
| BP | GO:0002573 | myeloid leukocyte differentiation | 0.015502 | 0.042195 | TNF/VEGFA/PPARG | 3 |
| BP | GO:0045766 | positive regulation of angiogenesis | 0.015502 | 0.042195 | VEGFA/FGF2/KDR | 3 |
| BP | GO:0072078 | nephron tubule morphogenesis | 0.015542 | 0.042195 | VEGFA/FGF2 | 2 |
| BP | GO:0099601 | regulation of neurotransmitter receptor activity | 0.015542 | 0.042195 | OPRM1/ADRB2 | 2 |
| BP | GO:1901616 | organic hydroxy compound catabolic process | 0.015542 | 0.042195 | MAOA/SLC6A3 | 2 |
| BP | GO:0071222 | cellular response to lipopolysaccharide | 0.015705 | 0.042588 | TNF/MAPK8/OPRK1 | 3 |
| BP | GO:0050679 | positive regulation of epithelial cell proliferation | 0.015908 | 0.043031 | VEGFA/FGF2/KDR | 3 |
| BP | GO:0021536 | diencephalon development | 0.015941 | 0.043031 | DRD2/SLC6A3 | 2 |
| BP | GO:0033143 | regulation of intracellular steroid hormone receptor signaling pathway | 0.015941 | 0.043031 | ESR1/HDAC1 | 2 |
| BP | GO:0150076 | neuroinflammatory response | 0.015941 | 0.043031 | TNF/JAK2 | 2 |
| BP | GO:0002064 | epithelial cell development | 0.016114 | 0.043446 | TNF/VEGFA/ESR1 | 3 |
| BP | GO:0001937 | negative regulation of endothelial cell proliferation | 0.016346 | 0.04392 | TNF/PPARG | 2 |
| BP | GO:0007492 | endoderm development | 0.016346 | 0.04392 | MMP2/HDAC1 | 2 |
| BP | GO:0072088 | nephron epithelium morphogenesis | 0.016346 | 0.04392 | VEGFA/FGF2 | 2 |
| BP | GO:0045765 | regulation of angiogenesis | 0.016684 | 0.044778 | VEGFA/FGF2/PPARG/KDR | 4 |
| BP | GO:0043523 | regulation of neuron apoptotic process | 0.016738 | 0.044812 | TNF/PIK3CA/JAK2 | 3 |
| BP | GO:0006809 | nitric oxide biosynthetic process | 0.016754 | 0.044812 | TNF/JAK2 | 2 |
| BP | GO:0048678 | response to axon injury | 0.016754 | 0.044812 | DRD2/JAK2 | 2 |
| BP | GO:1901653 | cellular response to peptide | 0.016974 | 0.045347 | PIK3CA/ADRB2/PPARG/JAK2 | 4 |
| BP | GO:0071219 | cellular response to molecule of bacterial origin | 0.017162 | 0.045656 | TNF/MAPK8/OPRK1 | 3 |
| BP | GO:0021954 | central nervous system neuron development | 0.017167 | 0.045656 | DRD2/DRD1 | 2 |
| BP | GO:0061333 | renal tubule morphogenesis | 0.017167 | 0.045656 | VEGFA/FGF2 | 2 |
| BP | GO:0072028 | nephron morphogenesis | 0.017167 | 0.045656 | VEGFA/FGF2 | 2 |
| BP | GO:0001570 | vasculogenesis | 0.017585 | 0.04666 | VEGFA/KDR | 2 |
| BP | GO:0043537 | negative regulation of blood vessel endothelial cell migration | 0.017585 | 0.04666 | FGF2/PPARG | 2 |
| BP | GO:0010810 | regulation of cell-substrate adhesion | 0.01781 | 0.047204 | VEGFA/KDR/JAK2 | 3 |
| BP | GO:0043393 | regulation of protein binding | 0.018249 | 0.048313 | ACE/MAPK8/ADRB2 | 3 |
| BP | GO:0014855 | striated muscle cell proliferation | 0.018432 | 0.048634 | FGF2/JAK2 | 2 |
| BP | GO:0048041 | focal adhesion assembly | 0.018432 | 0.048634 | VEGFA/KDR | 2 |
| BP | GO:0048708 | astrocyte differentiation | 0.018432 | 0.048634 | TNF/DRD1 | 2 |
| BP | GO:1903039 | positive regulation of leukocyte cell-cell adhesion | 0.018471 | 0.04868 | TNF/PIK3CA/IL2 | 3 |
| BP | GO:0007584 | response to nutrient | 0.018694 | 0.049213 | SLC6A4/BCHE/PPARG | 3 |
| BP | GO:0046209 | nitric oxide metabolic process | 0.018863 | 0.049602 | TNF/JAK2 | 2 |
| BP | GO:0001503 | ossification | 0.018932 | 0.04973 | TNF/ACHE/ADRB2/MMP2 | 4 |
| CC | GO:0099699 | integral component of synaptic membrane | 8.76E-21 | 1.35E-18 | SLC6A4/DRD2/CHRNA4/OPRM1/HTR2A/ADRA2C/DRD1/SLC6A3/ADRA1A/DRD3/HTR5A/OPRK1/CHRM1/HTR1B/ADORA1 | 15 |
| CC | GO:0099240 | intrinsic component of synaptic membrane | 2.83E-20 | 2.18E-18 | SLC6A4/DRD2/CHRNA4/OPRM1/HTR2A/ADRA2C/DRD1/SLC6A3/ADRA1A/DRD3/HTR5A/OPRK1/CHRM1/HTR1B/ADORA1 | 15 |
| CC | GO:0099056 | integral component of presynaptic membrane | 1.61E-19 | 8.26E-18 | SLC6A4/DRD2/CHRNA4/OPRM1/HTR2A/DRD1/SLC6A3/ADRA1A/OPRK1/CHRM1/HTR1B/ADORA1 | 12 |
| CC | GO:0098889 | intrinsic component of presynaptic membrane | 7.14E-19 | 2.75E-17 | SLC6A4/DRD2/CHRNA4/OPRM1/HTR2A/DRD1/SLC6A3/ADRA1A/OPRK1/CHRM1/HTR1B/ADORA1 | 12 |
| CC | GO:0099055 | integral component of postsynaptic membrane | 9.24E-19 | 2.84E-17 | SLC6A4/DRD2/OPRM1/HTR2A/ADRA2C/DRD1/SLC6A3/ADRA1A/DRD3/HTR5A/OPRK1/CHRM1/ADORA1 | 13 |
| CC | GO:0098936 | intrinsic component of postsynaptic membrane | 1.63E-18 | 4.17E-17 | SLC6A4/DRD2/OPRM1/HTR2A/ADRA2C/DRD1/SLC6A3/ADRA1A/DRD3/HTR5A/OPRK1/CHRM1/ADORA1 | 13 |
| CC | GO:0042734 | presynaptic membrane | 6.61E-17 | 1.46E-15 | SLC6A4/DRD2/CHRNA4/OPRM1/HTR2A/SLC6A2/DRD1/SLC6A3/ADRA1A/OPRK1/CHRM1/HTR1B/ADORA1 | 13 |
| CC | GO:0097060 | synaptic membrane | 2.54E-15 | 4.89E-14 | SLC6A4/DRD2/CHRNA4/OPRM1/HTR2A/SLC6A2/ADRA2C/DRD1/SLC6A3/ADRA1A/DRD3/HTR5A/OPRK1/CHRM1/HTR1B/ADORA1 | 16 |
| CC | GO:0098793 | presynapse | 1.86E-14 | 3.18E-13 | TH/SLC6A4/DRD2/CHRNA4/OPRM1/HTR2A/SLC6A2/ADRA2C/DRD1/SLC18A2/SLC6A3/ADRA1A/OPRK1/CHRM1/HTR1B/ADORA1 | 16 |
| CC | GO:0045211 | postsynaptic membrane | 2.21E-14 | 3.41E-13 | SLC6A4/DRD2/CHRNA4/OPRM1/HTR2A/ADRA2C/DRD1/SLC6A3/ADRA1A/DRD3/HTR5A/OPRK1/CHRM1/ADORA1 | 14 |
| CC | GO:0098691 | dopaminergic synapse | 2.55E-13 | 3.57E-12 | DRD2/CHRNA4/SLC18A2/SLC6A3/ADRA1A/DRD3 | 6 |
| CC | GO:0043679 | axon terminus | 4.28E-10 | 5.49E-09 | TH/DRD2/ADRA2C/SLC18A2/OPRK1/CHRM1/HTR1B/ADORA1 | 8 |
| CC | GO:0044306 | neuron projection terminus | 1.4E-09 | 1.66E-08 | TH/DRD2/ADRA2C/SLC18A2/OPRK1/CHRM1/HTR1B/ADORA1 | 8 |
| CC | GO:0043025 | neuronal cell body | 1.76E-09 | 1.94E-08 | TH/DRD2/CHRNA4/OPRM1/HTR2A/SLC6A2/ADRA2C/SLC6A3/HTR5A/OPRK1/ADORA1/HDAC1 | 12 |
| CC | GO:0045121 | membrane raft | 3.59E-09 | 3.56E-08 | TNF/SLC6A4/OPRM1/HTR2A/ADRA1B/SLC6A2/SLC6A3/ADRA1A/KDR/JAK2 | 10 |
| CC | GO:0098857 | membrane microdomain | 3.7E-09 | 3.56E-08 | TNF/SLC6A4/OPRM1/HTR2A/ADRA1B/SLC6A2/SLC6A3/ADRA1A/KDR/JAK2 | 10 |
| CC | GO:0098589 | membrane region | 5.29E-09 | 4.79E-08 | TNF/SLC6A4/OPRM1/HTR2A/ADRA1B/SLC6A2/SLC6A3/ADRA1A/KDR/JAK2 | 10 |
| CC | GO:0098978 | glutamatergic synapse | 0.00000014 | 0.0000012 | DRD2/DRD4/HTR2A/ADRA2C/DRD1/ADRA1A/DRD3/CHRM1/JAK2 | 9 |
| CC | GO:0150034 | distal axon | 0.000000395 | 0.0000032 | TH/DRD2/ADRA2C/SLC18A2/OPRK1/CHRM1/HTR1B/ADORA1 | 8 |
| CC | GO:0005901 | caveola | 0.00000145 | 0.0000111 | HTR2A/ADRA1B/SLC6A3/ADRA1A/JAK2 | 5 |
| CC | GO:0044853 | plasma membrane raft | 0.00000667 | 0.0000489 | HTR2A/ADRA1B/SLC6A3/ADRA1A/JAK2 | 5 |
| CC | GO:0043204 | perikaryon | 0.0000182 | 0.000128 | TH/DRD2/OPRM1/HTR5A/OPRK1 | 5 |
| CC | GO:0098982 | GABA-ergic synapse | 0.0000266 | 0.000178 | DRD2/DRD1/ADRA1A/DRD3 | 4 |
| CC | GO:0099060 | integral component of postsynaptic specialization membrane | 0.0000314 | 0.000201 | ADRA2C/DRD3/HTR5A/CHRM1 | 4 |
| CC | GO:0098948 | intrinsic component of postsynaptic specialization membrane | 0.0000367 | 0.000226 | ADRA2C/DRD3/HTR5A/CHRM1 | 4 |
| CC | GO:0099634 | postsynaptic specialization membrane | 0.000106 | 0.000629 | ADRA2C/DRD3/HTR5A/CHRM1 | 4 |
| CC | GO:0099572 | postsynaptic specialization | 0.00019 | 0.001085 | DRD2/ADRA2C/DRD3/HTR5A/CHRM1/ADORA1 | 6 |
| CC | GO:0099061 | integral component of postsynaptic density membrane | 0.000245 | 0.001347 | ADRA2C/DRD3/CHRM1 | 3 |
| CC | GO:0043195 | terminal bouton | 0.00026 | 0.001379 | TH/SLC18A2/ADORA1 | 3 |
| CC | GO:0099146 | intrinsic component of postsynaptic density membrane | 0.000291 | 0.001495 | ADRA2C/DRD3/CHRM1 | 3 |
| CC | GO:0043197 | dendritic spine | 0.000601 | 0.002986 | DRD2/OPRM1/DRD1/ADORA1 | 4 |
| CC | GO:0044309 | neuron spine | 0.00063 | 0.003032 | DRD2/OPRM1/DRD1/ADORA1 | 4 |
| CC | GO:0060170 | ciliary membrane | 0.000718 | 0.003349 | DRD2/DRD1/DRD5 | 3 |
| CC | GO:0098839 | postsynaptic density membrane | 0.000777 | 0.003521 | ADRA2C/DRD3/CHRM1 | 3 |
| CC | GO:0014069 | postsynaptic density | 0.001115 | 0.004823 | DRD2/ADRA2C/DRD3/CHRM1/ADORA1 | 5 |
| CC | GO:0031253 | cell projection membrane | 0.001146 | 0.004823 | DRD2/OPRM1/DRD1/DRD5/ADORA1 | 5 |
| CC | GO:0032279 | asymmetric synapse | 0.001177 | 0.004823 | DRD2/ADRA2C/DRD3/CHRM1/ADORA1 | 5 |
| CC | GO:0008021 | synaptic vesicle | 0.00119 | 0.004823 | TH/DRD2/SLC18A2/OPRK1 | 4 |
| CC | GO:0044305 | calyx of Held | 0.001548 | 0.006009 | HTR1B/ADORA1 | 2 |
| CC | GO:0098984 | neuron to neuron synapse | 0.001568 | 0.006009 | DRD2/ADRA2C/DRD3/CHRM1/ADORA1 | 5 |
| CC | GO:0070382 | exocytic vesicle | 0.0016 | 0.006009 | TH/DRD2/SLC18A2/OPRK1 | 4 |
| CC | GO:0030672 | synaptic vesicle membrane | 0.002075 | 0.007433 | DRD2/SLC18A2/OPRK1 | 3 |
| CC | GO:0099501 | exocytic vesicle membrane | 0.002075 | 0.007433 | DRD2/SLC18A2/OPRK1 | 3 |
| CC | GO:0097730 | non-motile cilium | 0.005704 | 0.019966 | DRD2/DRD1/DRD5 | 3 |
| CC | GO:0032589 | neuron projection membrane | 0.008505 | 0.029107 | OPRM1/ADORA1 | 2 |
| CC | GO:0030658 | transport vesicle membrane | 0.014127 | 0.047296 | DRD2/SLC18A2/OPRK1 | 3 |
| CC | GO:0030133 | transport vesicle | 0.015045 | 0.049296 | TH/DRD2/SLC18A2/OPRK1 | 4 |
| MF | GO:0008227 | G protein-coupled amine receptor activity | 9.88E-34 | 2.59E-31 | DRD2/HTR1A/DRD4/HTR2A/ADRA1B/ADRA2A/ADRA2C/ADRA1A/ADRA1D/ADRB2/DRD3/ADRA2B/HTR5A/CHRM1/HTR2C/HTR1B/ADRB1/HTR1D | 18 |
| MF | GO:1901338 | catecholamine binding | 4.41E-27 | 5.77E-25 | TH/DRD2/DRD4/ADRA2A/ADRA2C/DRD1/SLC6A3/ADRB2/DRD3/ADRA2B/DRD5/ADRB1 | 12 |
| MF | GO:0030594 | neurotransmitter receptor activity | 8.7E-24 | 7.6E-22 | DRD2/CHRNA4/OPRM1/HTR1A/DRD4/HTR2A/DRD1/DRD3/HTR5A/CHRM1/DRD5/HTR2C/HTR1B/ADORA1/ADRB1/HTR1D | 16 |
| MF | GO:0004935 | adrenergic receptor activity | 1.52E-22 | 9.93E-21 | DRD2/ADRA1B/ADRA2A/ADRA2C/ADRA1A/ADRA1D/ADRB2/DRD3/ADRA2B/ADRB1 | 10 |
| MF | GO:0099528 | G protein-coupled neurotransmitter receptor activity | 8.34E-18 | 4.37E-16 | OPRM1/HTR1A/DRD4/HTR2A/HTR5A/CHRM1/HTR2C/HTR1B/ADRB1/HTR1D | 10 |
| MF | GO:0035240 | dopamine binding | 1.16E-15 | 5.06E-14 | TH/DRD2/DRD4/DRD1/SLC6A3/DRD3/DRD5 | 7 |
| MF | GO:0042165 | neurotransmitter binding | 2.08E-14 | 7.78E-13 | SLC6A4/ACHE/CHRNA4/HTR1A/HTR2A/HTR5A/BCHE/HTR2C/HTR1B | 9 |
| MF | GO:0004993 | G protein-coupled serotonin receptor activity | 2.73E-14 | 7.95E-13 | HTR1A/DRD4/HTR2A/HTR5A/CHRM1/HTR2C/HTR1B/HTR1D | 8 |
| MF | GO:0099589 | serotonin receptor activity | 2.73E-14 | 7.95E-13 | HTR1A/DRD4/HTR2A/HTR5A/CHRM1/HTR2C/HTR1B/HTR1D | 8 |
| MF | GO:0043176 | amine binding | 6E-14 | 1.43E-12 | SLC6A4/HTR1A/HTR2A/HTR5A/HTR2C/HTR1B | 6 |
| MF | GO:0051378 | serotonin binding | 6E-14 | 1.43E-12 | SLC6A4/HTR1A/HTR2A/HTR5A/HTR2C/HTR1B | 6 |
| MF | GO:0098960 | postsynaptic neurotransmitter receptor activity | 1.32E-11 | 2.88E-10 | DRD2/CHRNA4/DRD4/DRD1/DRD3/CHRM1/DRD5/ADRB1 | 8 |
| MF | GO:0004952 | dopamine neurotransmitter receptor activity | 2.96E-11 | 5.96E-10 | DRD2/DRD4/DRD1/DRD3/DRD5 | 5 |
| MF | GO:0015378 | sodium:chloride symporter activity | 9.89E-09 | 0.000000185 | SLC6A4/SLC6A2/SLC18A2/SLC6A3 | 4 |
| MF | GO:0015373 | anion:sodium symporter activity | 3.35E-08 | 0.000000584 | SLC6A4/SLC6A2/SLC18A2/SLC6A3 | 4 |
| MF | GO:0015377 | cation:chloride symporter activity | 8.47E-08 | 0.00000139 | SLC6A4/SLC6A2/SLC18A2/SLC6A3 | 4 |
| MF | GO:0015296 | anion:cation symporter activity | 0.000000179 | 0.00000276 | SLC6A4/SLC6A2/SLC18A2/SLC6A3 | 4 |
| MF | GO:0001965 | G-protein alpha-subunit binding | 0.000000486 | 0.00000708 | OPRM1/HTR2A/DRD1/DRD5 | 4 |
| MF | GO:0005328 | neurotransmitter:sodium symporter activity | 0.000000682 | 0.0000094 | SLC6A4/SLC6A2/SLC18A2/SLC6A3 | 4 |
| MF | GO:0001664 | G protein-coupled receptor binding | 0.000000775 | 0.0000102 | ACE/ADRA2A/ADRA2C/DRD3/IL2/ADORA1/ADRB1/JAK2 | 8 |
| MF | GO:1901618 | organic hydroxy compound transmembrane transporter activity | 0.00000597 | 0.0000745 | SLC6A4/SLC6A2/SLC18A2/SLC6A3 | 4 |
| MF | GO:0005326 | neurotransmitter transmembrane transporter activity | 0.0000108 | 0.000129 | SLC6A4/SLC6A2/SLC18A2/SLC6A3 | 4 |
| MF | GO:0031690 | adrenergic receptor binding | 0.0000176 | 0.000201 | ADRA2A/ADRA2C/ADRB1 | 3 |
| MF | GO:0015370 | solute:sodium symporter activity | 0.0000452 | 0.000493 | SLC6A4/SLC6A2/SLC18A2/SLC6A3 | 4 |
| MF | GO:0042277 | peptide binding | 0.000139 | 0.001456 | ACHE/OPRM1/ADRB2/OPRK1/BCHE/PPARG | 6 |
| MF | GO:0015108 | chloride transmembrane transporter activity | 0.000154 | 0.001555 | SLC6A4/SLC6A2/SLC18A2/SLC6A3 | 4 |
| MF | GO:0015294 | solute:cation symporter activity | 0.000173 | 0.001679 | SLC6A4/SLC6A2/SLC18A2/SLC6A3 | 4 |
| MF | GO:0004879 | nuclear receptor activity | 0.000279 | 0.002523 | ESR1/NR3C1/PPARG | 3 |
| MF | GO:0098531 | ligand-activated transcription factor activity | 0.000279 | 0.002523 | ESR1/NR3C1/PPARG | 3 |
| MF | GO:0099529 | neurotransmitter receptor activity involved in regulation of postsynaptic membrane potential | 0.000336 | 0.002931 | CHRNA4/CHRM1/ADRB1 | 3 |
| MF | GO:0033218 | amide binding | 0.000382 | 0.003194 | ACHE/OPRM1/ADRB2/OPRK1/BCHE/PPARG | 6 |
| MF | GO:0043560 | insulin receptor substrate binding | 0.00039 | 0.003194 | PIK3CA/JAK2 | 2 |
| MF | GO:0003707 | steroid hormone receptor activity | 0.000469 | 0.003641 | ESR1/NR3C1/PPARG | 3 |
| MF | GO:0070851 | growth factor receptor binding | 0.000472 | 0.003641 | VEGFA/FGF2/IL2/JAK2 | 4 |
| MF | GO:0015103 | inorganic anion transmembrane transporter activity | 0.000588 | 0.004401 | SLC6A4/SLC6A2/SLC18A2/SLC6A3 | 4 |
| MF | GO:0015293 | symporter activity | 0.00062 | 0.00451 | SLC6A4/SLC6A2/SLC18A2/SLC6A3 | 4 |
| MF | GO:0015081 | sodium ion transmembrane transporter activity | 0.000722 | 0.005114 | SLC6A4/SLC6A2/SLC18A2/SLC6A3 | 4 |
| MF | GO:0042166 | acetylcholine binding | 0.000955 | 0.006582 | ACHE/CHRNA4 | 2 |
| MF | GO:0046873 | metal ion transmembrane transporter activity | 0.001144 | 0.007687 | SLC6A4/OPRM1/SLC6A2/SLC18A2/SLC6A3/HTR1B | 6 |
| MF | GO:0001540 | amyloid-beta binding | 0.001235 | 0.008092 | ACHE/ADRB2/BCHE | 3 |
| MF | GO:0015464 | acetylcholine receptor activity | 0.001758 | 0.011232 | CHRNA4/CHRM1 | 2 |
| MF | GO:0042923 | neuropeptide binding | 0.001914 | 0.011941 | OPRM1/OPRK1 | 2 |
| MF | GO:0042562 | hormone binding | 0.002664 | 0.016233 | ACHE/CHRNA4/NR3C1 | 3 |
| MF | GO:0005125 | cytokine activity | 0.002948 | 0.017553 | TNF/VEGFA/FGF2/IL2 | 4 |
| MF | GO:0022853 | active ion transmembrane transporter activity | 0.003789 | 0.02191 | SLC6A4/SLC6A2/SLC18A2/SLC6A3 | 4 |
| MF | GO:0015291 | secondary active transmembrane transporter activity | 0.003847 | 0.02191 | SLC6A4/SLC6A2/SLC18A2/SLC6A3 | 4 |
| MF | GO:0001103 | RNA polymerase II repressing transcription factor binding | 0.004049 | 0.022457 | PPARG/HDAC1 | 2 |
| MF | GO:0031072 | heat shock protein binding | 0.004114 | 0.022457 | NR3C1/ADORA1/KDR | 3 |
| MF | GO:0019825 | oxygen binding | 0.00428 | 0.022883 | TH/CYP19A1 | 2 |
| MF | GO:0042056 | chemoattractant activity | 0.004759 | 0.024938 | VEGFA/FGF2 | 2 |
| MF | GO:0051879 | Hsp90 protein binding | 0.005523 | 0.028371 | NR3C1/KDR | 2 |
| MF | GO:0030331 | estrogen receptor binding | 0.005789 | 0.029166 | ESR1/PPARG | 2 |
| MF | GO:0005126 | cytokine receptor binding | 0.00744 | 0.036418 | TNF/VEGFA/IL2/JAK2 | 4 |
| MF | GO:0005245 | voltage-gated calcium channel activity | 0.007506 | 0.036418 | OPRM1/HTR1B | 2 |
| MF | GO:0070888 | E-box binding | 0.008123 | 0.038697 | PPARG/HDAC1 | 2 |
| MF | GO:0015459 | potassium channel regulator activity | 0.008763 | 0.040998 | DRD4/ADRB2 | 2 |
| MF | GO:0008083 | growth factor activity | 0.009798 | 0.045039 | VEGFA/FGF2/IL2 | 3 |
| MF | GO:0043621 | protein self-association | 0.010107 | 0.045658 | ACHE/PPARG | 2 |
| MF | GO:0050840 | extracellular matrix binding | 0.010457 | 0.046436 | VEGFA/ACHE | 2 |
