# Supplementary material for: Exploration of Ziziphi Spinosae Semen in Treating Insomnia Based on Network Pharmacology Strategy
Source: Evid Based Complement Alternat Med. 2021 Oct 28;2021:9888607. doi: 10.1155/2021/9888607 (PMC8568550; doi:10.1155/2021/9888607)
Supplement: Supplementary Materials — Table S1: detailed information of related targets and gene symbols of compounds in ZSS. Table S2: the known therapeutic gene targets for insomnia. Table S3: detailed information about the D-C-G-D network. Table S4: overlapping gene symbols and corresponding degree values between disease and drug. Table S5: details of GO enrichment analyses. Table S6: details of KEGG pathway enrichment analyses. [file 9888607.f1.zip › 9888607.f1/Supplementary Table S6-Details of KEGG pathway enrichment analyses (1).doc]

| **Details of KEGG pathway enrichment analyses** | | | | | |
| --- | --- | --- | --- | --- | --- |
| **GO ID** | **GOTerm** | **Term PValue** | **Term PValue Corrected with Bonferroni step down** | **GOGroups** | **Associated Genes Found** |
| KEGG:04080 | Neuroactive ligand-receptor interaction | 1.26738E-22 | 2.91497E-21 | Group6 | [ADORA1, ADRA1A, ADRA1B, ADRA1D, ADRA2A, ADRA2B, ADRA2C, ADRB1, ADRB2, CHRM1, CHRNA4, DRD1, DRD2, DRD3, DRD4, DRD5, HTR1A, HTR1B, HTR1D, HTR2A, HTR2C, HTR5A, NR3C1, OPRK1, OPRM1] |
| KEGG:04024 | cAMP signaling pathway | 2.43215E-09 | 5.35073E-08 | Group3 | [ADORA1, ADRB1, ADRB2, CHRM1, DRD1, DRD2, DRD5, HTR1A, HTR1B, HTR1D, MAPK8, PIK3CA] |
| KEGG:04728 | Dopaminergic synapse | 3.1826E-09 | 6.68345E-08 | Group4 | [DRD1, DRD2, DRD3, DRD4, DRD5, MAOA, MAPK8, SLC18A2, SLC6A3, TH] |
| KEGG:04020 | Calcium signaling pathway | 9.59378E-09 | 1.91876E-07 | Group6 | [ADRA1A, ADRA1B, ADRA1D, ADRB1, ADRB2, CHRM1, DRD1, DRD5, HTR2A, HTR2C, HTR5A] |
| KEGG:04726 | Serotonergic synapse | 1.6073E-08 | 3.05386E-07 | Group2 | [HTR1A, HTR1B, HTR1D, HTR2A, HTR2C, HTR5A, MAOA, SLC18A2, SLC6A4] |
| KEGG:05030 | Cocaine addiction | 3.35948E-07 | 6.04707E-06 | Group4 | [DRD1, DRD2, MAOA, SLC18A2, SLC6A3, TH] |
| KEGG:04022 | cGMP-PKG signaling pathway | 4.08329E-07 | 6.9416E-06 | Group6 | [ADORA1, ADRA1A, ADRA1B, ADRA1D, ADRA2A, ADRA2B, ADRA2C, ADRB1, ADRB2] |
| KEGG:05031 | Amphetamine addiction | 2.63755E-06 | 4.22009E-05 | Group4 | [DRD1, HDAC1, MAOA, SLC18A2, SLC6A3, TH] |
| KEGG:04933 | AGE-RAGE signaling pathway in diabetic complications | 2.28855E-05 | 0.000343282 | Group5 | [JAK2, MAPK8, MMP2, PIK3CA, TNF, VEGFA] |
| KEGG:04917 | Prolactin signaling pathway | 5.0475E-05 | 0.000656175 | Group5 | [ESR1, JAK2, MAPK8, PIK3CA, TH] |
| KEGG:04924 | Renin secretion | 4.70748E-05 | 0.000659047 | Group3 | [ACE, ADORA1, ADRB1, ADRB2, REN] |
| KEGG:04540 | Gap junction | 0.000151031 | 0.001661345 | Group6 | [ADRB1, DRD1, DRD2, HTR2A, HTR2C] |
| KEGG:05418 | Fluid shear stress and atherosclerosis | 0.000145221 | 0.001742655 | Group5 | [KDR, MAPK8, MMP2, PIK3CA, TNF, VEGFA] |
| KEGG:04970 | Salivary secretion | 0.000176953 | 0.001769528 | Group6 | [ADRA1A, ADRA1B, ADRA1D, ADRB1, ADRB2] |
| KEGG:04923 | Regulation of lipolysis in adipocytes | 0.000303679 | 0.00242943 | Group3 | [ADORA1, ADRB1, ADRB2, PIK3CA] |
| KEGG:05142 | Chagas disease (American trypanosomiasis) | 0.000302077 | 0.00271869 | Group5 | [ACE, IL2, MAPK8, PIK3CA, TNF] |
| KEGG:04725 | Cholinergic synapse | 0.000485214 | 0.0033965 | Group1 | [ACHE, CHRM1, CHRNA4, JAK2, PIK3CA] |
| KEGG:04721 | Synaptic vesicle cycle | 0.001073492 | 0.00644095 | Group0 | [SLC18A2, SLC6A2, SLC6A3, SLC6A4] |
| KEGG:04920 | Adipocytokine signaling pathway | 0.007531409 | 0.007531409 | Group5 | [JAK2, MAPK8, TNF] |
